# Structural brain correlates of childhood trauma with replication across two large, independent community-based samples

**DOI:** 10.1192/j.eurpsy.2022.2347

**Published:** 2023-01-26

**Authors:** Rebecca A. Madden, Kimberley Atkinson, Xueyi Shen, Claire Green, Robert F. Hillary, Emma Hawkins, Emma Såge, Anca-Larisa Sandu, Gordon Waiter, Christopher McNeil, Mathew Harris, Archie Campbell, David Porteous, Jennifer A. Macfarlane, Alison Murray, Douglas Steele, Liana Romaniuk, Stephen M. Lawrie, Andrew M. McIntosh, Heather C. Whalley

**Affiliations:** 1 Division of Psychiatry, University of Edinburgh, Edinburgh, United Kingdom; 2 School of Medicine, University of Aberdeen, Aberdeen, United Kingdom; 3 Medical Sciences and Nutrition, School of Medicine, University of Dundee, Dundee, United Kingdom

**Keywords:** depression, MRI, trauma, adversity, brain

## Abstract

**Introduction:**

Childhood trauma and adversity are common across societies and have strong associations with physical and psychiatric morbidity throughout the life-course. One possible mechanism through which childhood trauma may predispose individuals to poor psychiatric outcomes is via associations with brain structure. This study aimed to elucidate the associations between childhood trauma and brain structure across two large, independent community cohorts.

**Methods:**

The two samples comprised (i) a subsample of Generation Scotland (n=1,024); and (ii) individuals from UK Biobank (n=27,202). This comprised n=28,226 for mega-analysis. MRI scans were processed using Free Surfer, providing cortical, subcortical, and global brain metrics. Regression models were used to determine associations between childhood trauma measures and brain metrics and psychiatric phenotypes.

**Results:**

Childhood trauma associated with lifetime depression across cohorts (OR 1.06 GS, 1.23 UKB), and related to early onset and recurrent course within both samples. There was evidence for associations between childhood trauma and structural brain metrics. This included reduced global brain volume, and reduced cortical surface area with highest effects in the frontal (β=−0.0385, SE=0.0048, p(FDR)=5.43x10−15) and parietal lobes (β=−0.0387, SE=0.005, p(FDR)=1.56x10−14). At a regional level the ventral diencephalon (VDc) displayed significant associations with childhood trauma measures across both cohorts and at mega-analysis (β=−0.0232, SE=0.0039, p(FDR)=2.91x10−8). There were also associations with reduced hippocampus, thalamus, and nucleus accumbens volumes.

**Discussion:**

Associations between childhood trauma and reduced global and regional brain volumes were found, across two independent UK cohorts, and at mega-analysis. This provides robust evidence for a lasting effect of childhood adversity on brain structure.

## Introduction

Adverse childhood experiences (ACEs) are reported to affect approximately 50% of the UK population [1, 2], with similar proportions worldwide [3]. The classification of ACEs varies, but typically includes abuse and neglect, witnessing domestic or neighborhood violence, family substance misuse, and parental divorce. ACEs have been found to act in a dose-dependent manner to raise the risk of a broad range of adverse outcomes in adulthood, including poor psychiatric outcomes [4]. The childhood trauma questionnaire (CTQ) describes a narrower range of early life experiences, focusing specifically on abuse and neglect, mainly in the home environment [5]. It has been repeatedly demonstrated that experiences that fall under this definition of “childhood trauma” (CT) have stronger associations with poor psychiatric outcomes than other types of childhood adversity (CA). It has been shown, for instance, that interpersonal trauma (adolescent sexual or physical abuse) is more strongly associated with lifetime structured clinical interview for DSM-IV (SCID) diagnoses than traumatic bereavement during adolescence [6]; and that physical neglect, emotional neglect, and sexual abuse show stronger associations with symptom severity among adult psychiatric inpatients than other ACEs such as peer bullying [7].

The biological underpinnings of the relationship between CA and psychiatric disorders are likely to be multifaceted, including chronic dysregulation of the hypothalamic–pituitary–adrenal (HPA) axis [8], disrupted attachment and emotional development [9], and deviations from typical brain development [10]. The latter hypothesis posits that the experience of trauma during sensitive periods of neurodevelopment may lead to structural abnormalities which predispose the adult individual to pathological symptoms [11]. A great deal of previous work has focused on describing the neurostructural consequences of CA; the literature has, however, proven to be inconsistent where results fail to replicate study to study.

The field to date has often taken a hypothesis-led approach; although there can be a strong rationale for examining specific regions of interest (ROIs) in relation to CA, this *a priori* approach can also contribute to bias and inconsistencies, whereby meaningful effects in other regions may be missed. The hippocampus, for instance, has received a lot of attention due to evidence for smaller hippocampal volumes in depression [12], alongside findings from preclinical work indicating the hippocampus is particularly vulnerable to early stressful life events [13]. These ROI studies generally find reduced hippocampal gray matter (GM) volume [14–20] in CA-exposed groups; however, other studies report no association [21–24]. A meta-analysis including 17 of these ROI studies looking specifically at the hippocampus did find evidence, however, for reduced volumes of the left, right, and total hippocampus (hedges *g* = −0.642, −0.616, and −0.517, respectively), despite significant between-study heterogeneity, and publication bias in the latter (Egger’s *t*[df = 33] = 3.44, *p* = 0.001) [25].

The amygdala has been another focus of ROI analyses of the neurostructural effects of CA, given its role in emotion and fear processing [19, 24, 26]. Two meta-analyses of amygdala-specific ROI analyses are however contradictory: one reporting reductions in amygdala volume from meta-analysis of 19 studies [25] and the other finding no effect in a meta-analysis of 15 studies [16]. Other commonly used ROIs have included the caudate nucleus [22, 23, 27], nucleus accumbens [21, 22], orbitofrontal cortex [18, 22], and anterior cingulate cortex [18, 22–24], with mixed findings.

Despite multiple strands of evidence that traumatic events can affect the structure and function of the thalamus, this region is rarely included as an ROI in *a priori* studies of CA. After traumatic events in adulthood, structural and functional changes have been observed in the thalamus [28–30]. Thalamic structural deviations after CA have also been observed [31, 32], and CT has been associated with functional hyperconnectivity of the thalamus in a transdiagnostic sample [33]. In addition to these structural and functional imaging findings, endocrine work has repeatedly shown evidence of dysregulation in the HPA axis in CT-exposed adult populations [34–37], implicating associated thalamic structures as well. The lack of inclusion of the thalamus and hypothalamus in *a priori* structural imaging studies of CA shows the potential to miss interesting regions by taking an ROI-based approach.

Although brain-wide (*a priori*) analyses have been conducted, they are rarer and often lack robust sample sizes. One such study revealed an association between CTQ scores and right insular surface area, [38]. Meta-analyses of voxel-based morphometry (VBM) approaches to brain-wide studies of CA report reduced GM volumes in the dorsolateral prefrontal cortex, right hippocampus, and right postcentral gyrus (across 19 studies with 1095 adult subjects [25]); and GM volume reductions in temporal, frontal, and midbrain regions, and the left postcentral gyrus (across 12 studies with a total of 693 children, adolescents, and adults [39]). The Enhancing NeuroImaging Genetics through Meta-Analysis (ENIGMA) Consortium [40] has recently produced larger-sample, *a priori* analyses of the impact of CA on adult brain structure based on meta-analyzed data from multiple cohort studies. In an analysis across seven subcortical GM structures defined by the FreeSurfer image processing software, no significant main effects of CA were found [41]. A similar approach taken in cortical thickness and surface area measures found the main effects of CA score in the thickness of the banks of the superior temporal sulcus and the supramarginal gyrus, and the surface area of the middle temporal gyrus [42]. They also found various interaction effects with age, sex, and major depressive disorder (MDD) diagnosis.

It is evident that there is limited consistency in determining the relationships between CT and adult structural imaging findings from whole-brain analyses. These inconsistencies may result from between-study variability. In particular, differences in the detail and definition of CT/adversity measures, in the demographics, and in the use of healthy or psychiatric populations (with varying diagnoses and severities of clinical status). With the growing opportunities provided by neuro-imaged population cohorts, large sample sizes are available within a single study protocol, enabling the analysis of neurostructural sequalae of CT more homogenous samples. In this study, therefore, we describe psychiatric phenotyping, whole-brain analysis, and regional analysis of CT in a subsample of the Generation Scotland: Scottish Family Health Study (GS) cohort with neuroimaging data. We based our initial analyses on this cohort due to its characteristically deep phenotyping, including the 28-item CTQ. We then sought to replicate in the larger UK Biobank (UKB) cohort, which utilized a much shorter questionnaire assessing CT. In addition, we report imaging mega-analysis from the combined cohorts, with a total sample size of *n* = 28,226. These large samples make the findings presented here well powered, and more generalizable to the population than smaller clinically oriented samples.

## Methods

**Figure 1.**
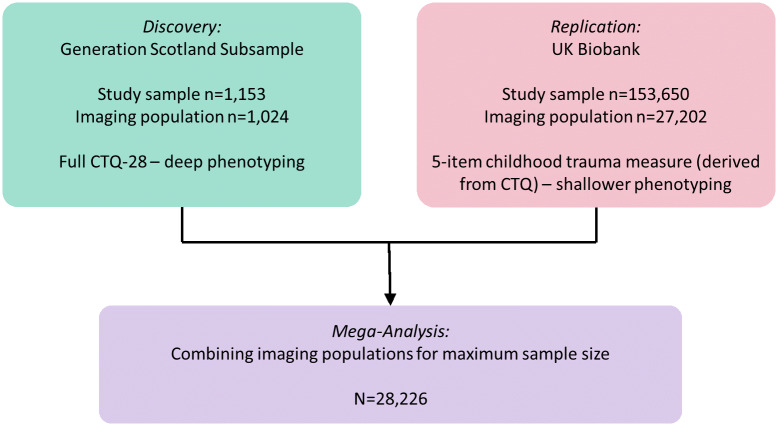
Analysis structure of this study, showing the initial discovery analysis in GS due to deeper phenotyping of childhood trauma, with replication in UKB and mega-analysis for maximum sample size.

The analysis comprised two independent populations: a subsample from The Generation Scotland (*n* = 1,024) and the UKB cohort (*n* = 27,202), totaling a population of *n* = 28,226 Figure 1. In both GS and UKB, *FreeSurfer* derived measures of cortical metrics and subcortical volumes were analyzed. The analysis focused initially on global cortical measures, lobar regions, and finally individual regional measures.

### The generation Scotland cohort

Generation Scotland: Scottish Family Health Study (GS hereafter) is a population-based cohort of over 24,000 individuals with in-depth phenotyping recruited between 2006 and 2011. A subsample of participants was recontacted in 2015–2019 for neuroimaging and further data collection. This subsample was used as the basis for this study and is described in detail elsewhere [43–46]. Cognitive assessments, blood sampling, physical measurements, and clinical questionnaires were also collected from GS participants, including the 28-item CTQ [5]. The subsample included *n* = 1,153 participants with CTQ and depression phenotyping, and *n* = 1,024 also had magnetic resonance imaging (MRI) data.

The 28-item CTQ is a questionnaire validated for self-report of abuse and neglect during childhood [5]. The questionnaire is made up of five subscales—Emotional Abuse (EA), Emotional Neglect (EN), Physical Abuse (PA), Physical Neglect (PN), and Sexual Abuse (SA)—with five items for each subscale, scored on a 5-point Likert scale rating frequency of each experience [1–5]. Emotional and PN items are reverse-scored. The minimization and denial scale makes up the remaining three items, a scale devised to help detect the under-reporting of CT [5]. The analyses reported here focused mainly on the summed score of the 25 subscale items of this questionnaire, scored on a scale of 25–125, which provides an index of the cumulative traumatic experience of an individual, consistent with previous literature (e.g. [47, 48]). The questionnaire can also be used to break scores down into severity categories of none–low, low–moderate, moderate–severe, and severe–extreme, for both total CTQ score and each subscale [49, 50].

All analyses were performed using scaled scores for the five CT subscales captured by the CTQ (and, in UKB, CTM) measures—EN, EA, PN, PA, and SA—as well as scaled composite “abuse” and “neglect” scores created by summing scores on the abuse and neglect items, respectively. Trauma subscales are often highly correlated with each other, and with summed total scores. Correlation matrices for the GS CTQ-28 and the UKB CTM subscales and total scores are presented in Supplementary Figure S1.

MDD diagnoses were derived from the SCID [43, 51] taken at GS baseline recruitment; a binary variable describing presence/absence of a lifetime diagnosis encompasses single-episode, chronic, postpartum onset, and depression with manic/hypomanic features. Two individuals with manic/hypomanic episodes alone were excluded from further analyses. The Quick Inventory of Depressive Symptomatology [52] was used as a measure of continuous (and current) depressive symptom severity at the time of the imaging clinic visit.

### MRI in GS

Imaging was conducted at two sites: Aberdeen and Dundee. In Aberdeen, brain MRI data were acquired on a 3-T Philips Achieva TX-series MRI system (Philips Healthcare, Best, Netherlands) with a 32-channel phased-array head coil with a back-facing mirror (software version 5.1.7; gradients with maximum amplitude 80 mT/m and maximum slew rate 100 T/m/s). In Dundee, images were acquired on a Siemens 3-T Prisma-FIT (Siemens Healthineers, Erlangen, Germany) with 20-channel head and neck coil and a back-facing mirror (software version VE11, gradient with max amplitude 80 mT/m and maximum slew rate 200 T/m/s). This study uses T1 structural data, although other sequences were also acquired [53, 54]. Structural measures were derived in-house from raw images using FreeSurfer version 5.3 [55–57]. The 1,070 images were segmented into GM, white matter, and cerebrospinal fluid. GM was further segmented into cortical and subcortical GM. The cortex was divided into 34 regions per hemisphere according to the Desikan–Killany atlas [58]. Visual quality control was undertaken for the exclusion of participants with major output errors in segmentation or parcellation. There were 424 subjects for whom a degree of manual editing was required to make small corrections to the images. Participants (*n* = 12) were excluded if errors could not be corrected in this way. For the 68 bilateral cortical regions, measures of mean thickness, surface area, and volume were calculated. For eight bilateral subcortical GM structures, volume alone was calculated. Coordinates of head position within the scanner were included as covariates.

### The UK Biobank cohort

UKB is a large, UK-based population cohort of over *n* = 500,000 adults recruited between 2006 and 2010 [59]. Data were collected over several instances of postal questionnaires and clinic visits. MRI was performed on *n* = 100,000, which was acquired in-clinic during the second assessment instance [59, 60]; at the time of writing, MRI data were available for *n* = 41,985 [61]. An online follow-up questionnaire was disseminated to participants after completion of the imaging appointment, comprising the Mental Health Questionnaire (MHQ; which included CT questions).

Five items relating to CT were included in the MHQ, derived from the 28-item CTQ with each item scored on a 5-point Likert scale (0–4). Hence, only one item related to each of the five subscales (as described in the fuller 28-item CTQ) was included in this cohort, making these data less sensitive than the GS and necessitating a focus throughout the study on total scores (with subscale results presented in Supplementary Material). For the purposes of this study, CT items were used on a 0–20 scale of the summed total of responses to these items. Any individuals with incomplete responses to the CT items were excluded from analyses, leaving a maximum sample of *n* = 153,650 for this report, of which *n* = 27,202 had MRI data available.

MDD diagnoses were derived from the International Classification of Diseases, Tenth Edition (ICD-10) diagnostic criteria assessed at the initial assessment center visit [62], and Composite International Diagnostic Interview Short-Form (CIDI-SF) [63] derived questionnaire in the MHQ at online follow-up. A modified form of the Generalized Anxiety Disorder Assessment (GAD-7; [64]) was delivered in the MHQ, as well, to indicate recent anxiety symptoms. The 4-item Patient Health Questionnaire (PHQ-4 [62]) was used to take the current mood level at the assessment center appointment.

### MRI scanning in UKB

Imaging for the 40,000 data release in January 2020 was conducted at three sites: Stockport, Newcastle, and Reading. These sites are equipped with identical MRI scanners—3 T Siemens Skyra (software VD13), using the standard Siemens 32-channel head coil [65]. This study uses T1 structural scans, although other sequences were also collected. Structural measures were derived for the 40,000 data release using FreeSurfer software [61]. See Supplementary Material, p.3.

### Statistical analyses

All statistical analyses were performed in R v.3.6.4. All analyses were corrected for multiple comparisons using the false-discovery rate (FDR) method.

Linear and binomial regressions were used to describe phenotypic relationships between scaled CT and demographic information, as well as features of depressive symptomatology in both cohorts. Numbers for each model varied in both cohorts, as different measures had different numbers of non-completers. These models adjusted for age and sex as minimum covariates.

To determine the relationships between scaled CT total and subscale scores and structural brain imaging features, linear regression models were conducted using the NLME package in R. For cortical structures, volume, surface area, and thickness measures were available; for subcortical structures volume data were available. Covariates included sex, age, age squared (to account for nonlinear effects of age), scaled intracranial volume, hemisphere, and scan site. For five summed lobar measures (lobar cortical volume, lobar cortical surface area, and mean lobar cortical thickness), the same covariates were used. For global measures (such as whole brain volume [WBV], total GM volume, and total white matter volume), the hemisphere was not a required covariate and Intracranial volume (ICV) was also excluded as a covariate due to high collinearity with the measures.

Imaging analyses were performed in *n* = 1,024 in GS, and *n* = 27,202 (*n* = 26,639 for WBV) in UKB. These numbers represent those with complete imaging data as well as complete CT data.

### Mega-analysis of imaging samples

For the mega-analysis, the total and subscale CT scores were scaled within each cohort, and then re-scaled across the merged mega-analysis sample. Structural imaging data including ICV were scaled within cohort. Analyses were conducted, as before, using the same covariates (Figure 1).

## Results

### Demographics characteristics

#### Generation Scotland

The GS study population (*n* = 1,153) was 58.54% female, with a mean age of 59.52 years (26–84 years). At the SCID interview 30.79% met the criteria for lifetime depression (Table 1).

**Table 1.** Demographic characteristics and psychiatric traits of the GS study population, incorporating participants with complete CTQ responses and SCID interviews.

			Prevalence
Trait	Total *N*		%
Sex (female)	1,153		*58.54*
Lifetime MDD	1,153		*30.79*
Recurrent MDD (≥2 episodes)^a^	355		*62.54*
Any CTQ subscale > “low”	1,153		*51.00*
Total CTQ score > “low”	1,153		*26.02*
Ever smoker	938		*43.71*

		Mean	Range
Age	1,153	59.52	*26–84*
Body Mass Index (BMI) (kg/m^2^)	1,153	28.21	*16.42–64.24*
Age of depression onset (yrs)^a^	355	33.45	*5–65*

			Prevalence
Subscales			*%*
Emotional abuse score > “low”	1,153		*21.25*
Emotional neglect score > “low”	1,153		*31.92*
Physical abuse score > “low”	1,153		*13.54*
Physical neglect score > “low”	1,153		*21.77*
Sexual abuse score > “low”	1,153		*13.80*

*Note:* Prevalence of the five CTQ subscales is also shown. Italic values represent the prevalence of discrete traits, or the range of continuous traits observed, in the total *N* quoted.

Abbreviations: CTQ, childhood trauma questionnaire; MDD, major depressive disorder.

a For the *n* = 355 reporting lifetime depression.

Using the CTQ score cutoffs (see Supplementary Materials, p.1), 26.02% of the study population reported experiencing “low–moderate” levels of CT, or above. Using the subscale-specific cutoffs, 51% of the study population reported a score of “low–moderate” or above on at least one CTQ subscale. The most frequent subtype of trauma reported was EN, and the least frequent were physical and SA (Table 1).

Total CTQ scores were significantly higher in females (mean = 34.75) than males (mean = 32.94; *t*_(df = 1,138.8)_ = 2.79, *p* = 0.0054), and were associated with higher BMI (β = 0.97, SE = 0.17, *p*_(FDR)_ = 2.31 x 10^−8^), but were not significantly associated with age (β = −4.04x10^−4^, SE = 0.0029, *p*_(FDR)_ = 0.89).

Participants with lifetime MDD diagnoses reported higher total CTQ scores (mean = 39.59) than healthy controls (mean = 31.52; *t*_(df = 442.54)_ = −9.11, *p* < 0.001). A significant positive association was found between total CTQ score and risk of lifetime depression (β = 0.68, OR = 1.98, *p*_(FDR)_ = 6.45x10^−18^). Depression risk was positively associated with total scores on all trauma subscales (Appendix 1). Higher total CTQ scores also predicted younger age-of-onset for depression (β = −3.2, 95% CI = –4.22 to −2.17, *p*_(FDR)_ = 1.44 x 10^−8^), and higher odds for a recurrent course of depressive illness (β = 0.36, OR = 1.43, *p*_(FDR)_ = 0.0022).

#### UK Biobank

The UKB study population (*n* = 153,650) was 56.31% female, with a mean age of 55.91 years (38–72 years). Of *n* = 94,379 participants for whom ICD-10 coded diagnostic information was available, 2.68% reported lifetime depression. For the *n* = 123,355 participants who completed a CIDI diagnostic questionnaire, 29.47% met the criteria for lifetime depression (Table 2).

**Table 2.** Demographic characteristics and psychiatric traits of the UKB study population, incorporating participants with complete childhood trauma responses.

		Prevalence
Trait	Total *N*	%
Sex (female)	153,650	*56.31*
Lifetime MDD International Classification of Diseases (ICD-10)	94,379	*2.68*
Lifetime MDD Composite International Diagnostic Interview (CIDI)	123,355	*29.47*
Recurrent MDD (≥2 episodes)^a^	23,004	*69.4*
Any CTQ subscale > “rarely true”	153,650	*59.28*
Ever smoker	153,306	*59.68*

		Mean	Range
Age	153,650	55.91	*38–72*
Total CTM score	153,650	1.76	*0–20*
BMI (kg/m^2^)	153,269	26.77	*12.12–67.38*
Age of depression onset (yrs)^b^	79,288	37.24	*2–78*

		Prevalence
Subscales		%
Emotional abuse item < “v. often true”	153,650	*14.41*
Emotional neglect item > “rarely true”	153,650	*47.59*
Physical abuse item > “rarely true”	153,650	*16.22*
Physical neglect item < “v. often true”	153,650	*16.21*
Sexual abuse item > “rarely true”	153,650	*8.76*

Abbreviations: CTM, childhood trauma metric; CTQ, childhood trauma questionnaire; MDD, major depressive disorder.

a Data come from the initial assessment center visit, not tied to a diagnostic questionnaire.

b Data come from the online follow-up to the imaging appointment, not tied to a diagnostic questionnaire.

Due to the less detailed nature of the childhood trauma metric (CTM) items in UKB’s MHQ, the severity cutoffs inherent to the CTQ could not be replicated. The proportion of participants responding higher than “Rarely True” (or lower than “Very Often True” for reverse-coded items) to any CTM item was 59.28% (Table 2).

Total CTM scores in UKB were significantly higher in females (mean = 1.85) than in males (mean = 1.64; *t*_(df = 152,796)_ = 16.85, *p* < 0.001). They showed a positive association with BMI (β = 0.37, SE = 0.012, *p*_(FDR)_ = 1.86 x 10^−220^), and a small but significant negative association with age (β = −0.0059, SE = 3.3x10^−4^, *p*_(FDR)_ = 3.28 x 10^−72^).

Participants with CIDI-coded lifetime MDD had higher CTM scores (mean = 2.57) than healthy controls (mean = 1.32; *t*_(df = 48,247)_ = −72.35, *p* < 0.001); similarly, those with ICD-10-coded lifetime MDD had higher CTM scores (mean = 3.34) than healthy controls (mean = 1.67; t_(df = 2582)_ = −22.18, *p* < 0.001). Significant positive associations were found between total CTM scores and risk of lifetime depression for CIDI (β = 0.49, OR = 1.63, *p*_(FDR)_ < 1 x 10^−314^), and ICD-10 data (β = 0.4, OR = 1.49, *p*_(FDR)_ = 8.52 x 10^−186^). ICD-10- and CIDI-diagnosed depression risk both positively associated with responses to each of the CTM subscale items, in addition (Appendix 1). Total CTM scores predicted younger age of depression onset (β = −2.1, SE = 0.046, *p*_(FDR)_ < 1 x 10^−314^), and higher odds of a recurrent course of depressive illness (β = 0.22, OR = 1.24, *p*_(FDR)_ = 2.66 x 10^−51^).

### Neuroimaging findings

We took the approach of analyzing relationships between scaled CT scores and brain metrics in increasing levels of detail, starting with global brain and gray/white matter volumes, then lobar-level metrics, then across all regions.

#### Global brain measures

The relationships between CT scores and global brain metrics were analyzed in GS (*n* = 1,024), UKB (*n* = 26,639), and the mega-analysis sample (*n* = 28,226).

In GS, significant negative associations were revealed between total CTQ score and global white matter volume (β = −0.0852, SE = 0.0258, *p*_(FDR)_ = 0.0015), global GM volume (β = −0.0682, SE = 0.0219, *p*_(FDR)_ = 0.0019), and whole brain volume (β = −0.0844, SE = 0.0239, *p*_(FDR)_ = 0.0013; Appendix 2).

In UKB, CTM scores showed significant negative associations with global white matter volume (β = −0.0311, SE = 0.0049, *p*_(FDR)_ = 2.67x10^−10^), global GM volume (β = −0.0433, SE = 0.0049, *p*_(FDR)_ = 5.27x10^−18^), and whole brain volume (β = −0.0399, SE = 0.0048, *p*_(FDR)_ = 1.75x10^−16^; Appendix 2).

In the mega-analysis population, the whole-brain analyses followed much the same pattern, with significant negative associations between CT scores and global white matter volume (β = −0.0330, SE = 0.0048, *p*_(FDR)_ = 9.15x10^−12^), global GM volume (β = −0.0442, SE = 0.0048, *p*_(FDR)_ = 1.74x10^−19^), and whole brain volume (β = −0.0417, SE = 0.0047, *p*_(FDR)_ = 1.51 x 10^−18^; Appendix 2).

#### Lobar measures

The relationships between scaled CT scores and the whole-lobe cortical volume, whole-lobe cortical surface area, and mean cortical thickness of all five lobar regions were examined and analyzed in GS (*n* = 1,024), UKB (*n* = 27,202), and the mega-analysis sample (*n* = 28,226).

In GS, there were significant negative associations between total CTQ scores and both cortical surface areas and cortical volumes of the frontal, parietal, temporal, occipital, and cingulate lobes (Appendix 2). There were no significant associations for lobar cortical thicknesses.

In UKB, we observed significant negative associations between total CTM score and cortical volumes and cortical surface areas of all five lobes, but no associations with cortical thicknesses were observed (Appendix 2).

In the mega-analysis sample, relationships between CT score and cortical volume and surface areas of all five lobes were significant (Appendix 2). Again, no associations between CT scores and whole-lobe cortical thickness were observed.

#### Regional measures

Regional analyses examined the relationships between CT scores and volumes of 34 cortical and 8 subcortical regions, as well as thickness and surface area for the same 34 cortical regions in GS (*n* = 1,024). In UKB (*n* = 27,202) and mega-analysis (*n* = 28,226), 33 cortical regions were examined on the same metrics, plus 8 subcortical regional volumes.

**Figure 2.**
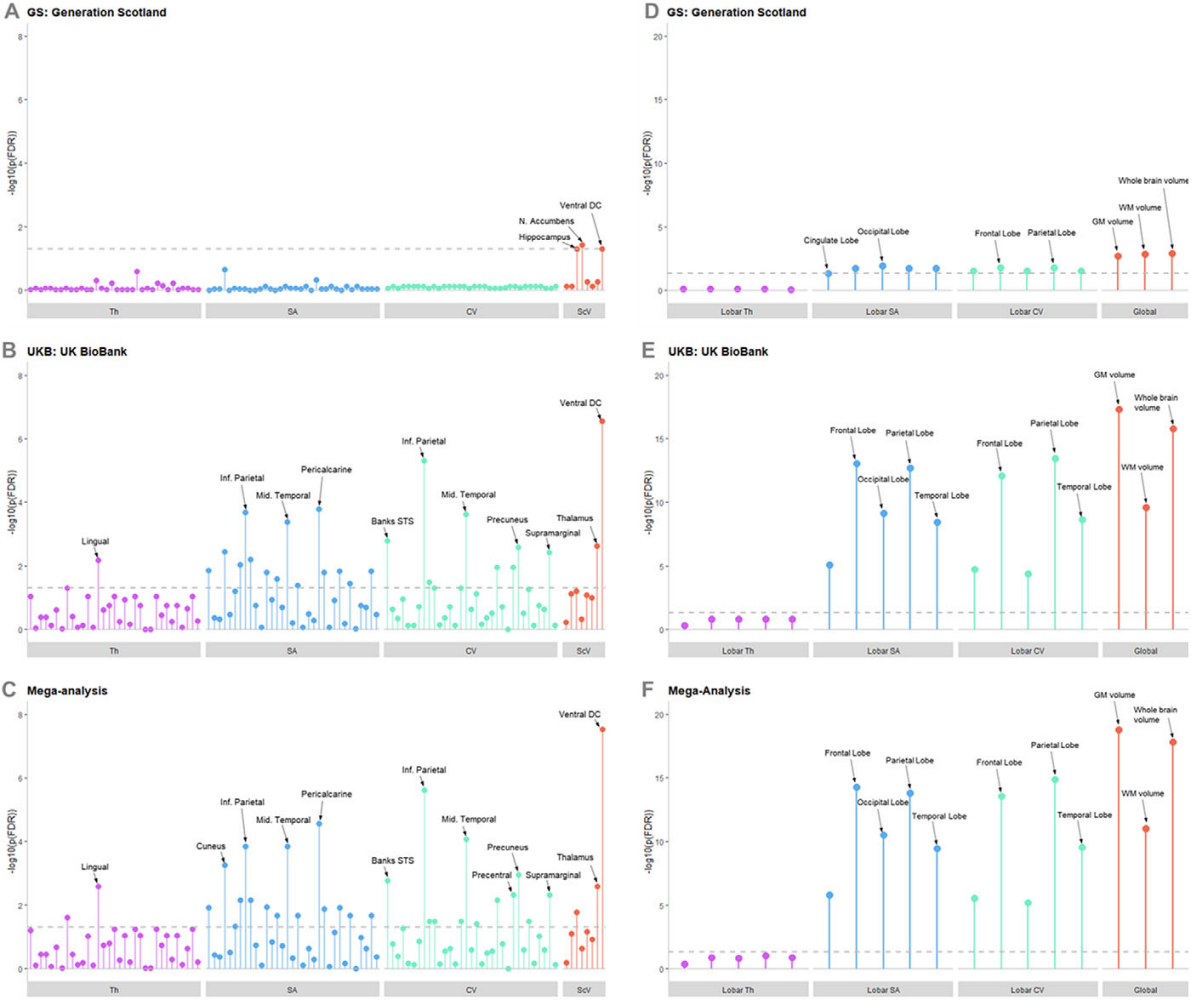
Lollipop plots showing –log10 of *p*_(FDR)_ for each region and metric in the regional analysis for (A) GS, (B) UKB, and (C) the mega-analysis; and in the global and lobar analyses for (D) GS, (E) UKB, and (F) the meg-analysis. The –log10 of *p*_(FDR)_ = 0.05 is represented by the dotted gray line, any points exceeding this line achieved statistical significance after FDR correction. Regions of higher significance are labeled; the order in which other regions are presented can be found in Supplementary Material. Abbreviations: CV, cortical volume; SA, cortical surface area; ScV, subcortical volume; Th, cortical thickness.

In GS, across all regional metrics analyzed three regions were significantly associated after FDR correction (Figure 2, Appendix 1). These were the volumes of the hippocampus (β = −0.0582, SE = 0.0247, *p*_(FDR)_ = 0.050), nucleus accumbens (β = −0.0654, SE = 0.0231, *p*_(FDR)_ = 0.037), and ventral diencephalon (VDc; Supplementary Figure S2; β = −0.0526, SE = 0.0218, *p*_(FDR)_ = 0.050). All three regions are subcortical areas, and all were negatively associated with total CTQ score, with a higher CTQ score predicting a lower volume of these regions.

In UKB, many significant associations were found (Figure 2; Appendix 1), due to the higher power afforded by the larger sample size in this data set. Of note was the replication of a significant negative association between the total CTM score and the volume of the VDc (β = −0.0220, SE = 0.0040, *p*_(FDR)_ = 2.84x10^−7^). Significant associations between CTM scores and cortical regions were demonstrated—again showing more consistent associations with SA and volume than cortical thickness. Regions with the strongest effect sizes were the volume of the inferior parietal lobule (β = −0.0223, SE = 0.0042, *p*_(FDR)_ = 4.78 x 10^−6^), and the surface area of the pericalcarine cortex (β = −0.0248, SE = 0.0054, *p*_(FDR)_ = 1.61 x 10^−4^).

**Figure 3.**
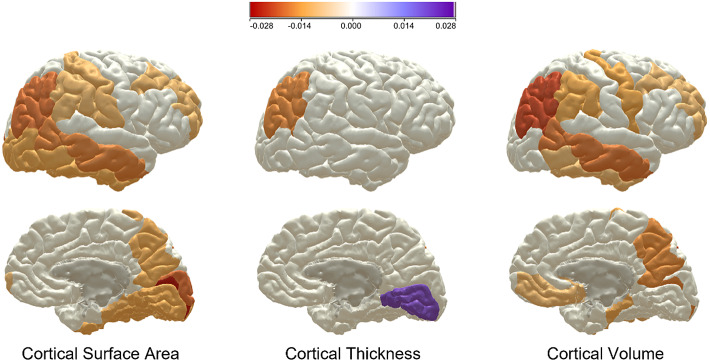
Map of showing Beta values for regions significantly associated with childhood trauma in the mega-analysis, for cortical thickness, cortical surface area, and cortical volume. The red-toned colors represent a negative association and the purple-toned represent a positive association, with the strength of that association denoted by the shade of the color.

All findings from the UKB analyses were replicated in the mega-analysis, which also revealed some novel significant associations (Figures 2, 3, Appendix 2). Two of the three findings in the GS regional analysis were also replicated in the mega-analysis; the significant negative associations in the hippocampus (β = −0.0119, SE = 0.0044, *p*_(FDR)_ = 0.017), and the VDc (β = −0.0232, SE = 0.0039, *p*_(FDR)_ = 2.91x10^−8^). The volume of the thalamus was additionally significant at mega-analysis (β = −0.0134, SE = 0.0039, *p*_(FDR)_ = 0.0026).

#### Childhood trauma subscales

Across all three global brain metrics in GS, significant associations were demonstrated with EA, PA, SA, and Abuse composite score. This was replicated in the UKB and mega-analysis. In GS, however, no significant association was indicated for EN, PN, or Neglect Composite score. This was not replicated in the UKB and mega-analysis; EN, PN, and Neglect composite were significantly associated with all three global brain metrics in these analyses (Appendix 3).

Similarly, at the lobar level, imaging samples demonstrated associations with some subscale scores with particularly consistent results between PA, SA, and abuse composite score and lobar surface areas; and between PA and abuse composite score and lobar volumes. Very few significant associations were found with lobar thicknesses, excepting significant associations with both abuse and neglect composite scores in the UKB analysis only (Appendix 3). At the regional level in GS, the only significant associations observed were between the PA score and the surface area of superior parietal cortex (β = −0.0771, SE = 0.0235, *p*_(FDR)_ = 0.036), the cortical volume of the entorhinal cortex (β = −0.0733, SE = 0.0224, *p*_(FDR)_ = 0.037), and 6 of the 8 subcortical volumes: the hippocampus, VDc, thalamus, amygdala, nucleus accumbens, and pallidum (Appendix 3). No other subscale or composite score showed any significant associations with any regional metric in GS, except for the neglect composite score which associated significantly with the volume of the nucleus accumbens (β = −0.0805, SE = 0.0229, *p*_(FDR)_ = 0.0037).

In UKB and in the mega-analysis, many associations were found between the different trauma subtypes and regional imaging metrics, including some associations between the EA items and cortical thickness measures (Appendix 3). The abuse composite score demonstrated significant associations with the volumes of the hippocampus, thalamus, and VDc in both the UKB analysis and mega-analysis—the same three subcortical regions with which total CT scores associated in the mega-analysis (Appendix 3). The only region for which a significant association was found across all three analyses was the VDc (GS: β = −0.0676, SE = 0.0217, *p*_(FDR)_ = 0.005; UKB: β = −0.0212, SE = 0.004, *p*_(FDR)_ = 1.01x10^−6^; Mega-analysis: β = −0.023, SE = 0.0039, *p*_(FDR)_ = 4.19x10^−8^).

## Discussion

This study reports psychiatric and structural brain correlates of CT in a mega-analysis of two independent UK cohorts. The mega-analysis sample size of *n* = 28,226 improves significantly upon the sample sizes of similar multicenter studies [41, 42].

The data presented here provide further validation for proposed relationships between CT and depressive pathology in adulthood. These links to poor mental health outcomes have been demonstrated previously [66], as have links to poor physical health outcomes [4]. The study also highlights the high prevalence of traumatic childhood experiences in our population, with 51 and 59% of participants reporting having experienced some kind of ACE in GS and UKB, respectively. In the GS CTQ data, where more detailed analysis was possible, we observed what might be considered a “clinically significant” level of trauma (a total CTQ score over the “low–moderate” threshold) in 26% of the study population. This fits with previous estimates of CT prevalence [1, 2, 4]. The association between higher CT score and younger age of onset for MDD is strong in both cohorts (GS: β = −3.2, 95% CI = –4.22 to −2.17, *p*_(FDR)_ = 1.44 x 10^−8^; UKB: β = −2.1, SE = 0.046, *p*_(FDR)_ < 1 x 10^−314^). This replication indicates that CT is having early effects on the development of psychopathology, and given the links between age of onset and poorer long-term outcomes in MDD could also suggest a link with lifetime severity [67].

The severity of traumatic childhood experiences associated with global brain volume, global gray and white matter volumes, and the volumes and surface areas of all five major brain lobes at mega-analysis with effect sizes ranging from −0.0442 to −0.0204 (Appendix 2). These results point to a whole-brain effect of adverse childhood events on brain development, perhaps with exacerbated results in particular regions. Interestingly, in the global, lobar, and regional analyses very few associations are seen between CT scores and cortical thickness metrics. The evidence of this work suggests that CT severity may be more strongly associated with cortical surface areas and volumes, especially in front and temporo-parietal regions (Figure 3). Specific reductions in cortical surface area rather than thickness have also been reported in the context of adolescent depression [68], this could reflect a common feature associated with early onset of psychiatric morbidity. Cortical surface area has been shown to be established earlier in development within a shorter window than cortical thickness [69]; adverse experiences within this earlier window could be interrupting the developmental process resulting in the surface area findings presented here. Cortical thickness is established over a much longer time period, well into adulthood [69], and may therefore be less susceptible to interruption by early life events.

The effect sizes found in the global and lobar analyses presented here are larger than those found in the regional analyses, but there were nevertheless multiple significant associations demonstrated. Total CT scores were significantly associated with multiple cortical and subcortical regional volumes at mega-analysis, with effect sizes ranging from −0.0262 to 0.0208. The strongest effect sizes were seen in the negative relationship between total CT scores and the volume of the VDc (β = −0.0232, SE = 0.0039, *p*_(FDR)_ = 2.91 x10^−8^), the volume of the inferior parietal cortex (β = −0.0225, SE = 0.0042, *p*_(FDR)_ = 2.43 x 10^−6^), and the surface area of the pericalcarine cortex (β = −0.0262, SE = 0.0053, *p*_(FDR)_ = 2.75 x 10^−5^); as well as the positive association with the thickness of the lingual cortex (β = 0.0208, SE = 0.0053, *p*_(FDR)_ = 0.0026). The subcortical regions the hippocampus (β = −0.0119, SE = 0.0044, *p*_(FDR)_ = 0.017) and the thalamus (β = −0.0134, SE = 0.0039, *p*_(FDR)_ = 0.0026) were also significantly associated with total CT scores at mega-analysis, and in the GS analysis the nucleus accumbens was significant with a relatively strong effect size (β = −0.0654, SE = 0.023, *p*_(FDR)_ = 0.037). We have previously reported an association between elevated hair glucocorticoids and the volume of the nucleus accumbens (as well as with MDD and CT) in the GS imaging sample, suggesting a possible link to the HPA axis and stress reactivity [70]. Indeed, all of the subcortical regions significantly associated with CT scores in this analysis display possible links to the stress hormone system.

The VDc was the only regional metric in which a consistent effect was seen across both GS and UKB, as well as in the mega-analysis. The VDc is a *FreeSurfer-*derived region comprising several structures, including the hypothalamus—a region crucial to the regulation of the stress response—as well as subthalamic nuclei, the substantia nigra, the medial and lateral geniculate nuclei and associated white matter structures (Figure 2; as described [71, 72]). Lower VDc (and nucleus accumbens) volumes have also been reported in patients with earlier onset of MDD REF ANCELIN, indicating possible neurostructural links between the incidence of CT and the early onset of depressive symptoms. A significant relationship was also observed between CT and the volume of the thalamus (β = −0.0134, SE = 0.0039, *p*_(FDR)_ = 0.0026). This could implicate involvement of thalamic/hypothalamic function after CT, supporting previous work [31–37]. Future work could employ finer-grained approaches to probe associations with this region more deeply.

It is well established that different types of trauma may affect the brain in different ways [10]. The subscale mega-analysis presented here generally found an influence of PA on a greater number of regional surface areas and cortical volumes than other subscales, although PN is also associated with a high number of regional surface areas (Appendix 3). Interestingly, PA and PN showed a moderate-to-strong correlation (see Supplementary Figure S1) with total CTQ score in GS (R = 0.691, 0.694, respectively) and in UKB (R = 0.661, 0.571, respectively), but EA and neglect items were more strongly correlated with total scores. This suggests that the findings for PA and neglect subscales may therefore represent specific effects, not merely due to strong correlations with total CT scores. EA was the only subscale that had any associations with regional cortical thicknesses (Appendix 3). Subcortical regional volumes were affected by all subscales of CT, with the abuse composite measure showing the same pattern of effects as the total CT score—negatively associating with the hippocampus (β = −0.0124, SE = 0.044, *p*_(FDR)_ = 0.013), thalamus (β = −0.0166, SE = 0.0040, *p*_(FDR)_ = 1.03x10^−4^), and VDc (β = −0.0249, SE = 0.0040, *p*_(FDR)_ = 2.42x10^−9^)—indicating that abuse may be more strongly associated with structural brain changes after CT than neglect. It is important, however, to treat the UKB and mega-analysis subscales with caution due to the single-factor nature of the subscale items in UKB. In GS alone, due to the lower power of the cohort in comparison, few significant associations were found in the subscale analyses. Notably, PA is associated significantly with the surface area of the superior parietal cortex (β = −0.0771, SE = 0.024, *p*_(FDR)_ = 0.036), and the volume of the entorhinal cortex (β = −0.0733, SE = 0.022, *p*_(FDR)_ = 0.037), as well as 6 of 8 subcortical regions (Appendix 3). No other subscale was associated with any cortical measures in GS, although physical and EN both associated with the volume of the nucleus accumbens (β = −0.0681, SE = 0.023, *p*_(FDR)_ = 0.025; β = −0.0743, SE = 0.023, *p*_(FDR)_ = 0.0099, respectively), for which the neglect composite score was accordingly significant (β = −0.0805, SE = 0.023, *p*_(FDR)_ = 0.0037). In the global and lobar level analyses in GS, physical and SA was associated with most lobar surface areas, and PA associated with most lobar volumes (Appendix 3). The physical, emotional, and SA items and the abuse composite score are each associated with the global brain volume, and global gray and white matter measures (effect sizes ranging from −0.114 - -0480; Appendix 3); meanwhile, neither neglect item not the composite score associated with any global measure in GS alone. This suggests that the effect of CT on global brain development may be more influenced by experiences of abuse than neglect, with PA seeming to display the strongest influence.

### Limitations

Despite the very strong sample size derived from mega-analysis of two independent UK cohorts, enabling well-powered brain-wide analysis and conservative correction for multiple testing, the multi-sample nature of this study introduces unavoidable limitations. One limitation is the use of different CT measures between the cohorts. The GS subsample employed the full CTQ-28, while the UKB study used a five-item CT metric comprising one item taken from each subscale of the CTQ-28. Although this does mean that the two measures share features, they are not precisely equivalent. The cruder nature of the UKB measure is evident in the high report of EN in UKB (47.59% responding “rarely true” or above, compared to 31.92% reporting a “low–moderate” score or above in GS). The single item used to denote EN in UKB was “When I was growing up… I felt loved,” scored “never true” to “very often true.” This item carries a high emotional valence, which may have led respondents to over-report compared to the scores derived from a multi-factorial scale in the full CTQ-28. The subscale analyses presented here should be treated particularly cautiously as a result, in both UKB and in the mega-analysis sample due to the heavy representation of UKB participants there. This mismatch in sample sizes is a further limitation to the mega-analysis presented here—the UKB cohort makes up the majority of the mega-analysis sample and the results from the mega-analysis are, therefore, likely to be disproportionately influenced by the UKB sample compared to the GS sample. Some other metrics used in the two cohorts—such as psychiatric diagnostic tools—differed, leading again to a degree of mismatch in the data derived from each cohort. The imaging protocols, too, were conducted at different sites and scanners and according to different protocols, although the “scan site” variable employed in all imaging analyses attempts to account for this, and both cohorts used the same FreeSurfer software to derive structural data from MRI scans. Despite these limitations, the two samples were relatively demographically homogenous compared to previous multi-cohort studies, as can be seen by comparing the study population demographics presented for each cohort (Tables 1, 2). Furthermore, the two cohorts complement each other—the GS sample utilizes the full CTQ-28, therefore employing a more comprehensive measure of the CT phenotype than UKB, which uses a shallower CT screen, but—with its impressive sample size—overcomes the slightly underpowered GS sample.

Further limitations are evident in the nature of the CTQ in itself—retrospective report of traumatic childhood experiences has been shown to identify a different population than prospective report [73]. Although it is difficult to conceive of a study on this scale that does not rely on retrospective report, it is important to consider these results in light of this fact. Neither cohort provides any information on the timing of reported trauma, either. This has been shown to affect outcomes, with different types of trauma affecting brain development in different ways according to the timing of these events [74]. An additional concern relevant to any population imaging cohort is evidence that “healthy bias” may exist in the group of participants who were able and willing to attend MRI scanning appointments as compared to the un-scanned members of the cohort. Healthy bias has been demonstrated in UKB [75] but may well be present in GS, thus participants with more extreme symptoms following CT may not have been included in the study populations reported here.

Finally, although the use of metrics from broad brain regions derived by FreeSurfer processing of the MRI scans was practical for an analysis of this scale, they do not allow for the more detailed analysis of subregions that may play different functional roles. More finely-grained analysis techniques such as VBM could be used in future work to enhance understanding of the functional significance of the findings presented here. It is also worth noting that the effect sizes reported here are uniformly low (β < 0.1), indicating that despite the statistical significance of the reported findings the magnitude of the difference in regional and global volumes associated with CT scores is small.

### Conclusions

This large mega-analysis study reports associations between CT and altered structural metrics across the adult brain, with analyses performed brain-wide in a non-hypothesis driven manner. These effects can be seen at the global, lobar, and regional levels. A negative association between CTM scores and the VDc—an area comprising the hypothalamus and other thalamus-associated regions—replicated across all three analyses. The effects on global brain measures are particularly noteworthy, and support evidence of an involvement of traumatic childhood experiences in global brain development.

## Data Availability

According to the terms of consent for Generation Scotland participants, access to data must be reviewed by the Generation Scotland Access Committee. Applications should be made to access@generationscotland.org.

## Abbreviations

ACEs: adverse childhood experiences
CA: childhood adversity
CT: childhood trauma
CTM: childhood trauma metric (shorthand for UKB childhood trauma items)
CTQ: childhood trauma questionnaire
EA: emotional abuse
EN: emotional neglect
PA: physical abuse
PN: physical neglect
SA: sexual abuse
SCID: structured clinical interview for DSM-IV
VDc: ventral diencephalon

## Supplementary material

For supplementary material accompanying this paper visit http://doi.org/10.1192/j.eurpsy.2022.2347. click here to view supplementary material
