# Supplementary material for: Structural brain correlates of childhood trauma with replication across two large, independent community-based samples
Source: Eur Psychiatry. 2023 Jan 26;66(1):e19. doi: 10.1192/j.eurpsy.2022.2347 (PMC9970154; doi:10.1192/j.eurpsy.2022.2347)
Supplement: Supplementary file 1 [file epasup.zip › S0924933822023471sup002.docx]

Appendix 1: Regression model results for associations between CT metric and depression diagnosis in the GS and UKB cohorts. Significant p-values after FDR correction are indicated in bold.

| **Binomial regression outputs for the association between CTQ scores and lifetime** | | | | | | | |
| --- | --- | --- | --- | --- | --- | --- | --- |
| **depression diagnosis in the GS cohort study population (n=1,153)** | | | | | | |  |
|  |  |  |  |  |  |  |  |
|  | term | Beta | std error | p value | Odds Ratio | p(FDR) |  |
| Total CTQ Score | (Intercept) | 0.595 | 0.521 | 0.2532 | 1.813 | **0.2532** |  |
|  | age | -0.037 | 0.007 | 6.01E-08 | 0.964 | **1.50E-07** |  |
|  | sexM | -0.761 | 0.146 | 1.83E-07 | 0.467 | **3.05E-07** |  |
|  | BMI | 0.035 | 0.012 | 0.0034 | 1.036 | **0.0042** |  |
|  | totalCTQ | 0.684 | 0.078 | 1.29E-18 | 1.981 | **6.45E-18** |  |
|  |  |  |  |  |  |  |  |
| Total Emotional Abuse Score | (Intercept) | 0.055 | 0.511 | 0.914 | 1.057 | **0.914** |  |
|  | age | -0.032 | 0.007 | 1.78E-06 | 0.969 | **4.46E-06** |  |
|  | sexM | -0.670 | 0.145 | 3.89E-06 | 0.512 | **6.49E-06** |  |
|  | BMI | 0.043 | 0.012 | 0.00022 | 1.044 | **0.00028** |  |
|  | TotalEA | 0.594 | 0.074 | 8.39E-16 | 1.811 | **4.20E-15** |  |
|  |  |  |  |  |  |  |  |
| Total Emotional Neglect Score | (Intercept) | 0.390 | 0.511 | 0.45 | 1.477 | 0.45 |  |
|  | age | -0.036 | 0.007 | 6.46E-08 | 0.965 | **1.08E-07** |  |
|  | sexM | -0.913 | 0.146 | 4.57E-10 | 0.401 | **1.14E-09** |  |
|  | BMI | 0.043 | 0.012 | 0.00026 | 1.044 | **0.00033** |  |
|  | TotalEN | 0.572 | 0.069 | 8.03E-17 | 1.771 | **4.02E-16** |  |
|  |  |  |  |  |  |  |  |
| Total Physical Abuse Score | (Intercept) | 0.355 | 0.500 | 0.48 | 1.426 | 0.48 |  |
|  | age | -0.036 | 0.007 | 4.33E-08 | 0.965 | **1.08E-07** |  |
|  | sexM | -0.845 | 0.143 | 3.05E-09 | 0.430 | **1.53E-08** |  |
|  | BMI | 0.044 | 0.011 | 0.00012 | 1.045 | **0.00015** |  |
|  | TotalPA | 0.351 | 0.068 | 2.18E-07 | 1.420 | **3.63E-07** |  |
|  |  |  |  |  |  |  |  |
| Total Physical Neglect Score | (Intercept) | 0.391 | 0.503 | 0.44 | 1.479 | 0.44 |  |
|  | age | -0.037 | 0.007 | 1.86E-08 | 0.964 | **4.65E-08** |  |
|  | sexM | -0.843 | 0.143 | 3.45E-09 | 0.430 | **1.72E-08** |  |
|  | BMI | 0.045 | 0.011 | 9.39E-05 | 1.046 | **0.00012** |  |
|  | TotalPN | 0.366 | 0.066 | 3.33E-08 | 1.442 | **5.55E-08** |  |
|  |  |  |  |  |  |  |  |
| Total Sexual Abuse Score | (Intercept) | 0.193 | 0.500 | 0.700254 | 1.212 | 0.70 |  |
|  | age | -0.032 | 0.006 | 6.46E-07 | 0.968 | **1.62E-06** |  |
|  | sexM | -0.685 | 0.143 | 1.73E-06 | 0.504 | **2.88E-06** |  |
|  | BMI | 0.041 | 0.012 | 0.00048 | 1.041 | **0.00060** |  |
|  | TotalSA | 0.417 | 0.076 | 4.04E-08 | 1.518 | **2.02E-07** |  |
|  |  |  |  |  |  |  |  |

| **Binomial regression outputs for the association between CT scores and lifetime** | | | | | | | |
| --- | --- | --- | --- | --- | --- | --- | --- |
| **ICD-10 depression diagnosis in the UKB cohort study population (n=94,379)** | | | | | | | |
|  |  |  |  |  |  |  |  |
|  | term | Beta | std error | p value | Odds Ratio | p(FDR) |  |
| Total CT Score | (Intercept) | -4.388 | 0.177 | 2.68E-136 | 0.012 | **6.70E-136** |  |
|  | Age | -0.017 | 0.003 | 1.01E-10 | 0.983 | **1.01E-10** |  |
|  | sexMale | -0.565 | 0.044 | 2.86E-37 | 0.568 | **3.58E-37** |  |
|  | bmi | 0.067 | 0.004 | 8.07E-79 | 1.069 | **1.34E-78** |  |
|  | totalCTQ | 0.396 | 0.014 | 1.70E-186 | 1.486 | **8.52E-186** |  |
|  |  |  |  |  |  |  |  |
| Emotional Abuse Iten Response | (Intercept) | -4.562 | 0.176 | 2.15E-147 | 0.010 | **1.08E-146** |  |
|  | Age | -0.015 | 0.003 | 6.17E-09 | 0.985 | **6.17E-09** |  |
|  | sexMale | -0.550 | 0.044 | 2.01E-35 | 0.577 | **2.52E-35** |  |
|  | bmi | 0.071 | 0.004 | 1.83E-89 | 1.073 | **3.06E-89** |  |
|  | EA | 0.342 | 0.014 | 1.81E-141 | 1.408 | **4.52E-141** |  |
|  |  |  |  |  |  |  |  |
| Emotional Neglect Item Response | (Intercept) | -4.307 | 0.175 | 4.83E-133 | 0.013 | **1.21E-132** |  |
|  | Age | -0.020 | 0.003 | 4.38E-15 | 0.980 | **4.38E-15** |  |
|  | sexMale | -0.627 | 0.044 | 8.22E-46 | 0.534 | **1.03E-45** |  |
|  | bmi | 0.072 | 0.004 | 5.07E-94 | 1.075 | **8.44E-94** |  |
|  | EN | 0.422 | 0.017 | 1.61E-136 | 1.524 | **8.06E-136** |  |
|  |  |  |  |  |  |  |  |
| Physical Abuse Item Response | (Intercept) | -4.389 | 0.175 | 2.08E-138 | 0.012 | **1.04E-137** |  |
|  | Age | -0.017 | 0.003 | 5.40E-11 | 0.983 | **5.40E-11** |  |
|  | sexMale | -0.640 | 0.044 | 1.12E-47 | 0.527 | **1.41E-47** |  |
|  | bmi | 0.071 | 0.004 | 1.66E-89 | 1.073 | **4.16E-89** |  |
|  | PA | 0.249 | 0.015 | 1.30E-62 | 1.282 | **2.16E-62** |  |
|  |  |  |  |  |  |  |  |
| Physical Neglect Item Response | (Intercept) | -4.137 | 0.174 | 1.45E-124 | 0.016 | **7.27E-124** |  |
|  | Age | -0.023 | 0.003 | 4.95E-19 | 0.977 | **4.95E-19** |  |
|  | sexMale | -0.608 | 0.044 | 2.28E-43 | 0.545 | **3.80E-43** |  |
|  | bmi | 0.074 | 0.003 | 9.47E-99 | 1.077 | **2.37E-98** |  |
|  | PN | 0.191 | 0.015 | 4.59E-38 | 1.211 | **5.74E-38** |  |
|  |  |  |  |  |  |  |  |
| Sexual Abuse Item Response | (Intercept) | -4.322 | 0.175 | 1.03E-134 | 0.013 | **5.15E-134** |  |
|  | Age | -0.019 | 0.003 | 7.48E-14 | 0.981 | **7.48E-14** |  |
|  | sexMale | -0.554 | 0.044 | 9.53E-36 | 0.575 | **1.19E-35** |  |
|  | bmi | 0.072 | 0.004 | 1.67E-93 | 1.075 | **4.18E-93** |  |
|  | SA | 0.204 | 0.013 | 3.62E-56 | 1.226 | **6.04E-56** |  |
|  |  |  |  |  |  |  |  |

| **Binomial regression outputs for the association between CT scores and lifetime** | | | | | | | |
| --- | --- | --- | --- | --- | --- | --- | --- |
| **CIDI depression diagnosis in the UKB cohort study population (n=123,355)** | | | | | | | |
|  |  |  |  |  |  |  |  |
|  | term | Beta | std error | p value | Odds Ratio | p(FDR) |  |
| Total CT Score | (Intercept) | 0.606 | 0.059 | 1.70E-24 | 1.832 | **1.70E-24** |  |
|  | Age | -0.041 | 0.001 | 0.00E+00 | 0.960 | **0.00E+00** |  |
|  | sexMale | -0.778 | 0.014 | 0.00E+00 | 0.460 | **0.00E+00** |  |
|  | bmi | 0.041 | 0.001 | 8.77E-181 | 1.042 | **1.10E-180** |  |
|  | totalCTQ | 0.492 | 0.007 | 0.00E+00 | 1.635 | **0.00E+00** |  |
|  |  |  |  |  |  |  |  |
| Emotional Abuse Item Response | (Intercept) | 0.365 | 0.059 | 6.11E-10 | 1.441 | **6.11E-10** |  |
|  | Age | -0.039 | 0.001 | 0.00E+00 | 0.962 | **0.00E+00** |  |
|  | sexMale | -0.749 | 0.014 | 0.00E+00 | 0.473 | **0.00E+00** |  |
|  | bmi | 0.045 | 0.001 | 5.13E-212 | 1.046 | **6.42E-212** |  |
|  | EA | 0.426 | 0.007 | 0.00E+00 | 1.531 | **0.00E+00** |  |
|  |  |  |  |  |  |  |  |
| Emotional Neglect Item Response | (Intercept) | 0.627 | 0.059 | 1.86E-26 | 1.873 | **1.86E-26** |  |
|  | Age | -0.044 | 0.001 | 0.00E+00 | 0.957 | **0.00E+00** |  |
|  | sexMale | -0.821 | 0.014 | 0.00E+00 | 0.440 | **0.00E+00** |  |
|  | bmi | 0.046 | 0.001 | 4.23E-226 | 1.047 | **5.29E-226** |  |
|  | EN | 0.439 | 0.006 | 0.00E+00 | 1.551 | **0.00E+00** |  |
|  |  |  |  |  |  |  |  |
| Physical Abuse Item Response | (Intercept) | 0.482 | 0.058 | 1.33E-16 | 1.619 | **1.33E-16** |  |
|  | Age | -0.040 | 0.001 | 0.00E+00 | 0.961 | **0.00E+00** |  |
|  | sexMale | -0.823 | 0.014 | 0.00E+00 | 0.439 | **0.00E+00** |  |
|  | bmi | 0.044 | 0.001 | 2.87E-208 | 1.045 | **3.58E-208** |  |
|  | PA | 0.289 | 0.006 | 0.00E+00 | 1.336 | **0.00E+00** |  |
|  |  |  |  |  |  |  |  |
| Physical Neglect Item Response | (Intercept) | 0.612 | 0.058 | 4.06E-26 | 1.844 | **4.06E-26** |  |
|  | Age | -0.044 | 0.001 | 0.00E+00 | 0.957 | **0.00E+00** |  |
|  | sexMale | -0.789 | 0.014 | 0.00E+00 | 0.454 | **0.00E+00** |  |
|  | bmi | 0.047 | 0.001 | 1.22E-248 | 1.048 | **2.04E-248** |  |
|  | PN | 0.122 | 0.006 | 8.49E-86 | 1.129 | **1.06E-85** |  |
|  |  |  |  |  |  |  |  |
| Sexual Abuse Item Response | (Intercept) | 0.520 | 0.058 | 3.53E-19 | 1.681 | **3.53E-19** |  |
|  | Age | -0.042 | 0.001 | 0.00E+00 | 0.959 | **0.00E+00** |  |
|  | sexMale | -0.750 | 0.014 | 0.00E+00 | 0.472 | **0.00E+00** |  |
|  | bmi | 0.046 | 0.001 | 5.03E-233 | 1.047 | **6.28E-233** |  |
|  | SA | 0.231 | 0.006 | 1.01E-290 | 1.260 | **1.69E-290** |  |
|  |  |  |  |  |  |  |  |
