# Supplementary material for: Structural brain correlates of childhood trauma with replication across two large, independent community-based samples
Source: Eur Psychiatry. 2023 Jan 26;66(1):e19. doi: 10.1192/j.eurpsy.2022.2347 (PMC9970154; doi:10.1192/j.eurpsy.2022.2347)
Supplement: Supplementary file 1 [file epasup.zip › S0924933822023471sup003.docx]

Appendix 2: Regression model results for the effect of CT score on structural MRI metrics across GS and UKB, and at mega-analysis. Metrics significantly associated with CT score after FDR correction are highlighted in bold; metrics significantly associated with CT score with replication across the three analyses are highlighted in yellow

Whole brain metrics

|  | **GS (n=1,024)** | | | **UKB (n=26,639)** | | | **Mega-Analysis (n=27,663)** | | |  |
| --- | --- | --- | --- | --- | --- | --- | --- | --- | --- | --- |
| **Region** | **Beta** | **Std Err** | **P(FDR)** | **Beta** | **Std Err** | **P(FDR)** | **Beta** | **Std Err** | **P(FDR)** |  |
| Global WM | -0.0852 | 0.0258 | **0.0015** | -0.0311 | 0.0049 | **2.67E-10** | -0.0330 | 0.0048 | **9.15E-12** |  |
| Global GM | -0.0682 | 0.0219 | **0.0019** | -0.0433 | 0.0049 | **5.27E-18** | -0.0442 | 0.0048 | **1.74E-19** |  |
| Whole Brain Vol | -0.0844 | 0.0239 | **0.0013** | -0.0399 | 0.0048 | **1.75E-16** | -0.0417 | 0.0047 | **1.51E-18** |  |
|  |  |  |  |  |  |  |  |  |  |  |

Lobar measures

|  | **GS (n=1,024)** | | | **UKB (n=27,202)** | | | **Mega-Analysis (n=28,226)** | | |  |
| --- | --- | --- | --- | --- | --- | --- | --- | --- | --- | --- |
| **Region** | **Beta** | **Std Err** | **P(FDR)** | **Beta** | **Std Err** | **P(FDR)** | **Beta** | **Std Err** | **P(FDR)** |  |
| **Volume** |  |  |  |  |  |  |  |  |  |  |
| Frontal | -0.0675 | 0.0237 | **0.017** | -0.0298 | 0.0049 | **2.45E-09** | -0.0365 | 0.0047 | **2.89E-14** |  |
| Temporal | -0.0553 | 0.0235 | **0.031** | -0.0351 | 0.0048 | **8.35E-13** | -0.0308 | 0.0048 | **2.84E-10** |  |
| Parietal | -0.0652 | 0.0240 | **0.017** | -0.0393 | 0.0050 | **3.42E-14** | -0.0404 | 0.0049 | **1.34E-15** |  |
| Occipital | -0.0528 | 0.0245 | **0.031** | -0.0189 | 0.0046 | **4.17E-05** | -0.0204 | 0.0045 | **6.82E-06** |  |
| Cingulate | -0.0520 | 0.0237 | **0.031** | -0.0205 | 0.0047 | **1.88E-05** | -0.0219 | 0.0046 | **3.05E-06** |  |
|  |  |  |  |  |  |  |  |  |  |  |
|  |  |  |  |  |  |  |  |  |  |  |
| **Thickness** |  |  |  |  |  |  |  |  |  |  |
| Frontal | -0.0251 | 0.0265 | 0.74 | -0.0093 | 0.0053 | 0.16 | -0.0091 | 0.0051 | 0.13 |  |
| Temporal | 0.0034 | 0.0263 | 0.90 | -0.0080 | 0.0052 | 0.16 | -0.0091 | 0.0052 | 0.13 |  |
| Parietal | -0.0292 | 0.0239 | 0.74 | -0.0113 | 0.0054 | 0.16 | -0.0124 | 0.0053 | 0.091 |  |
| Occipital | 0.0176 | 0.0263 | 0.74 | 0.0084 | 0.0054 | 0.16 | 0.0083 | 0.0053 | 0.15 |  |
| Cingulate | 0.0138 | 0.0258 | 0.74 | 0.0037 | 0.0050 | 0.46 | 0.0038 | 0.0049 | 0.44 |  |
|  |  |  |  |  |  |  |  |  |  |  |
|  |  |  |  |  |  |  |  |  |  |  |
| **Surf Area** |  |  |  |  |  |  |  |  |  |  |
| Frontal | -0.0647 | 0.0259 | **0.018** | -0.0296 | 0.0050 | **3.65E-09** | -0.0385 | 0.0048 | **5.43E-15** |  |
| Temporal | -0.0615 | 0.0250 | **0.018** | -0.0375 | 0.0049 | **8.60E-14** | -0.0308 | 0.0049 | **3.53E-10** |  |
| Parietal | -0.0622 | 0.0254 | **0.018** | -0.0377 | 0.0051 | **2.17E-13** | -0.0387 | 0.0050 | **1.56E-14** |  |
| Occipital | -0.0776 | 0.0256 | **0.012** | -0.0319 | 0.0051 | **7.32E-10** | -0.0337 | 0.0050 | **3.03E-11** |  |
| Cingulate | -0.0499 | 0.0250 | **0.046** | -0.0209 | 0.0047 | **7.98E-06** | -0.0221 | 0.0046 | **1.61E-06** |  |
|  |  |  |  |  |  |  |  |  |  |  |

Cortical volumes

|  | **GS (n=1,024)** | | | **UKB (n=27,202)** | | | **Mega-Analysis (n=28,226)** | | |  |
| --- | --- | --- | --- | --- | --- | --- | --- | --- | --- | --- |
| **Region** | **Beta** | **Std Err** | **P(FDR)** | **Beta** | **Std Err** | **P(FDR)** | **Beta** | **Std Err** | **P(FDR)** |  |
| Banks STS | 0.0088 | 0.0231 | 0.85 | -0.0166 | 0.0044 | **0.0016** | -0.0160 | 0.0043 | **0.0017** |  |
| Caudal Ant Cingulate | -0.0245 | 0.0212 | 0.76 | -0.0063 | 0.0042 | 0.24 | -0.0072 | 0.0042 | 0.17 |  |
| Caudal Mid Frontal | 0.0093 | 0.0234 | 0.85 | 0.0042 | 0.0043 | 0.44 | 0.0043 | 0.0043 | 0.41 |  |
| Cuneus | -0.0352 | 0.0237 | 0.76 | -0.0101 | 0.0050 | 0.11 | -0.0113 | 0.0049 | 0.055 |  |
| Entorhinal | -0.0230 | 0.0225 | 0.76 | -0.0017 | 0.0049 | 0.75 | -0.0030 | 0.0048 | 0.68 |  |
| Frontal Pole | -0.0216 | 0.0239 | 0.76 | 0.0023 | 0.0047 | 0.73 | 0.0017 | 0.0046 | 0.77 |  |
| Fusiform | -0.0207 | 0.0222 | 0.76 | -0.0070 | 0.0041 | 0.19 | -0.0075 | 0.0040 | 0.14 |  |
| Inf Parietal | -0.0184 | 0.0221 | 0.76 | -0.0223 | 0.0042 | **4.78E-06** | -0.0225 | 0.0042 | **2.43E-06** |  |
| Inf Temporal | 0.0086 | 0.0216 | 0.85 | -0.0107 | 0.0040 | **0.032** | -0.0101 | 0.0040 | **0.032** |  |
| Insula | -0.0183 | 0.0206 | 0.76 | -0.0102 | 0.0042 | **0.050** | -0.0107 | 0.0041 | **0.032** |  |
| Isthmus Cingulate | -0.0065 | 0.0230 | 0.85 | 0.0024 | 0.0043 | 0.71 | 0.0019 | 0.0043 | 0.73 |  |
| Lat Occipital | -0.0261 | 0.0215 | 0.76 | -0.0044 | 0.0043 | 0.43 | -0.0054 | 0.0042 | 0.28 |  |
| Lat Orbitofrontal | 0.0152 | 0.0210 | 0.76 | -0.0068 | 0.0041 | 0.19 | -0.0062 | 0.0040 | 0.23 |  |
| Lingual | 0.0258 | 0.0241 | 0.76 | 0.0018 | 0.0050 | 0.75 | 0.0022 | 0.0049 | 0.73 |  |
| Med Orbitofrontal | -0.0147 | 0.0205 | 0.76 | -0.0098 | 0.0040 | **0.050** | -0.0100 | 0.0039 | **0.032** |  |
| Middle Temporal | -0.0264 | 0.0204 | 0.76 | -0.0174 | 0.0040 | **0.00024** | -0.0179 | 0.0039 | **8.54E-05** |  |
| Paracentral | 0.0058 | 0.0227 | 0.85 | -0.0065 | 0.0044 | 0.24 | -0.0061 | 0.0044 | 0.26 |  |
| Parahippocampal | -0.0170 | 0.0245 | 0.76 | -0.0110 | 0.0050 | 0.074 | -0.0119 | 0.0049 | **0.040** |  |
| Pars Opercularis | 0.0219 | 0.0228 | 0.76 | -0.0028 | 0.0045 | 0.67 | -0.0019 | 0.0044 | 0.73 |  |
| Pars Orbitalis | -0.0263 | 0.0216 | 0.76 | -0.0045 | 0.0044 | 0.43 | -0.0052 | 0.0043 | 0.32 |  |
| Pars Triangularis | -0.0045 | 0.0227 | 0.85 | -0.0059 | 0.0045 | 0.30 | -0.0058 | 0.0044 | 0.28 |  |
| Pericalcarine | -0.0123 | 0.0261 | 0.85 | -0.0165 | 0.0054 | **0.011** | -0.0167 | 0.0053 | **0.0070** |  |
| Postcentral | -0.0041 | 0.0223 | 0.85 | -0.0070 | 0.0042 | 0.19 | -0.0071 | 0.0042 | 0.17 |  |
| Posterior Cingulate | 0.0120 | 0.0224 | 0.84 | 0.0001 | 0.0043 | 0.99 | 0.0002 | 0.0042 | 0.97 |  |
| Precentral | -0.0341 | 0.0225 | 0.76 | -0.0129 | 0.0043 | **0.011** | -0.0140 | 0.0042 | **0.0048** |  |
| Precuneus | -0.0324 | 0.0214 | 0.76 | -0.0149 | 0.0041 | **0.0025** | -0.0158 | 0.0041 | **0.0011** |  |
| Rostral Ant Cingulate | -0.0126 | 0.0215 | 0.83 | -0.0053 | 0.0042 | 0.30 | -0.0058 | 0.0041 | 0.26 |  |
| Rostral Mid Frontal | -0.0302 | 0.0201 | 0.76 | -0.0092 | 0.0039 | **0.056** | -0.0100 | 0.0038 | **0.032** |  |
| Sup Frontal | -0.0134 | 0.0196 | 0.76 | -0.0016 | 0.0038 | 0.75 | -0.0022 | 0.0038 | 0.69 |  |
| Sup Parietal | -0.0309 | 0.0223 | 0.76 | -0.0079 | 0.0044 | 0.18 | -0.0090 | 0.0044 | 0.093 |  |
| Sup Temporal | 0.0156 | 0.0216 | 0.76 | -0.0063 | 0.0041 | 0.23 | -0.0057 | 0.0041 | 0.26 |  |
| Supramarginal | 0.0049 | 0.0215 | 0.85 | -0.0141 | 0.0041 | **0.0038** | -0.0134 | 0.0040 | **0.0048** |  |
| Temporal Pole* | 0.0055 | 0.0245 | 0.85 |  |  |  |  |  |  |  |
| Transverse Temporal | 0.0179 | 0.0247 | 0.76 | -0.0021 | 0.0048 | 0.75 | -0.0015 | 0.0047 | 0.77 |  |
|  |  |  |  |  |  |  |  |  |  |  |

Cortical Surface Area

|  | **GS (n=1,024)** | | | **UKB (n=27,202)** | | | **Mega-Analysis (n=28,226)** | | |  |
| --- | --- | --- | --- | --- | --- | --- | --- | --- | --- | --- |
| **Region** | **Beta** | **Std Err** | **P(FDR)** | **Beta** | **Std Err** | **P(FDR)** | **Beta** | **Std Err** | **P(FDR)** |  |
| Banks STS | 0.0033 | 0.0233 | 0.97 | -0.0129 | 0.0043 | **0.014** | -0.0126 | 0.0043 | **0.012** |  |
| Caudal Ant Cingulate | -0.0123 | 0.0219 | 0.92 | -0.0041 | 0.0042 | 0.43 | -0.0045 | 0.0041 | 0.37 |  |
| Caudal Mid Frontal | 0.0094 | 0.0238 | 0.92 | 0.0039 | 0.0043 | 0.47 | 0.0041 | 0.0043 | 0.43 |  |
| Cuneus | -0.0653 | 0.0239 | 0.22 | -0.0172 | 0.0049 | **0.0035** | -0.0190 | 0.0048 | **0.00056** |  |
| Entorhinal | -0.0034 | 0.0236 | 0.97 | -0.0057 | 0.0049 | 0.34 | -0.0060 | 0.0048 | 0.30 |  |
| Frontal Pole | -0.0253 | 0.0231 | 0.85 | -0.0092 | 0.0042 | 0.062 | -0.0095 | 0.0041 | **0.048** |  |
| Fusiform | -0.0221 | 0.0224 | 0.92 | -0.0123 | 0.0039 | **0.0091** | -0.0125 | 0.0039 | **0.0069** |  |
| Inf Parietal | -0.0112 | 0.0227 | 0.92 | -0.0186 | 0.0042 | **0.00021** | -0.0185 | 0.0042 | **0.00014** |  |
| Inf Temporal | 0.0028 | 0.0224 | 0.97 | -0.0131 | 0.0040 | **0.0063** | -0.0126 | 0.0039 | **0.0069** |  |
| Insula | -0.0032 | 0.0211 | 0.97 | -0.0065 | 0.0040 | 0.18 | -0.0063 | 0.0039 | 0.18 |  |
| Isthmus Cingulate | -0.0092 | 0.0223 | 0.92 | -0.0009 | 0.0041 | 0.85 | -0.0013 | 0.0040 | 0.80 |  |
| Lat Occipital | -0.0310 | 0.0223 | 0.75 | -0.0121 | 0.0043 | **0.016** | -0.0129 | 0.0043 | **0.012** |  |
| Lat Orbitofrontal | 0.0108 | 0.0230 | 0.92 | -0.0076 | 0.0040 | 0.12 | -0.0069 | 0.0039 | 0.15 |  |
| Lingual | -0.0008 | 0.0245 | 0.97 | -0.0128 | 0.0049 | **0.026** | -0.0126 | 0.0048 | **0.022** |  |
| Med Orbitofrontal | -0.0101 | 0.0215 | 0.92 | -0.0053 | 0.0035 | 0.21 | -0.0055 | 0.0035 | 0.19 |  |
| Middle Temporal | -0.0322 | 0.0219 | 0.75 | -0.0161 | 0.0039 | **0.00042** | -0.0168 | 0.0039 | **0.00014** |  |
| Paracentral | 0.0270 | 0.0229 | 0.85 | 0.0028 | 0.0042 | 0.61 | 0.0036 | 0.0042 | 0.47 |  |
| Parahippocampal | -0.0271 | 0.0236 | 0.85 | -0.0104 | 0.0044 | **0.040** | -0.0113 | 0.0043 | **0.022** |  |
| Pars Opercularis | 0.0122 | 0.0236 | 0.92 | 0.0011 | 0.0045 | 0.85 | 0.0016 | 0.0044 | 0.78 |  |
| Pars Orbitalis | -0.0307 | 0.0227 | 0.75 | -0.0052 | 0.0042 | 0.32 | -0.0060 | 0.0041 | 0.23 |  |
| Pars Triangularis | -0.0017 | 0.0243 | 0.97 | -0.0037 | 0.0045 | 0.51 | -0.0034 | 0.0044 | 0.52 |  |
| Pericalcarine | -0.0583 | 0.0263 | 0.46 | -0.0248 | 0.0054 | **0.00016** | -0.0262 | 0.0053 | **2.75E-05** |  |
| Postcentral | -0.0135 | 0.0227 | 0.92 | -0.0108 | 0.0039 | **0.016** | -0.0110 | 0.0038 | **0.014** |  |
| Posterior Cingulate | 0.0100 | 0.0222 | 0.92 | -0.0011 | 0.0040 | 0.85 | -0.0008 | 0.0039 | 0.87 |  |
| Precentral | -0.0318 | 0.0222 | 0.75 | -0.0074 | 0.0040 | 0.12 | -0.0083 | 0.0039 | 0.073 |  |
| Precuneus | -0.0142 | 0.0221 | 0.92 | -0.0119 | 0.0041 | **0.015** | -0.0121 | 0.0041 | **0.012** |  |
| Rostral Ant Cingulate | 0.0024 | 0.0220 | 0.97 | 0.0022 | 0.0040 | 0.66 | 0.0021 | 0.0039 | 0.68 |  |
| Rostral Mid Frontal | -0.0305 | 0.0221 | 0.75 | -0.0095 | 0.0039 | **0.036** | -0.0103 | 0.0038 | **0.021** |  |
| Sup Frontal | 0.0064 | 0.0211 | 0.96 | -0.0002 | 0.0037 | 0.97 | 0.0000 | 0.0037 | 1.00 |  |
| Sup Parietal | -0.0382 | 0.0237 | 0.75 | -0.0072 | 0.0044 | 0.18 | -0.0083 | 0.0043 | 0.11 |  |
| Sup Temporal | 0.0083 | 0.0217 | 0.92 | -0.0059 | 0.0039 | 0.21 | -0.0054 | 0.0038 | 0.23 |  |
| Supramarginal | 0.0163 | 0.0222 | 0.92 | -0.0116 | 0.0040 | **0.015** | -0.0105 | 0.0040 | **0.022** |  |
| Temporal Pole* | 0.0093 | 0.0227 | 0.92 |  |  |  |  |  |  |  |
| Transverse Temporal | 0.0128 | 0.0251 | 0.92 | -0.0054 | 0.0046 | 0.34 | -0.0043 | 0.0045 | 0.43 |  |
|  |  |  |  |  |  |  |  |  |  |  |

Cortical Thickness

|  | **GS (n=1,024)** | | | **UKB (n=27,202)** | | | **Mega-Analysis (n=28,226)** | | |  |
| --- | --- | --- | --- | --- | --- | --- | --- | --- | --- | --- |
| **Region** | **Beta** | **Std Err** | **P(FDR)** | **Beta** | **Std Err** | **P(FDR)** | **Beta** | **Std Err** | **P(FDR)** |  |
| Banks STS | 0.0176 | 0.0250 | 0.96 | -0.0127 | 0.0050 | 0.091 | -0.0121 | 0.0049 | 0.064 |  |
| Caudal Ant Cingulate | -0.0313 | 0.0245 | 0.86 | -0.0011 | 0.0047 | 0.89 | -0.0021 | 0.0047 | 0.78 |  |
| Caudal Mid Frontal | -0.0137 | 0.0257 | 0.96 | -0.0061 | 0.0053 | 0.41 | -0.0068 | 0.0052 | 0.35 |  |
| Cuneus | 0.0228 | 0.0260 | 0.86 | 0.0063 | 0.0054 | 0.41 | 0.0066 | 0.0053 | 0.36 |  |
| Entorhinal | -0.0293 | 0.0266 | 0.86 | 0.0028 | 0.0051 | 0.74 | 0.0014 | 0.0050 | 0.86 |  |
| Frontal Pole | 0.0120 | 0.0251 | 0.96 | 0.0079 | 0.0050 | 0.25 | 0.0080 | 0.0049 | 0.21 |  |
| Fusiform | 0.0064 | 0.0270 | 0.96 | 0.0007 | 0.0052 | 0.94 | 0.0004 | 0.0051 | 0.95 |  |
| Inf Parietal | -0.0238 | 0.0240 | 0.86 | -0.0156 | 0.0053 | **0.050** | -0.0163 | 0.0051 | **0.025** |  |
| Inf Temporal | 0.0030 | 0.0252 | 0.96 | -0.0064 | 0.0052 | 0.39 | -0.0063 | 0.0051 | 0.36 |  |
| Insula | -0.0105 | 0.0257 | 0.96 | -0.0018 | 0.0051 | 0.85 | -0.0027 | 0.0050 | 0.75 |  |
| Isthmus Cingulate | 0.0228 | 0.0253 | 0.86 | 0.0028 | 0.0051 | 0.74 | 0.0035 | 0.0050 | 0.64 |  |
| Lat Occipital | -0.0122 | 0.0255 | 0.96 | 0.0124 | 0.0054 | 0.091 | 0.0112 | 0.0053 | 0.096 |  |
| Lat Orbitofrontal | -0.0053 | 0.0279 | 0.96 | -0.0017 | 0.0053 | 0.85 | -0.0021 | 0.0052 | 0.78 |  |
| Lingual | 0.0581 | 0.0264 | 0.48 | 0.0199 | 0.0054 | **0.0067** | 0.0208 | 0.0053 | **0.0026** |  |
| Med Orbitofrontal | -0.0263 | 0.0264 | 0.86 | -0.0080 | 0.0051 | 0.25 | -0.0089 | 0.0050 | 0.18 |  |
| Middle Temporal | 0.0018 | 0.0246 | 0.97 | -0.0092 | 0.0051 | 0.18 | -0.0093 | 0.0050 | 0.16 |  |
| Paracentral | -0.0473 | 0.0248 | 0.59 | -0.0123 | 0.0053 | 0.091 | -0.0139 | 0.0052 | 0.058 |  |
| Parahippocampal | 0.0095 | 0.0276 | 0.96 | -0.0047 | 0.0052 | 0.55 | -0.0046 | 0.0051 | 0.55 |  |
| Pars Opercularis | 0.0069 | 0.0242 | 0.96 | -0.0111 | 0.0052 | 0.12 | -0.0109 | 0.0051 | 0.092 |  |
| Pars Orbitalis | -0.0057 | 0.0249 | 0.96 | -0.0035 | 0.0050 | 0.68 | -0.0038 | 0.0049 | 0.61 |  |
| Pars Triangularis | 0.0010 | 0.0253 | 0.97 | -0.0131 | 0.0052 | 0.091 | -0.0130 | 0.0051 | 0.058 |  |
| Pericalcarine | 0.0725 | 0.0271 | 0.26 | 0.0097 | 0.0053 | 0.18 | 0.0115 | 0.0052 | 0.092 |  |
| Postcentral | 0.0079 | 0.0239 | 0.96 | -0.0002 | 0.0052 | 0.99 | -0.0003 | 0.0051 | 0.95 |  |
| Posterior Cingulate | 0.0241 | 0.0258 | 0.86 | -0.0001 | 0.0049 | 0.99 | 0.0003 | 0.0048 | 0.95 |  |
| Precentral | -0.0177 | 0.0270 | 0.96 | -0.0130 | 0.0053 | 0.091 | -0.0137 | 0.0052 | 0.058 |  |
| Precuneus | -0.0432 | 0.0252 | 0.59 | -0.0072 | 0.0054 | 0.35 | -0.0091 | 0.0053 | 0.19 |  |
| Rostral Ant Cingulate | -0.0393 | 0.0256 | 0.71 | -0.0091 | 0.0048 | 0.18 | -0.0102 | 0.0047 | 0.092 |  |
| Rostral Mid Frontal | -0.0136 | 0.0257 | 0.96 | -0.0046 | 0.0053 | 0.56 | -0.0052 | 0.0052 | 0.51 |  |
| Sup Frontal | -0.0466 | 0.0266 | 0.59 | -0.0096 | 0.0053 | 0.18 | -0.0114 | 0.0052 | 0.092 |  |
| Sup Parietal | -0.0117 | 0.0244 | 0.96 | -0.0018 | 0.0054 | 0.85 | -0.0026 | 0.0053 | 0.75 |  |
| Sup Temporal | 0.0245 | 0.0260 | 0.86 | -0.0086 | 0.0051 | 0.22 | -0.0078 | 0.0050 | 0.24 |  |
| Supramarginal | -0.0231 | 0.0244 | 0.86 | -0.0122 | 0.0052 | 0.091 | -0.0132 | 0.0051 | 0.058 |  |
| Temporal Pole* | 0.0037 | 0.0269 | 0.96 |  |  |  |  |  |  |  |
| Transverse Temporal | -0.0033 | 0.0266 | 0.96 | 0.0049 | 0.0053 | 0.55 | 0.0040 | 0.0052 | 0.61 |  |
|  |  |  |  |  |  |  |  |  |  |  |

Subcortical Volumes

|  | **GS (n=1,024)** | | | **UKB (n=27,202)** | | | **Mega-Analysis (n=28,226)** | | |  |
| --- | --- | --- | --- | --- | --- | --- | --- | --- | --- | --- |
| **Region** | **Beta** | **Std Err** | **P(FDR)** | **Beta** | **Std Err** | **P(FDR)** | **Beta** | **Std Err** | **P(FDR)** |  |
| Accumbens | -0.0654 | 0.0231 | **0.037** | -0.0035 | 0.0042 | 0.47 | -0.0053 | 0.0041 | 0.23 |  |
| Amygdala | -0.0136 | 0.0244 | 0.75 | 0.0021 | 0.0040 | 0.60 | 0.0017 | 0.0039 | 0.66 |  |
| Caudate | -0.0081 | 0.0258 | 0.75 | 0.0104 | 0.0050 | 0.075 | 0.0096 | 0.0049 | 0.080 |  |
| Hippocampus | -0.0582 | 0.0247 | **0.050** | -0.0101 | 0.0044 | 0.062 | -0.0119 | 0.0044 | **0.017** |  |
| Pallidum | -0.0249 | 0.0240 | 0.53 | -0.0087 | 0.0044 | 0.082 | -0.0093 | 0.0044 | 0.070 |  |
| Putamen | -0.0101 | 0.0244 | 0.75 | 0.0085 | 0.0048 | 0.099 | 0.0080 | 0.0047 | 0.12 |  |
| Thalamus | -0.0201 | 0.0207 | 0.53 | -0.0138 | 0.0040 | **0.0024** | -0.0134 | 0.0039 | **0.0026** |  |
| Ventral DC | -0.0526 | 0.0218 | **0.050** | -0.0220 | 0.0040 | **2.84E-07** | -0.0232 | 0.0039 | **2.91E-08** |  |
|  |  |  |  |  |  |  |  |  |  |  |
