# Supplementary material for: Structural brain correlates of childhood trauma with replication across two large, independent community-based samples
Source: Eur Psychiatry. 2023 Jan 26;66(1):e19. doi: 10.1192/j.eurpsy.2022.2347 (PMC9970154; doi:10.1192/j.eurpsy.2022.2347)
Supplement: Supplementary file 1 [file epasup.zip › S0924933822023471sup004.docx]

Appendix 3: Results of the trauma subscale and abuse and neglect composite scores, regressed against structural MRI metrics.

Whole Brain Volumes

| **Emotional Abuse** | | **GS (n=1,024)** | | | | | **UKB (n=27,202)** | | | | | | **Mega-Analysis (n=27,663)** | | | | | |  | |
| --- | --- | --- | --- | --- | --- | --- | --- | --- | --- | --- | --- | --- | --- | --- | --- | --- | --- | --- | --- | --- |
| **Region** | | **Beta** | **Std Err** | | **P(FDR)** | | **Beta** | | **Std Err** | | **P(FDR)** | | **Beta** | | **Std Err** | | **P(FDR)** | |  | |
| Global WM | | -0.0805 | 0.0261 | | **0.0062** | | -0.0100 | | 0.0049 | | **0.043** | | -0.0124 | | 0.0049 | | **0.011** | |  | |
| Global GM | | -0.0480 | 0.0222 | | **0.031** | | -0.0244 | | 0.0050 | | **2.55E-06** | | -0.0253 | | 0.0049 | | **5.84E-07** | |  | |
| Whole Brain Vol | | -0.0697 | 0.0243 | | **0.0062** | | -0.0176 | | 0.0048 | | **4.14E-04** | | -0.0196 | | 0.0047 | | **5.27E-05** | |  | |
|  | |  |  | |  | |  | |  | |  | |  | |  | |  | |  | |
|  | |  |  | |  | |  | |  | |  | |  | |  | |  | |  | |
| **Physical Abuse** | | **GS (n=1,024)** | | | | | **UKB (n=27,202)** | | | | | | **Mega-Analysis (n=27,663)** | | | | | |  | |
| **Region** | | **Beta** | **Std Err** | | **P(FDR)** | | **Beta** | | **Std Err** | | **P(FDR)** | | **Beta** | | **Std Err** | | **P(FDR)** | |  | |
| Global WM | | -0.1135 | 0.0257 | | **2.42E-05** | | -0.0274 | | 0.0049 | | **2.77E-08** | | -0.0306 | | 0.0048 | | **2.32E-10** | |  | |
| Global GM | | -0.0765 | 0.0219 | | **4.86E-04** | | -0.0329 | | 0.0049 | | **8.61E-11** | | -0.0341 | | 0.0048 | | **2.55E-12** | |  | |
| Whole Brain Vol | | -0.1033 | 0.0238 | | **2.42E-05** | | -0.0311 | | 0.0048 | | **1.59E-10** | | -0.0339 | | 0.0047 | | **1.86E-12** | |  | |
|  | |  |  | |  | |  | |  | |  | |  | |  | |  | |  | |
|  | |  |  | |  | |  | |  | |  | |  | |  | |  | |  | |
| **Sexual Abuse** | | **GS (n=1,024)** | | | | | **UKB (n=27,202)** | | | | | | **Mega-Analysis (n=27,663)** | | | | | |  | |
| **Region** | | **Beta** | **Std Err** | | **P(FDR)** | | **Beta** | | **Std Err** | | **P(FDR)** | | **Beta** | | **Std Err** | | **P(FDR)** | |  | |
| Global WM | | -0.0685 | 0.0261 | | **0.013** | | -0.0185 | | 0.0049 | | **2.73E-04** | | -0.0201 | | 0.0048 | | **5.11E-05** | |  | |
| Global GM | | -0.0536 | 0.0222 | | **0.016** | | -0.0165 | | 0.0050 | | **8.96E-04** | | -0.0179 | | 0.0048 | | **2.14E-04** | |  | |
| Whole Brain Vol | | -0.0673 | 0.0243 | | **0.013** | | -0.0191 | | 0.0048 | | **2.32E-04** | | -0.0208 | | 0.0047 | | **3.42E-05** | |  | |
|  | |  |  | |  | |  | |  | |  | |  | |  | |  | |  | |
| **Emotional Neglect** | **GS (n=1,024)** | | | | | | | **UKB (n=27,202)** | | | | | | **Mega-Analysis (n=27,663)** | | | | | |  |
| **Region** | **Beta** | | | **Std Err** | | **P(FDR)** | | **Beta** | | **Std Err** | | **P(FDR)** | | **Beta** | | **Std Err** | | **P(FDR)** | |  |
| Global WM | -0.0254 | | | 0.0259 | | 0.33 | | -0.0154 | | 0.0049 | | **0.0017** | | -0.0157 | | 0.0048 | | **0.0011** | |  |
| Global GM | -0.0451 | | | 0.0220 | | 0.12 | | -0.0258 | | 0.0049 | | **5.25E-07** | | -0.0264 | | 0.0048 | | **1.27E-07** | |  |
| Whole Brain Vol | -0.0399 | | | 0.0240 | | 0.15 | | -0.0227 | | 0.0048 | | **3.30E-06** | | -0.0234 | | 0.0047 | | **9.99E-07** | |  |
|  |  | | |  | |  | |  | |  | |  | |  | |  | |  | |  |
|  |  | | |  | |  | |  | |  | |  | |  | |  | |  | |  |
| **Physical Neglect** | **GS (n=1,024)** | | | | | | | **UKB (n=27,202)** | | | | | | **Mega-Analysis (n=27,663)** | | | | | |  |
| **Region** | **Beta** | | | **Std Err** | | **P(FDR)** | | **Beta** | | **Std Err** | | **P(FDR)** | | **Beta** | | **Std Err** | | **P(FDR)** | |  |
| Global WM | -0.0466 | | | 0.0259 | | 0.11 | | -0.0321 | | 0.0049 | | **6.98E-11** | | -0.0326 | | 0.0048 | | **1.61E-11** | |  |
| Global GM | -0.0333 | | | 0.0220 | | 0.13 | | -0.0398 | | 0.0049 | | **1.25E-15** | | -0.0394 | | 0.0048 | | **5.15E-16** | |  |
| Whole Brain Vol | -0.0443 | | | 0.0241 | | 0.11 | | -0.0393 | | 0.0048 | | **9.31E-16** | | -0.0398 | | 0.0047 | | **1.04E-16** | |  |
|  |  | | |  | |  | |  | |  | |  | |  | |  | |  | |  |

|  |  | |  | |  | |  |  | |  | |  |  |  | |  |
| --- | --- | --- | --- | --- | --- | --- | --- | --- | --- | --- | --- | --- | --- | --- | --- | --- |
| **Abuse Composite Score** | **GS (n=1,024)** | | | | | | **UKB (n=27,202)** | | | | | **Mega-Analysis (n=27,663)** | | | |  |
| **Region** | **Beta** | | **Std Err** | | **P(FDR)** | | **Beta** | **Std Err** | | **P(FDR)** | | **Beta** | **Std Err** | **P(FDR)** | |  |
| Global WM | -0.1080 | | 0.0259 | | **6.17E-05** | | -0.0252 | 0.0049 | | **3.46E-07** | | -0.0281 | 0.0049 | **7.32E-09** | |  |
| Global GM | -0.0735 | | 0.0221 | | **8.92E-04** | | -0.0347 | 0.0050 | | **8.75E-12** | | -0.0359 | 0.0048 | **3.73E-13** | |  |
| Whole Brain Vol | -0.0991 | | 0.0241 | | **6.17E-05** | | -0.0312 | 0.0048 | | **1.69E-10** | | -0.0338 | 0.0047 | **1.52E-12** | |  |
|  |  | |  | |  | |  |  | |  | |  |  |  | |  |
|  | |  | |  | |  |  | |  | |  |  |  | |  |  |
| **Neglect Composite Score** | | **GS (n=1,024)** | | | | | **UKB (n=27,202)** | | | | | **Mega-Analysis (n=27,663)** | | | |  |
| **Region** | | **Beta** | | **Std Err** | | **P(FDR)** | **Beta** | | **Std Err** | | **P(FDR)** | **Beta** | **Std Err** | | **P(FDR)** |  |
| Global WM | | -0.0366 | | 0.0259 | | 0.16 | -0.0277 | | 0.0049 | | **1.73E-08** | -0.0280 | 0.0048 | | **6.86E-09** |  |
| Global GM | | -0.0456 | | 0.0219 | | 0.082 | -0.0391 | | 0.0049 | | **6.83E-15** | -0.0392 | 0.0048 | | **1.15E-15** |  |
| Whole Brain Vol | | -0.0462 | | 0.0240 | | 0.082 | -0.0367 | | 0.0048 | | **3.29E-14** | -0.0372 | 0.0047 | | **4.24E-15** |  |
|  | |  | |  | |  |  | |  | |  |  |  | |  |  |

Lobar volume

| **Emotional Abuse** | **GS (n=1,024)** | | | | | | **UKB (n=27,202)** | | | | | | **Mega-Analysis (n=28,226)** | | | | | |  | |
| --- | --- | --- | --- | --- | --- | --- | --- | --- | --- | --- | --- | --- | --- | --- | --- | --- | --- | --- | --- | --- |
| **Region** | **Beta** | | **Std Err** | | **P(FDR)** | | **Beta** | | **Std Err** | | **P(FDR)** | | **Beta** | | **Std Err** | | **P(FDR)** | |  | |
| Frontal Lobe | -0.0453 | | 0.0241 | | 0.10 | | -0.0155 | | 0.0050 | | **0.0029** | | -0.0211 | | 0.0047 | | **2.25E-05** | |  | |
| Temporal Lobe | -0.0394 | | 0.0238 | | 0.10 | | -0.0201 | | 0.0048 | | **8.40E-05** | | -0.0165 | | 0.0049 | | **0.0011** | |  | |
| Parietal Lobe | -0.0437 | | 0.0243 | | 0.10 | | -0.0225 | | 0.0051 | | **4.46E-05** | | -0.0233 | | 0.0050 | | **1.28E-05** | |  | |
| Occipital Lobe | -0.0411 | | 0.0248 | | 0.10 | | -0.0079 | | 0.0046 | | 0.11 | | -0.0094 | | 0.0046 | | **0.049** | |  | |
| Cingulate Lobe | -0.0396 | | 0.0240 | | 0.10 | | -0.0040 | | 0.0048 | | 0.40 | | -0.0055 | | 0.0047 | | 0.24 | |  | |
|  |  | |  | |  | |  | |  | |  | |  | |  | |  | |  | |
|  |  | |  | |  | |  | |  | |  | |  | |  | |  | |  | |
| **Physical Abuse** | **GS (n=1,024)** | | | | | | **UKB (n=27,202)** | | | | | | **Mega-Analysis (n=28,226)** | | | | | |  | |
| **Region** | **Beta** | | **Std Err** | | **P(FDR)** | | **Beta** | | **Std Err** | | **P(FDR)** | | **Beta** | | **Std Err** | | **P(FDR)** | |  | |
| Frontal Lobe | -0.0844 | | 0.0237 | | **9.51E-04** | | -0.0215 | | 0.0049 | | **1.72E-05** | | -0.0271 | | 0.0047 | | **2.5E-08** | |  | |
| Temporal Lobe | -0.0575 | | 0.0235 | | **0.018** | | -0.0248 | | 0.0048 | | **6.68E-07** | | -0.0229 | | 0.0048 | | **2.95E-06** | |  | |
| Parietal Lobe | -0.0930 | | 0.0239 | | **5.31E-04** | | -0.0330 | | 0.0050 | | **3.20E-10** | | -0.0354 | | 0.0049 | | **4.28E-12** | |  | |
| Occipital Lobe | -0.0431 | | 0.0245 | | 0.079 | | -0.0202 | | 0.0046 | | **1.72E-05** | | -0.0215 | | 0.0045 | | **2.95E-06** | |  | |
| Cingulate Lobe | -0.0591 | | 0.0237 | | **0.018** | | -0.0195 | | 0.0047 | | **3.81E-05** | | -0.0212 | | 0.0047 | | **5.05E-06** | |  | |
|  |  | |  | |  | |  | |  | |  | |  | |  | |  | |  | |
|  |  | |  | |  | |  | |  | |  | |  | |  | |  | |  | |
| **Sexual Abuse** | **GS (n=1,024)** | | | | | | **UKB (n=27,202)** | | | | | | **Mega-Analysis (n=28,226)** | | | | | |  | |
| **Region** | **Beta** | | **Std Err** | | **P(FDR)** | | **Beta** | | **Std Err** | | **P(FDR)** | | **Beta** | | **Std Err** | | **P(FDR)** | |  | |
| Frontal Lobe | -0.0557 | | 0.0240 | | 0.052 | | -0.0061 | | 0.0050 | | 0.22 | | -0.0139 | | 0.0048 | | **0.018** | |  | |
| Temporal Lobe | -0.0408 | | 0.0238 | | 0.11 | | -0.0123 | | 0.0048 | | 0.054 | | -0.0073 | | 0.0049 | | 0.13 | |  | |
| Parietal Lobe | -0.0321 | | 0.0243 | | 0.19 | | -0.0115 | | 0.0051 | | 0.058 | | -0.0122 | | 0.0050 | | **0.030** | |  | |
| Occipital Lobe | -0.0535 | | 0.0248 | | 0.052 | | -0.0074 | | 0.0046 | | 0.14 | | -0.0091 | | 0.0046 | | 0.058 | |  | |
| Cingulate Lobe | -0.0639 | | 0.0240 | | **0.039** | | -0.0091 | | 0.0048 | | 0.093 | | -0.0110 | | 0.0047 | | **0.030** | |  | |
|  |  | |  | |  | |  | |  | |  | |  | |  | |  | |  | |
|  |  | |  | |  | |  | |  | |  | |  | |  | |  | |  | |
| **Emotional Neglect** | | **GS (n=1,024)** | | | | | | **UKB (n=27,202)** | | | | | | **Mega-Analysis (n=28,226)** | | | | | |  |
| **Region** | | **Beta** | | **Std Err** | | **P(FDR)** | | **Beta** | | **Std Err** | | **P(FDR)** | | **Beta** | | **Std Err** | | **P(FDR)** | |  |
| Frontal Lobe | | -0.0442 | | 0.0238 | | 0.11 | | -0.0214 | | 0.0049 | | **2.22E-05** | | -0.0233 | | 0.0047 | | **2.05E-06** | |  |
| Temporal Lobe | | -0.0428 | | 0.0235 | | 0.11 | | -0.0223 | | 0.0048 | | **8.97E-06** | | -0.0222 | | 0.0048 | | **6.94E-06** | |  |
| Parietal Lobe | | -0.0480 | | 0.0240 | | 0.11 | | -0.0241 | | 0.0050 | | **8.69E-06** | | -0.0251 | | 0.0049 | | **1.73E-06** | |  |
| Occipital Lobe | | -0.0354 | | 0.0245 | | 0.19 | | -0.0056 | | 0.0046 | | 0.23 | | -0.0067 | | 0.0045 | | 0.14 | |  |
| Cingulate Lobe | | -0.0300 | | 0.0238 | | 0.21 | | -0.0153 | | 0.0047 | | **0.0015** | | -0.0159 | | 0.0046 | | **7.60E-04** | |  |
|  | |  | |  | |  | |  | |  | |  | |  | |  | |  | |  |
|  | |  | |  | |  | |  | |  | |  | |  | |  | |  | |  |
| **Physical Neglect** | | **GS (n=1,024)** | | | | | | **UKB (n=27,202)** | | | | | | **Mega-Analysis (n=28,226)** | | | | | |  |
| **Region** | | **Beta** | | **Std Err** | | **P(FDR)** | | **Beta** | | **Std Err** | | **P(FDR)** | | **Beta** | | **Std Err** | | **P(FDR)** | |  |
| Frontal Lobe | | -0.0235 | | 0.0238 | | 0.54 | | -0.0286 | | 0.0049 | | **1.12E-08** | | -0.0326 | | 0.0047 | | **1.43E-11** | |  |
| Temporal Lobe | | -0.0237 | | 0.0236 | | 0.54 | | -0.0324 | | 0.0048 | | **4.29E-11** | | -0.0286 | | 0.0048 | | **5.63E-09** | |  |
| Parietal Lobe | | -0.0350 | | 0.0241 | | 0.54 | | -0.0342 | | 0.0050 | | **4.29E-11** | | -0.0347 | | 0.0049 | | **1.08E-11** | |  |
| Occipital Lobe | | -0.0178 | | 0.0246 | | 0.59 | | -0.0222 | | 0.0046 | | **1.84E-06** | | -0.0224 | | 0.0045 | | **9.87E-07** | |  |
| Cingulate Lobe | | 0.0090 | | 0.0239 | | 0.71 | | -0.0177 | | 0.0047 | | **1.87E-04** | | -0.0171 | | 0.0046 | | **2.27E-04** | |  |
|  | |  | |  | |  | |  | |  | |  | |  | |  | |  | |  |

|  | |  |  | |  | |  | |  | |  | |  |  |  | |  | |
| --- | --- | --- | --- | --- | --- | --- | --- | --- | --- | --- | --- | --- | --- | --- | --- | --- | --- | --- |
| **Abuse Composite Score** | | **GS (n=1,024)** | | | | | **UKB (n=27,202)** | | | | | | **Mega-Analysis (n=28,226)** | | | |  | |
| **Region** | | **Beta** | **Std Err** | | **P(FDR)** | | **Beta** | | **Std Err** | | **P(FDR)** | | **Beta** | **Std Err** | **P(FDR)** | |  | |
| Frontal Lobe | | -0.0759 | 0.0239 | | **0.0077** | | -0.0197 | | 0.0050 | | **1.01E-04** | | -0.0288 | 0.0047 | **3.36E-09** | |  | |
| Temporal Lobe | | -0.0571 | 0.0237 | | **0.017** | | -0.0271 | | 0.0049 | | **8.46E-08** | | -0.0221 | 0.0049 | **8.92E-06** | |  | |
| Parietal Lobe | | -0.0666 | 0.0242 | | **0.010** | | -0.0294 | | 0.0051 | | **3.76E-08** | | -0.0331 | 0.0050 | **1.2E-10** | |  | |
| Occipital Lobe | | -0.0592 | 0.0247 | | **0.017** | | -0.0270 | | 0.0051 | | **2.33E-07** | | -0.0185 | 0.0046 | **6.43E-05** | |  | |
| Cingulate Lobe | | -0.0689 | 0.0239 | | **0.0099** | | -0.0143 | | 0.0047 | | **0.0023** | | -0.0170 | 0.0047 | **2.74E-04** | |  | |
|  | |  |  | |  | |  | |  | |  | |  |  |  | |  | |
|  | |  |  | |  | |  | |  | |  | |  |  |  | |  | |
| **Neglect Composite Score** | **GS (n=1,024)** | | | | | | | **UKB (n=27,202)** | | | | | **Mega-Analysis (n=28,226)** | | | |  |  |
| **Region** | **Beta** | | | **Std Err** | | **P(FDR)** | | **Beta** | | **Std Err** | | **P(FDR)** | **Beta** | **Std Err** | | **P(FDR)** |  |  |
| Frontal Lobe | -0.0411 | | | 0.0238 | | 0.15 | | -0.0307 | | 0.0050 | | **1.11E-09** | -0.0334 | 0.0047 | | **3.43E-12** |  |  |
| Temporal Lobe | -0.0402 | | | 0.0235 | | 0.15 | | -0.0368 | | 0.0049 | | **2.43E-13** | -0.0306 | 0.0048 | | **3.71E-10** |  |  |
| Parietal Lobe | -0.0484 | | | 0.0240 | | 0.15 | | -0.0349 | | 0.0051 | | **1.26E-11** | -0.0358 | 0.0049 | | **1.85E-12** |  |  |
| Occipital Lobe | -0.0326 | | | 0.0245 | | 0.23 | | -0.0272 | | 0.0051 | | **1.23E-07** | -0.0163 | 0.0045 | | **3.18E-04** |  |  |
| Cingulate Lobe | -0.0182 | | | 0.0238 | | 0.44 | | -0.0214 | | 0.0047 | | **4.98E-06** | -0.0203 | 0.0046 | | **1.53E-05** |  |  |
|  |  | | |  | |  | |  | |  | |  |  |  | |  |  |  |

Lobar Surface Area

| **Emotional Abuse** | **GS (n=1,024)** | | | | | | **UKB (n=27,202)** | | | | | | **Mega-Analysis (n=28,226)** | | | | | |  | |
| --- | --- | --- | --- | --- | --- | --- | --- | --- | --- | --- | --- | --- | --- | --- | --- | --- | --- | --- | --- | --- |
| **Region** | **Beta** | | **Std Err** | | **P(FDR)** | | **Beta** | | **Std Err** | | **P(FDR)** | | **Beta** | | **Std Err** | | **P(FDR)** | |  | |
| Frontal Lobe | -0.0490 | | 0.0263 | | 0.072 | | -0.0105 | | 0.0050 | | **0.046** | | -0.0191 | | 0.0048 | | **3.73E-04** | |  | |
| Temporal Lobe | -0.0485 | | 0.0254 | | 0.072 | | -0.0180 | | 0.0049 | | **0.0013** | | -0.0120 | | 0.0049 | | **0.019** | |  | |
| Parietal Lobe | -0.0484 | | 0.0257 | | 0.072 | | -0.0175 | | 0.0051 | | **0.0015** | | -0.0187 | | 0.0050 | | **4.40E-04** | |  | |
| Occipital Lobe | -0.0700 | | 0.0259 | | **0.035** | | -0.0126 | | 0.0051 | | **0.023** | | -0.0149 | | 0.0050 | | **0.0051** | |  | |
| Cingulate Lobe | -0.0455 | | 0.0253 | | 0.072 | | -0.0011 | | 0.0047 | | 0.82 | | -0.0028 | | 0.0046 | | 0.55 | |  | |
|  |  | |  | |  | |  | |  | |  | |  | |  | |  | |  | |
|  |  | |  | |  | |  | |  | |  | |  | |  | |  | |  | |
| **Physical Abuse** | **GS (n=1,024)** | | | | | | **UKB (n=27,202)** | | | | | | **Mega-Analysis (n=28,226)** | | | | | |  | |
| **Region** | **Beta** | | **Std Err** | | **P(FDR)** | | **Beta** | | **Std Err** | | **P(FDR)** | | **Beta** | | **Std Err** | | **P(FDR)** | |  | |
| Frontal Lobe | -0.0884 | | 0.0258 | | **0.0016** | | -0.0215 | | 0.0050 | | **1.92E-05** | | -0.0284 | | 0.0048 | | **6.12E-09** | |  | |
| Temporal Lobe | -0.0745 | | 0.0250 | | **0.0037** | | -0.0261 | | 0.0049 | | **1.62E-07** | | -0.0237 | | 0.0049 | | **1.57E-06** | |  | |
| Parietal Lobe | -0.1011 | | 0.0252 | | **3.22E-04** | | -0.0311 | | 0.0051 | | **4.25E-09** | | -0.0338 | | 0.0050 | | **5.48E-11** | |  | |
| Occipital Lobe | -0.0802 | | 0.0255 | | **0.0029** | | -0.0289 | | 0.0051 | | **4.2E-08** | | -0.0309 | | 0.0050 | | **1.73E-09** | |  | |
| Cingulate Lobe | -0.0679 | | 0.0249 | | **0.0066** | | -0.0201 | | 0.0047 | | **1.92E-05** | | -0.0220 | | 0.0046 | | **1.83E-06** | |  | |
|  |  | |  | |  | |  | |  | |  | |  | |  | |  | |  | |
|  |  | |  | |  | |  | |  | |  | |  | |  | |  | |  | |
| **Sexual Abuse** | **GS (n=1,024)** | | | | | | **UKB (n=27,202)** | | | | | | **Mega-Analysis (n=28,226)** | | | | | |  | |
| **Region** | **Beta** | | **Std Err** | | **P(FDR)** | | **Beta** | | **Std Err** | | **P(FDR)** | | **Beta** | | **Std Err** | | **P(FDR)** | |  | |
| Frontal Lobe | -0.0613 | | 0.0262 | | **0.033** | | -0.0108 | | 0.0050 | | **0.032** | | -0.0158 | | 0.0048 | | **0.0027** | |  | |
| Temporal Lobe | -0.0549 | | 0.0253 | | **0.038** | | -0.0141 | | 0.0049 | | **0.0075** | | -0.0122 | | 0.0049 | | **0.013** | |  | |
| Parietal Lobe | -0.0441 | | 0.0257 | | 0.086 | | -0.0145 | | 0.0051 | | **0.0075** | | -0.0155 | | 0.0050 | | **0.0032** | |  | |
| Occipital Lobe | -0.0688 | | 0.0259 | | **0.020** | | -0.0179 | | 0.0051 | | **0.0025** | | -0.0197 | | 0.0050 | | **4.60E-04** | |  | |
| Cingulate Lobe | -0.0707 | | 0.0252 | | **0.020** | | -0.0112 | | 0.0047 | | **0.021** | | -0.0133 | | 0.0046 | | **0.0051** | |  | |
|  |  | |  | |  | |  | |  | |  | |  | |  | |  | |  | |
|  |  | |  | |  | |  | |  | |  | |  | |  | |  | |  | |
| **Emotional Neglect** | | **GS (n=1,024)** | | | | | | **UKB (n=27,202)** | | | | | | **Mega-Analysis (n=28,226)** | | | | | |  |
| **Region** | | **Beta** | | **Std Err** | | **P(FDR)** | | **Beta** | | **Std Err** | | **P(FDR)** | | **Beta** | | **Std Err** | | **P(FDR)** | |  |
| Frontal Lobe | | -0.0256 | | 0.0260 | | 0.46 | | -0.0205 | | 0.0050 | | **6.28E-05** | | -0.0260 | | 0.0048 | | **3.1E-07** | |  |
| Temporal Lobe | | -0.0310 | | 0.0251 | | 0.46 | | -0.0260 | | 0.0049 | | **4.93E-07** | | -0.0208 | | 0.0049 | | **3.33E-05** | |  |
| Parietal Lobe | | -0.0229 | | 0.0254 | | 0.46 | | -0.0238 | | 0.0050 | | **6.28E-06** | | -0.0237 | | 0.0050 | | **4.5E-06** | |  |
| Occipital Lobe | | -0.0389 | | 0.0256 | | 0.46 | | -0.0147 | | 0.0051 | | **0.0039** | | -0.0155 | | 0.0050 | | **0.0020** | |  |
| Cingulate Lobe | | -0.0130 | | 0.0250 | | 0.60 | | -0.0159 | | 0.0047 | | **8.28E-04** | | -0.0158 | | 0.0046 | | **7.27E-04** | |  |
|  | |  | |  | |  | |  | |  | |  | |  | |  | |  | |  |
|  | |  | |  | |  | |  | |  | |  | |  | |  | |  | |  |
| **Physical Neglect** | | **GS (n=1,024)** | | | | | | **UKB (n=27,202)** | | | | | | **Mega-Analysis (n=28,226)** | | | | | |  |
| **Region** | | **Beta** | | **Std Err** | | **P(FDR)** | | **Beta** | | **Std Err** | | **P(FDR)** | | **Beta** | | **Std Err** | | **P(FDR)** | |  |
| Frontal Lobe | | -0.0243 | | 0.0260 | | 0.44 | | -0.0309 | | 0.0050 | | **6.89E-10** | | -0.0348 | | 0.0048 | | **2.46E-12** | |  |
| Temporal Lobe | | -0.0237 | | 0.0251 | | 0.44 | | -0.0350 | | 0.0049 | | **3.99E-12** | | -0.0308 | | 0.0049 | | **3.72E-10** | |  |
| Parietal Lobe | | -0.0308 | | 0.0254 | | 0.44 | | -0.0344 | | 0.0051 | | **2.6E-11** | | -0.0344 | | 0.0050 | | **1.04E-11** | |  |
| Occipital Lobe | | -0.0330 | | 0.0257 | | 0.44 | | -0.0321 | | 0.0051 | | **5.97E-10** | | -0.0322 | | 0.0050 | | **2.18E-10** | |  |
| Cingulate Lobe | | 0.0163 | | 0.0251 | | 0.51 | | -0.0192 | | 0.0047 | | **4.14E-05** | | -0.0181 | | 0.0046 | | **8.31E-05** | |  |
|  | |  | |  | |  | |  | |  | |  | |  | |  | |  | |  |

| **Abuse Composite Score** | **GS (n=1,024)** | | | | | | **UKB (n=27,202)** | | | | | | **Mega-Analysis (n=28,226)** | | | | | |  | |
| --- | --- | --- | --- | --- | --- | --- | --- | --- | --- | --- | --- | --- | --- | --- | --- | --- | --- | --- | --- | --- |
| **Region** | **Beta** | | **Std Err** | | **P(FDR)** | | **Beta** | | **Std Err** | | **P(FDR)** | | **Beta** | | **Std Err** | | **P(FDR)** | |  | |
| Frontal Lobe | -0.0817 | | 0.0261 | | **0.0031** | | -0.0197 | | 0.0050 | | **1.01E-04** | | -0.0291 | | 0.0048 | | **4.18E-09** | |  | |
| Temporal Lobe | -0.0737 | | 0.0252 | | **0.0035** | | -0.0271 | | 0.0049 | | **8.46E-08** | | -0.0218 | | 0.0049 | | **1.08E-05** | |  | |
| Parietal Lobe | -0.0773 | | 0.0255 | | **0.0031** | | -0.0294 | | 0.0051 | | **3.76E-08** | | -0.0312 | | 0.0050 | | **1.9E-09** | |  | |
| Occipital Lobe | -0.0923 | | 0.0257 | | **0.0017** | | -0.0270 | | 0.0051 | | **2.33E-07** | | -0.0296 | | 0.0050 | | **6.71E-09** | |  | |
| Cingulate Lobe | -0.0778 | | 0.0251 | | **0.0031** | | -0.0143 | | 0.0047 | | **0.0023** | | -0.0167 | | 0.0046 | | **3.01E-04** | |  | |
|  |  | |  | |  | |  | |  | |  | |  | |  | |  | |  | |
|  |  | |  | |  | |  | |  | |  | |  | |  | |  | |  | |
| **Neglect Composite Score** | | **GS (n=1,024)** | | | | | | **UKB (n=27,202)** | | | | | | **Mega-Analysis (n=28,226)** | | | | | |  |
| **Region** | | **Beta** | | **Std Err** | | **P(FDR)** | | **Beta** | | **Std Err** | | **P(FDR)** | | **Beta** | | **Std Err** | | **P(FDR)** | |  |
| Frontal Lobe | | -0.0280 | | 0.0260 | | 0.35 | | -0.0307 | | 0.0050 | | **1.11E-09** | | -0.0365 | | 0.0048 | | **1.38E-13** | |  |
| Temporal Lobe | | -0.0317 | | 0.0251 | | 0.35 | | -0.0368 | | 0.0049 | | **2.43E-13** | | -0.0307 | | 0.0049 | | **4.88E-10** | |  |
| Parietal Lobe | | -0.0287 | | 0.0254 | | 0.35 | | -0.0349 | | 0.0051 | | **1.26E-11** | | -0.0347 | | 0.0050 | | **6.81E-12** | |  |
| Occipital Lobe | | -0.0411 | | 0.0256 | | 0.35 | | -0.0272 | | 0.0051 | | **1.23E-07** | | -0.0277 | | 0.0050 | | **4.08E-08** | |  |
| Cingulate Lobe | | -0.0030 | | 0.0250 | | 0.90 | | -0.0214 | | 0.0047 | | **4.98E-06** | | -0.0208 | | 0.0046 | | **6.17E-06** | |  |
|  | |  | |  | |  | |  | |  | |  | |  | |  | |  | |  |

Lobar Thickness

| **Emotional Abuse** | **GS (n=1,024)** | | | | | | **UKB (n=27,202)** | | | | | | **Mega-Analysis (n=28,226)** | | | | | |  | |
| --- | --- | --- | --- | --- | --- | --- | --- | --- | --- | --- | --- | --- | --- | --- | --- | --- | --- | --- | --- | --- |
| **Region** | **Beta** | | **Std Err** | | **P(FDR)** | | **Beta** | | **Std Err** | | **P(FDR)** | | **Beta** | | **Std Err** | | **P(FDR)** | |  | |
| Frontal Lobe | -0.0099 | | 0.0268 | | 0.89 | | -0.0136 | | 0.0053 | | **0.027** | | -0.0109 | | 0.0051 | | 0.056 | |  | |
| Temporal Lobe | 0.0030 | | 0.0266 | | 0.91 | | -0.0107 | | 0.0052 | | 0.069 | | -0.0134 | | 0.0052 | | **0.026** | |  | |
| Parietal Lobe | -0.0123 | | 0.0241 | | 0.89 | | -0.0139 | | 0.0054 | | **0.027** | | -0.0141 | | 0.0053 | | **0.026** | |  | |
| Occipital Lobe | 0.0246 | | 0.0266 | | 0.89 | | 0.0025 | | 0.0054 | | 0.65 | | 0.0030 | | 0.0053 | | 0.62 | |  | |
| Cingulate Lobe | 0.0206 | | 0.0262 | | 0.89 | | -0.0031 | | 0.0050 | | 0.65 | | -0.0024 | | 0.0049 | | 0.62 | |  | |
|  |  | |  | |  | |  | |  | |  | |  | |  | |  | |  | |
|  |  | |  | |  | |  | |  | |  | |  | |  | |  | |  | |
| **Physical Abuse** | **GS (n=1,024)** | | | | | | **UKB (n=27,202)** | | | | | | **Mega-Analysis (n=28,226)** | | | | | |  | |
| **Region** | **Beta** | | **Std Err** | | **P(FDR)** | | **Beta** | | **Std Err** | | **P(FDR)** | | **Beta** | | **Std Err** | | **P(FDR)** | |  | |
| Frontal Lobe | -0.0272 | | 0.0265 | | 0.51 | | -0.0059 | | 0.0053 | | 0.54 | | -0.0062 | | 0.0051 | | 0.51 | |  | |
| Temporal Lobe | 0.0111 | | 0.0263 | | 0.67 | | -0.0051 | | 0.0052 | | 0.54 | | -0.0054 | | 0.0052 | | 0.51 | |  | |
| Parietal Lobe | -0.0267 | | 0.0238 | | 0.51 | | -0.0090 | | 0.0054 | | 0.48 | | -0.0098 | | 0.0053 | | 0.31 | |  | |
| Occipital Lobe | 0.0300 | | 0.0263 | | 0.51 | | 0.0031 | | 0.0054 | | 0.70 | | 0.0033 | | 0.0053 | | 0.67 | |  | |
| Cingulate Lobe | 0.0146 | | 0.0259 | | 0.67 | | 0.0015 | | 0.0050 | | 0.77 | | 0.0018 | | 0.0049 | | 0.71 | |  | |
|  |  | |  | |  | |  | |  | |  | |  | |  | |  | |  | |
|  |  | |  | |  | |  | |  | |  | |  | |  | |  | |  | |
| **Sexual Abuse** | **GS (n=1,024)** | | | | | | **UKB (n=27,202)** | | | | | | **Mega-Analysis (n=28,226)** | | | | | |  | |
| **Region** | **Beta** | | **Std Err** | | **P(FDR)** | | **Beta** | | **Std Err** | | **P(FDR)** | | **Beta** | | **Std Err** | | **P(FDR)** | |  | |
| Frontal Lobe | -0.0068 | | 0.0268 | | 0.91 | | 0.0051 | | 0.0053 | | 0.56 | | -0.0008 | | 0.0051 | | 0.87 | |  | |
| Temporal Lobe | 0.0202 | | 0.0266 | | 0.75 | | -0.0005 | | 0.0052 | | 0.92 | | 0.0054 | | 0.0052 | | 0.50 | |  | |
| Parietal Lobe | 0.0027 | | 0.0241 | | 0.91 | | 0.0025 | | 0.0054 | | 0.80 | | 0.0024 | | 0.0053 | | 0.81 | |  | |
| Occipital Lobe | 0.0254 | | 0.0266 | | 0.75 | | 0.0088 | | 0.0054 | | 0.27 | | 0.0095 | | 0.0053 | | 0.19 | |  | |
| Cingulate Lobe | 0.0240 | | 0.0262 | | 0.75 | | 0.0093 | | 0.0050 | | 0.27 | | 0.0098 | | 0.0049 | | 0.19 | |  | |
|  |  | |  | |  | |  | |  | |  | |  | |  | |  | |  | |
|  |  | |  | |  | |  | |  | |  | |  | |  | |  | |  | |
| **Emotional Neglect** | | **GS (n=1,024)** | | | | | | **UKB (n=27,202)** | | | | | | **Mega-Analysis (n=28,226)** | | | | | |  |
| **Region** | | **Beta** | | **Std Err** | | **P(FDR)** | | **Beta** | | **Std Err** | | **P(FDR)** | | **Beta** | | **Std Err** | | **P(FDR)** | |  |
| Frontal Lobe | | -0.0339 | | 0.0265 | | 0.50 | | -0.0076 | | 0.0053 | | 0.38 | | -0.0031 | | 0.0051 | | 0.66 | |  |
| Temporal Lobe | | -0.0109 | | 0.0263 | | 0.71 | | -0.0016 | | 0.0052 | | 0.76 | | -0.0077 | | 0.0052 | | 0.23 | |  |
| Parietal Lobe | | -0.0457 | | 0.0238 | | 0.28 | | -0.0061 | | 0.0054 | | 0.43 | | -0.0078 | | 0.0052 | | 0.23 | |  |
| Occipital Lobe | | -0.0098 | | 0.0263 | | 0.71 | | 0.0095 | | 0.0054 | | 0.38 | | 0.0085 | | 0.0053 | | 0.23 | |  |
| Cingulate Lobe | | -0.0112 | | 0.0259 | | 0.71 | | 0.0028 | | 0.0050 | | 0.71 | | 0.0022 | | 0.0049 | | 0.66 | |  |
|  | |  | |  | |  | |  | |  | |  | |  | |  | |  | |  |
|  | |  | |  | |  | |  | |  | |  | |  | |  | |  | |  |
| **Physical Neglect** | | **GS (n=1,024)** | | | | | | **UKB (n=27,202)** | | | | | | **Mega-Analysis (n=28,226)** | | | | | |  |
| **Region** | | **Beta** | | **Std Err** | | **P(FDR)** | | **Beta** | | **Std Err** | | **P(FDR)** | | **Beta** | | **Std Err** | | **P(FDR)** | |  |
| Frontal Lobe | | -0.0140 | | 0.0265 | | 0.90 | | -0.0044 | | 0.0053 | | 0.58 | | -0.0088 | | 0.0051 | | 0.21 | |  |
| Temporal Lobe | | -0.0097 | | 0.0264 | | 0.90 | | -0.0080 | | 0.0052 | | 0.32 | | -0.0049 | | 0.0052 | | 0.58 | |  |
| Parietal Lobe | | -0.0241 | | 0.0239 | | 0.90 | | -0.0082 | | 0.0054 | | 0.32 | | -0.0094 | | 0.0053 | | 0.21 | |  |
| Occipital Lobe | | 0.0034 | | 0.0264 | | 0.90 | | 0.0030 | | 0.0054 | | 0.58 | | 0.0022 | | 0.0053 | | 0.68 | |  |
| Cingulate Lobe | | 0.0068 | | 0.0259 | | 0.90 | | 0.0033 | | 0.0050 | | 0.58 | | 0.0031 | | 0.0049 | | 0.67 | |  |
|  | |  | |  | |  | |  | |  | |  | |  | |  | |  | |  |

| **Abuse Composite Score** | **GS (n=1,024)** | | | | | | **UKB (n=27,202)** | | | | | | **Mega-Analysis (n=28,226)** | | | | | |  | |
| --- | --- | --- | --- | --- | --- | --- | --- | --- | --- | --- | --- | --- | --- | --- | --- | --- | --- | --- | --- | --- |
| **Region** | **Beta** | | **Std Err** | | **P(FDR)** | | **Beta** | | **Std Err** | | **P(FDR)** | | **Beta** | | **Std Err** | | **P(FDR)** | |  | |
| Frontal Lobe | -0.0169 | | 0.0267 | | 0.58 | | -0.0197 | | 0.0050 | | **1.01E-04** | | -0.0088 | | 0.0051 | | 0.22 | |  | |
| Temporal Lobe | 0.0149 | | 0.0265 | | 0.58 | | -0.0271 | | 0.0049 | | **8.46E-08** | | -0.0076 | | 0.0052 | | 0.24 | |  | |
| Parietal Lobe | -0.0132 | | 0.0240 | | 0.58 | | -0.0294 | | 0.0051 | | **3.76E-08** | | -0.0110 | | 0.0053 | | 0.19 | |  | |
| Occipital Lobe | 0.0337 | | 0.0265 | | 0.58 | | -0.0270 | | 0.0051 | | **2.33E-07** | | 0.0067 | | 0.0053 | | 0.26 | |  | |
| Cingulate Lobe | 0.0259 | | 0.0261 | | 0.58 | | -0.0143 | | 0.0047 | | **0.0023** | | 0.0032 | | 0.0049 | | 0.52 | |  | |
|  |  | |  | |  | |  | |  | |  | |  | |  | |  | |  | |
|  |  | |  | |  | |  | |  | |  | |  | |  | |  | |  | |
| **Neglect Composite Score** | | **GS (n=1,024)** | | | | | | **UKB (n=27,202)** | | | | | | **Mega-Analysis (n=28,226)** | | | | | |  |
| **Region** | | **Beta** | | **Std Err** | | **P(FDR)** | | **Beta** | | **Std Err** | | **P(FDR)** | | **Beta** | | **Std Err** | | **P(FDR)** | |  |
| Frontal Lobe | | -0.0300 | | 0.0265 | | 0.64 | | -0.0307 | | 0.0050 | | **1.11E-09** | | -0.0067 | | 0.0051 | | 0.24 | |  |
| Temporal Lobe | | -0.0116 | | 0.0263 | | 0.83 | | -0.0368 | | 0.0049 | | **2.43E-13** | | -0.0080 | | 0.0052 | | 0.24 | |  |
| Parietal Lobe | | -0.0425 | | 0.0238 | | 0.37 | | -0.0349 | | 0.0051 | | **1.26E-11** | | -0.0104 | | 0.0052 | | 0.24 | |  |
| Occipital Lobe | | -0.0058 | | 0.0263 | | 0.83 | | -0.0272 | | 0.0051 | | **1.23E-07** | | 0.0073 | | 0.0053 | | 0.24 | |  |
| Cingulate Lobe | | -0.0055 | | 0.0259 | | 0.83 | | -0.0214 | | 0.0047 | | **4.98E-06** | | 0.0031 | | 0.0049 | | 0.52 | |  |
|  | |  | |  | |  | |  | |  | |  | |  | |  | |  | |  |

Cortical Volume

| **Emotional Abuse** | **GS (n=1,024)** | | | | | | **UKB (n=27,202)** | | | | | | **Mega-Analysis (n=28,226)** | | | | | |  | |
| --- | --- | --- | --- | --- | --- | --- | --- | --- | --- | --- | --- | --- | --- | --- | --- | --- | --- | --- | --- | --- |
| **Region** | **Beta** | | **Std Err** | | **P(FDR)** | | **Beta** | | **Std Err** | | **P(FDR)** | | **Beta** | | **Std Err** | | **P(FDR)** | |  | |
| Banks STS | 0.0278 | | 0.0233 | | 0.98 | | -0.0090 | | 0.0044 | | 0.11 | | -0.0079 | | 0.0043 | | 0.16 | |  | |
| Caudal Ant Cingulate** | -0.0156 | | 0.0214 | | 0.98 | | -0.0011 | | 0.0043 | | 0.91 | | -0.0019 | | 0.0042 | | 0.80 | |  | |
| Caudal Mid Frontal | 0.0106 | | 0.0236 | | 0.98 | | 0.0020 | | 0.0044 | | 0.83 | | 0.0023 | | 0.0043 | | 0.75 | |  | |
| Cuneus | -0.0139 | | 0.0239 | | 0.98 | | -0.0036 | | 0.0050 | | 0.65 | | -0.0042 | | 0.0049 | | 0.59 | |  | |
| Entorhinal | -0.0053 | | 0.0227 | | 0.98 | | -0.0003 | | 0.0049 | | 0.99 | | -0.0012 | | 0.0048 | | 0.89 | |  | |
| Frontal Pole | -0.0014 | | 0.0241 | | 0.98 | | 0.0002 | | 0.0047 | | 0.99 | | 0.0005 | | 0.0047 | | 0.92 | |  | |
| Fusiform | -0.0156 | | 0.0224 | | 0.98 | | -0.0109 | | 0.0041 | | **0.040** | | -0.0112 | | 0.0041 | | **0.032** | |  | |
| Inf Parietal | -0.0056 | | 0.0223 | | 0.98 | | -0.0204 | | 0.0043 | | **5.47E-05** | | -0.0201 | | 0.0042 | | **5.44E-05** | |  | |
| Inf Temporal | 0.0043 | | 0.0218 | | 0.98 | | -0.0090 | | 0.0040 | | 0.096 | | -0.00847 | | 0.0040 | | 0.098 | |  | |
| Insula | -0.0095 | | 0.0208 | | 0.98 | | -0.0172 | | 0.0042 | | **6.83E-04** | | -0.0171 | | 0.0041 | | **5.33E-04** | |  | |
| Isthmus Cingulate | -0.0038 | | 0.0232 | | 0.98 | | 0.0035 | | 0.0043 | | 0.60 | | 0.003134 | | 0.0043 | | 0.64 | |  | |
| Lat Occipital | -0.0196 | | 0.0217 | | 0.98 | | -0.0073 | | 0.0043 | | 0.21 | | -0.0078 | | 0.0042 | | 0.16 | |  | |
| Lat Orbitofrontal | 0.0045 | | 0.0212 | | 0.98 | | -0.0092 | | 0.0041 | | 0.096 | | -0.0088 | | 0.0041 | | 0.097 | |  | |
| Lingual | 0.0088 | | 0.0243 | | 0.98 | | 0.0079 | | 0.0050 | | 0.23 | | 0.0074 | | 0.0049 | | 0.27 | |  | |
| Med Orbitofrontal | 0.0017 | | 0.0207 | | 0.98 | | -0.0084 | | 0.0040 | | 0.11 | | -0.0081 | | 0.0039 | | 0.11 | |  | |
| Middle Temporal | -0.0187 | | 0.0206 | | 0.98 | | -0.0106 | | 0.0040 | | **0.040** | | -0.0110 | | 0.0039 | | **0.032** | |  | |
| Paracentral | 0.0089 | | 0.0229 | | 0.98 | | -0.0016 | | 0.0045 | | 0.85 | | -0.0012 | | 0.0044 | | 0.89 | |  | |
| Parahippocampal | -0.0193 | | 0.0247 | | 0.98 | | -0.0096 | | 0.0050 | | 0.14 | | -0.0110 | | 0.0049 | | 0.093 | |  | |
| Pars Opercularis | 0.0231 | | 0.0230 | | 0.98 | | -0.0047 | | 0.0045 | | 0.46 | | -0.0036 | | 0.0044 | | 0.59 | |  | |
| Pars Orbitalis | -0.0038 | | 0.0218 | | 0.98 | | -0.0019 | | 0.0044 | | 0.83 | | -0.0017 | | 0.0044 | | 0.81 | |  | |
| Pars Triangularis | -0.0171 | | 0.0229 | | 0.98 | | -0.0073 | | 0.0045 | | 0.23 | | -0.0075 | | 0.0045 | | 0.20 | |  | |
| Pericalcarine | -0.0076 | | 0.0263 | | 0.98 | | -0.0024 | | 0.0054 | | 0.83 | | -0.0031 | | 0.0053 | | 0.74 | |  | |
| Postcentral | 0.0103 | | 0.0225 | | 0.98 | | -0.0043 | | 0.0043 | | 0.48 | | -0.0037 | | 0.0042 | | 0.59 | |  | |
| Posterior Cingulate | 0.0040 | | 0.0226 | | 0.98 | | 0.0065 | | 0.0043 | | 0.26 | | 0.0061 | | 0.0042 | | 0.29 | |  | |
| Precentral | -0.0102 | | 0.0227 | | 0.98 | | -0.0113 | | 0.0043 | | **0.040** | | -0.0114 | | 0.0042 | | **0.032** | |  | |
| Precuneus | -0.0225 | | 0.0216 | | 0.98 | | -0.0118 | | 0.0041 | | **0.037** | | -0.0123 | | 0.0041 | | **0.021** | |  | |
| Rostral Ant Cingulate | -0.0119 | | 0.0217 | | 0.98 | | -0.0001 | | 0.0042 | | 0.99 | | -0.0006 | | 0.0041 | | 0.92 | |  | |
| Rostral Mid Frontal | -0.0228 | | 0.0203 | | 0.98 | | -0.0083 | | 0.0039 | | 0.11 | | -0.0088 | | 0.0039 | | 0.091 | |  | |
| Sup Frontal | -0.0048 | | 0.0197 | | 0.98 | | 0.0008 | | 0.0038 | | 0.92 | | 0.0005 | | 0.0038 | | 0.92 | |  | |
| Sup Parietal | -0.0180 | | 0.0225 | | 0.98 | | -0.0049 | | 0.0044 | | 0.45 | | -0.0055 | | 0.0044 | | 0.37 | |  | |
| Sup Temporal | 0.0158 | | 0.0218 | | 0.98 | | -0.0051 | | 0.0042 | | 0.38 | | -0.0044 | | 0.0041 | | 0.47 | |  | |
| Supramarginal | 0.0007 | | 0.0217 | | 0.98 | | -0.0128 | | 0.0041 | | **0.020** | | -0.0123 | | 0.0040 | | **0.021** | |  | |
| Temporal Pole* | -0.0043 | | 0.0248 | | 0.98 | |  | |  | |  | |  | |  | |  | |  | |
| Transverse Temporal | 0.0027 | | 0.0250 | | 0.98 | | -0.0067 | | 0.0048 | | 0.29 | | -0.0065 | | 0.0047 | | 0.31 | |  | |
|  |  | |  | |  | |  | |  | |  | |  | |  | |  | |  | |
|  |  | |  | |  | |  | |  | |  | |  | |  | |  | |  | |
| **Physical Abuse** | **GS (n=1,024)** | | | | | | **UKB (n=27,202)** | | | | | | **Mega-Analysis (n=28,226)** | | | | | |  | |
| **Region** | **Beta** | | **Std Err** | | **P(FDR)** | | **Beta** | | **Std Err** | | **P(FDR)** | | **Beta** | | **Std Err** | | **P(FDR)** | |  | |
| Banks STS | 0.0057 | | 0.0231 | | 0.87 | | -0.0038 | | 0.0044 | | 0.58 | | -0.0037 | | 0.0043 | | 0.56 | |  | |
| Caudal Ant Cingulate** | -0.0180 | | 0.0211 | | 0.61 | | -0.0100 | | 0.0042 | | 0.080 | | -0.0104 | | 0.0042 | | **0.042** | |  | |
| Caudal Mid Frontal | -0.0020 | | 0.0234 | | 0.93 | | 0.0057 | | 0.0044 | | 0.37 | | 0.0053 | | 0.0043 | | 0.38 | |  | |
| Cuneus | -0.0376 | | 0.0236 | | 0.35 | | -0.0118 | | 0.0050 | | 0.080 | | -0.0133 | | 0.0049 | | **0.033** | |  | |
| Entorhinal | -0.0733 | | 0.0224 | | **0.037** | | -0.0110 | | 0.0049 | | 0.080 | | -0.0138 | | 0.0048 | | **0.023** | |  | |
| Frontal Pole | -0.0189 | | 0.0238 | | 0.61 | | -0.0003 | | 0.0047 | | 0.97 | | -0.0006 | | 0.0046 | | 0.93 | |  | |
| Fusiform | -0.0307 | | 0.0221 | | 0.40 | | -0.0045 | | 0.0041 | | 0.48 | | -0.0056 | | 0.0040 | | 0.34 | |  | |
| Inf Parietal | -0.0283 | | 0.0221 | | 0.40 | | -0.0171 | | 0.0042 | | **0.0019** | | -0.0179 | | 0.0042 | | **6.23E-04** | |  | |
| Inf Temporal | 0.0175 | | 0.0216 | | 0.61 | | -0.0053 | | 0.0040 | | 0.37 | | -0.0048 | | 0.0040 | | 0.38 | |  | |
| Insula | -0.0550 | | 0.0205 | | 0.091 | | -0.0068 | | 0.0042 | | 0.25 | | -0.0088 | | 0.0041 | | 0.079 | |  | |
| Isthmus Cingulate | -0.0121 | | 0.0229 | | 0.74 | | -0.0031 | | 0.0043 | | 0.68 | | -0.0038 | | 0.0043 | | 0.56 | |  | |
| Lat Occipital | -0.0203 | | 0.0214 | | 0.61 | | -0.0097 | | 0.0043 | | 0.080 | | -0.0104 | | 0.0042 | | **0.042** | |  | |
| Lat Orbitofrontal | -0.0105 | | 0.0209 | | 0.74 | | -0.0016 | | 0.0041 | | 0.86 | | -0.0021 | | 0.0040 | | 0.72 | |  | |
| Lingual | 0.0317 | | 0.0240 | | 0.40 | | -0.0029 | | 0.0050 | | 0.73 | | -0.0024 | | 0.0049 | | 0.72 | |  | |
| Med Orbitofrontal | -0.0393 | | 0.0205 | | 0.27 | | -0.0005 | | 0.0040 | | 0.95 | | -0.0018 | | 0.0039 | | 0.72 | |  | |
| Middle Temporal | -0.0174 | | 0.0203 | | 0.61 | | -0.0076 | | 0.0040 | | 0.14 | | -0.0084 | | 0.0039 | | 0.079 | |  | |
| Paracentral | -0.0058 | | 0.0226 | | 0.87 | | -0.0067 | | 0.0044 | | 0.29 | | -0.0066 | | 0.0044 | | 0.28 | |  | |
| Parahippocampal | -0.0405 | | 0.0244 | | 0.33 | | -0.0124 | | 0.0050 | | 0.080 | | -0.0140 | | 0.0049 | | **0.023** | |  | |
| Pars Opercularis | 0.0154 | | 0.0228 | | 0.66 | | -0.0046 | | 0.0045 | | 0.49 | | -0.0039 | | 0.0044 | | 0.56 | |  | |
| Pars Orbitalis | -0.0329 | | 0.0215 | | 0.36 | | -0.0050 | | 0.0044 | | 0.48 | | -0.0058 | | 0.0043 | | 0.34 | |  | |
| Pars Triangularis | -0.0318 | | 0.0226 | | 0.40 | | -0.0011 | | 0.0045 | | 0.94 | | -0.0022 | | 0.0044 | | 0.72 | |  | |
| Pericalcarine | -0.0124 | | 0.0260 | | 0.74 | | -0.0123 | | 0.0054 | | 0.080 | | -0.0129 | | 0.0053 | | **0.042** | |  | |
| Postcentral | -0.0254 | | 0.0222 | | 0.48 | | -0.0087 | | 0.0042 | | 0.11 | | -0.0095 | | 0.0042 | | 0.064 | |  | |
| Posterior Cingulate | -0.0150 | | 0.0223 | | 0.66 | | 0.0006 | | 0.0043 | | 0.95 | | -0.0004 | | 0.0042 | | 0.93 | |  | |
| Precentral | -0.0414 | | 0.0224 | | 0.28 | | -0.0143 | | 0.0043 | | **0.013** | | -0.0154 | | 0.0042 | | **0.0038** | |  | |
| Precuneus | -0.0536 | | 0.0213 | | 0.10 | | -0.0106 | | 0.0041 | | 0.080 | | -0.0125 | | 0.0041 | | **0.017** | |  | |
| Rostral Ant Cingulate | -0.0288 | | 0.0215 | | 0.40 | | -0.0043 | | 0.0042 | | 0.49 | | -0.0054 | | 0.0041 | | 0.34 | |  | |
| Rostral Mid Frontal | -0.0423 | | 0.0200 | | 0.20 | | 0.0009 | | 0.0039 | | 0.94 | | -0.0006 | | 0.0038 | | 0.93 | |  | |
| Sup Frontal | -0.0461 | | 0.0195 | | 0.12 | | -0.0001 | | 0.0038 | | 0.97 | | -0.0019 | | 0.0038 | | 0.72 | |  | |
| Sup Parietal | -0.0590 | | 0.0222 | | 0.091 | | -0.0123 | | 0.0044 | | 0.060 | | -0.0143 | | 0.0044 | | **0.011** | |  | |
| Sup Temporal | 0.0042 | | 0.0216 | | 0.87 | | -0.0018 | | 0.0041 | | 0.85 | | -0.0019 | | 0.0041 | | 0.72 | |  | |
| Supramarginal | -0.0365 | | 0.0214 | | 0.33 | | -0.0086 | | 0.0041 | | 0.11 | | -0.0099 | | 0.0040 | | **0.042** | |  | |
| Temporal Pole* | 0.0047 | | 0.0245 | | 0.87 | |  | |  | |  | |  | |  | |  | |  | |
| Transverse Temporal | -0.0205 | | 0.0247 | | 0.61 | | -0.0029 | | 0.0048 | | 0.73 | | -0.0039 | | 0.0047 | | 0.56 | |  | |
|  |  | |  | |  | |  | |  | |  | |  | |  | |  | |  | |
|  |  | |  | |  | |  | |  | |  | |  | |  | |  | |  | |
| **Sexual Abuse** | **GS (n=1,024)** | | | | | | **UKB (n=27,202)** | | | | | | **Mega-Analysis (n=28,226)** | | | | | |  | |
| **Region** | **Beta** | | **Std Err** | | **P(FDR)** | | **Beta** | | **Std Err** | | **P(FDR)** | | **Beta** | | **Std Err** | | **P(FDR)** | |  | |
| Banks STS | 0.0277 | | 0.0233 | | 0.95 | | -0.0036 | | 0.0044 | | 0.97 | | -0.0024 | | 0.0043 | | 0.96 | |  | |
| Caudal Ant Cingulate** | -0.0258 | | 0.0214 | | 0.95 | | -0.0010 | | 0.0043 | | 0.99 | | -0.0020 | | 0.0042 | | 0.96 | |  | |
| Caudal Mid Frontal | -0.0056 | | 0.0237 | | 0.95 | | 0.0032 | | 0.0044 | | 0.97 | | 0.0031 | | 0.0043 | | 0.96 | |  | |
| Cuneus | 0.0012 | | 0.0240 | | 0.96 | | -0.0092 | | 0.0050 | | 0.56 | | -0.0087 | | 0.0049 | | 0.65 | |  | |
| Entorhinal | -0.0110 | | 0.0228 | | 0.95 | | 0.0002 | | 0.0049 | | 0.99 | | -0.0005 | | 0.0048 | | 0.97 | |  | |
| Frontal Pole | -0.0215 | | 0.0241 | | 0.95 | | 0.0043 | | 0.0047 | | 0.97 | | 0.0035 | | 0.0047 | | 0.96 | |  | |
| Fusiform | 0.0058 | | 0.0224 | | 0.95 | | 0.0021 | | 0.0041 | | 0.99 | | 0.0026 | | 0.0041 | | 0.96 | |  | |
| Inf Parietal | -0.0095 | | 0.0224 | | 0.95 | | -0.0028 | | 0.0043 | | 0.97 | | -0.0028 | | 0.0042 | | 0.96 | |  | |
| Inf Temporal | -0.0029 | | 0.0219 | | 0.95 | | -0.0029 | | 0.0040 | | 0.97 | | -0.0025 | | 0.0040 | | 0.96 | |  | |
| Insula | 0.0058 | | 0.0208 | | 0.95 | | -0.0001 | | 0.0042 | | 0.99 | | 0.0002 | | 0.0041 | | 0.97 | |  | |
| Isthmus Cingulate | -0.0143 | | 0.0232 | | 0.95 | | -0.0028 | | 0.0043 | | 0.97 | | -0.0029 | | 0.0043 | | 0.96 | |  | |
| Lat Occipital | -0.0191 | | 0.0217 | | 0.95 | | 0.0042 | | 0.0043 | | 0.97 | | 0.0035 | | 0.0042 | | 0.96 | |  | |
| Lat Orbitofrontal | -0.0020 | | 0.0212 | | 0.95 | | 0.0042 | | 0.0041 | | 0.97 | | 0.0042 | | 0.0041 | | 0.96 | |  | |
| Lingual | -0.0096 | | 0.0243 | | 0.95 | | -0.0032 | | 0.0050 | | 0.97 | | -0.0033 | | 0.0049 | | 0.96 | |  | |
| Med Orbitofrontal | -0.0064 | | 0.0208 | | 0.95 | | 0.0021 | | 0.0040 | | 0.99 | | 0.0019 | | 0.0040 | | 0.96 | |  | |
| Middle Temporal | -0.0171 | | 0.0206 | | 0.95 | | -0.0082 | | 0.0040 | | 0.44 | | -0.0083 | | 0.0039 | | 0.39 | |  | |
| Paracentral | 0.0160 | | 0.0229 | | 0.95 | | -0.0003 | | 0.0045 | | 0.99 | | 0.0003 | | 0.0044 | | 0.97 | |  | |
| Parahippocampal | 0.0129 | | 0.0247 | | 0.95 | | -0.0050 | | 0.0050 | | 0.97 | | -0.0048 | | 0.0049 | | 0.96 | |  | |
| Pars Opercularis | 0.0164 | | 0.0231 | | 0.95 | | 0.0013 | | 0.0045 | | 0.99 | | 0.0021 | | 0.0044 | | 0.96 | |  | |
| Pars Orbitalis | -0.0308 | | 0.0218 | | 0.95 | | 0.0000 | | 0.0044 | | 0.99 | | -0.0010 | | 0.0044 | | 0.97 | |  | |
| Pars Triangularis | 0.0242 | | 0.0229 | | 0.95 | | 0.0004 | | 0.0045 | | 0.99 | | 0.0015 | | 0.0045 | | 0.97 | |  | |
| Pericalcarine | -0.0038 | | 0.0264 | | 0.95 | | -0.0193 | | 0.0054 | | **0.011** | | -0.0187 | | 0.0053 | | **0.013** | |  | |
| Postcentral | 0.0067 | | 0.0226 | | 0.95 | | 0.0096 | | 0.0043 | | 0.40 | | 0.0098 | | 0.0042 | | 0.33 | |  | |
| Posterior Cingulate | -0.0138 | | 0.0226 | | 0.95 | | 0.0002 | | 0.0043 | | 0.99 | | -0.0001 | | 0.0042 | | 0.97 | |  | |
| Precentral | -0.0204 | | 0.0228 | | 0.95 | | 0.0005 | | 0.0043 | | 0.99 | | -0.0002 | | 0.0042 | | 0.97 | |  | |
| Precuneus | 0.0037 | | 0.0216 | | 0.95 | | -0.0060 | | 0.0042 | | 0.71 | | -0.0053 | | 0.0041 | | 0.96 | |  | |
| Rostral Ant Cingulate | -0.0187 | | 0.0217 | | 0.95 | | -0.0029 | | 0.0042 | | 0.97 | | -0.0033 | | 0.0041 | | 0.96 | |  | |
| Rostral Mid Frontal | -0.0158 | | 0.0203 | | 0.95 | | 0.0012 | | 0.0039 | | 0.99 | | 0.0007 | | 0.0039 | | 0.97 | |  | |
| Sup Frontal | 0.0039 | | 0.0198 | | 0.95 | | 0.0061 | | 0.0038 | | 0.65 | | 0.0062 | | 0.0038 | | 0.65 | |  | |
| Sup Parietal | 0.0138 | | 0.0226 | | 0.95 | | -0.0015 | | 0.0044 | | 0.99 | | -0.0007 | | 0.0044 | | 0.97 | |  | |
| Sup Temporal | 0.0248 | | 0.0218 | | 0.95 | | -0.0011 | | 0.0042 | | 0.99 | | 0.0002 | | 0.0041 | | 0.97 | |  | |
| Supramarginal | 0.0206 | | 0.0217 | | 0.95 | | -0.0064 | | 0.0041 | | 0.65 | | -0.0050 | | 0.0040 | | 0.96 | |  | |
| Temporal Pole* | 0.0146 | | 0.0248 | | 0.95 | |  | |  | |  | |  | |  | |  | |  | |
| Transverse Temporal | 0.0235 | | 0.0250 | | 0.95 | | 0.0001 | | 0.0048 | | 0.99 | | 0.0013 | | 0.0047 | | 0.97 | |  | |
|  |  | |  | |  | |  | |  | |  | |  | |  | |  | |  | |
|  |  | |  | |  | |  | |  | |  | |  | |  | |  | |  | |
| **Emotional Neglect** | | **GS (n=1,024)** | | | | | | **UKB (n=27,202)** | | | | | | **Mega-Analysis (n=28,226)** | | | | | |  |
| **Region** | | **Beta** | | **Std Err** | | **P(FDR)** | | **Beta** | | **Std Err** | | **P(FDR)** | | **Beta** | | **Std Err** | | **P(FDR)** | |  |
| Banks STS | | -0.0241 | | 0.0230 | | 0.76 | | -0.0164 | | 0.0044 | | **0.0027** | | -0.0169 | | 0.0043 | | **0.0013** | |  |
| Caudal Ant Cingulate** | | -0.0209 | | 0.0210 | | 0.76 | | -0.0075 | | 0.0042 | | 0.21 | | -0.0080 | | 0.0041 | | 0.15 | |  |
| Caudal Mid Frontal | | 0.0067 | | 0.0233 | | 0.99 | | -0.0018 | | 0.0043 | | 0.77 | | -0.0016 | | 0.0043 | | 0.84 | |  |
| Cuneus | | -0.0486 | | 0.0236 | | 0.76 | | -0.0035 | | 0.0050 | | 0.66 | | -0.0053 | | 0.0049 | | 0.49 | |  |
| Entorhinal | | -0.0028 | | 0.0224 | | 0.99 | | 0.0043 | | 0.0049 | | 0.57 | | 0.0041 | | 0.0048 | | 0.54 | |  |
| Frontal Pole | | -0.0250 | | 0.0238 | | 0.76 | | -0.0019 | | 0.0047 | | 0.77 | | -0.0028 | | 0.0046 | | 0.66 | |  |
| Fusiform | | -0.0183 | | 0.0221 | | 0.81 | | -0.0045 | | 0.0041 | | 0.48 | | -0.0048 | | 0.0040 | | 0.46 | |  |
| Inf Parietal | | -0.0049 | | 0.0220 | | 0.99 | | -0.0176 | | 0.0042 | | **0.0011** | | -0.0172 | | 0.0042 | | **0.0011** | |  |
| Inf Temporal | | 0.0015 | | 0.0216 | | 0.99 | | -0.0089 | | 0.0040 | | 0.094 | | -0.0086 | | 0.0039 | | 0.11 | |  |
| Insula | | -0.0159 | | 0.0205 | | 0.83 | | 0.0001 | | 0.0042 | | 0.98 | | -0.0005 | | 0.0041 | | 0.92 | |  |
| Isthmus Cingulate | | -0.0132 | | 0.0229 | | 0.96 | | 0.0044 | | 0.0043 | | 0.48 | | 0.0038 | | 0.0043 | | 0.53 | |  |
| Lat Occipital | | -0.0206 | | 0.0214 | | 0.76 | | 0.0061 | | 0.0042 | | 0.31 | | 0.0049 | | 0.0042 | | 0.46 | |  |
| Lat Orbitofrontal | | 0.0286 | | 0.0209 | | 0.76 | | -0.0023 | | 0.0041 | | 0.72 | | -0.0012 | | 0.0040 | | 0.87 | |  |
| Lingual | | 0.0334 | | 0.0240 | | 0.76 | | 0.0037 | | 0.0050 | | 0.65 | | 0.0048 | | 0.0049 | | 0.51 | |  |
| Med Orbitofrontal | | -0.0059 | | 0.0205 | | 0.99 | | -0.0129 | | 0.0040 | | **0.010** | | -0.0126 | | 0.0039 | | **0.011** | |  |
| Middle Temporal | | -0.0282 | | 0.0203 | | 0.76 | | -0.0144 | | 0.0040 | | **0.0034** | | -0.0151 | | 0.0039 | | **0.0013** | |  |
| Paracentral | | -0.0002 | | 0.0226 | | 0.99 | | -0.0076 | | 0.0044 | | 0.21 | | -0.0073 | | 0.0043 | | 0.22 | |  |
| Parahippocampal | | -0.0038 | | 0.0244 | | 0.99 | | -0.0051 | | 0.0050 | | 0.48 | | -0.0051 | | 0.0049 | | 0.49 | |  |
| Pars Opercularis | | 0.0074 | | 0.0227 | | 0.99 | | -0.0012 | | 0.0045 | | 0.80 | | -0.0009 | | 0.0044 | | 0.92 | |  |
| Pars Orbitalis | | -0.0211 | | 0.0215 | | 0.76 | | 0.0014 | | 0.0044 | | 0.80 | | 0.0005 | | 0.0043 | | 0.92 | |  |
| Pars Triangularis | | -0.0054 | | 0.0226 | | 0.99 | | -0.0028 | | 0.0045 | | 0.70 | | -0.0029 | | 0.0044 | | 0.64 | |  |
| Pericalcarine | | -0.0221 | | 0.0260 | | 0.81 | | -0.0128 | | 0.0053 | | 0.079 | | -0.0131 | | 0.0052 | | 0.052 | |  |
| Postcentral | | -0.0162 | | 0.0222 | | 0.83 | | -0.0108 | | 0.0042 | | 0.058 | | -0.0113 | | 0.0042 | | **0.036** | |  |
| Posterior Cingulate | | 0.0258 | | 0.0223 | | 0.76 | | -0.0048 | | 0.0043 | | 0.48 | | -0.0039 | | 0.0042 | | 0.53 | |  |
| Precentral | | -0.0335 | | 0.0224 | | 0.76 | | -0.0095 | | 0.0043 | | 0.094 | | -0.0106 | | 0.0042 | | 0.052 | |  |
| Precuneus | | -0.0299 | | 0.0213 | | 0.76 | | -0.0070 | | 0.0041 | | 0.21 | | -0.0079 | | 0.0041 | | 0.15 | |  |
| Rostral Ant Cingulate | | -0.0040 | | 0.0214 | | 0.99 | | -0.0062 | | 0.0041 | | 0.30 | | -0.0062 | | 0.0041 | | 0.28 | |  |
| Rostral Mid Frontal | | -0.0217 | | 0.0200 | | 0.76 | | -0.0106 | | 0.0039 | | **0.043** | | -0.0111 | | 0.0038 | | **0.024** | |  |
| Sup Frontal | | -0.0097 | | 0.0195 | | 0.96 | | -0.0069 | | 0.0038 | | 0.21 | | -0.0071 | | 0.0037 | | 0.15 | |  |
| Sup Parietal | | -0.0305 | | 0.0222 | | 0.76 | | 0.0017 | | 0.0044 | | 0.77 | | 0.0004 | | 0.0043 | | 0.92 | |  |
| Sup Temporal | | -0.0009 | | 0.0215 | | 0.99 | | -0.0046 | | 0.0041 | | 0.48 | | -0.0045 | | 0.0041 | | 0.48 | |  |
| Supramarginal | | 0.0110 | | 0.0214 | | 0.96 | | -0.0089 | | 0.0041 | | 0.094 | | -0.0082 | | 0.0040 | | 0.14 | |  |
| Temporal Pole* | | 0.0069 | | 0.0244 | | 0.99 | |  | |  | |  | |  | |  | |  | |  |
| Transverse Temporal | | 0.0273 | | 0.0246 | | 0.76 | | 0.0026 | | 0.0048 | | 0.72 | | 0.0034 | | 0.0047 | | 0.61 | |  |
|  | |  | |  | |  | |  | |  | |  | |  | |  | |  | |  |
|  | |  | |  | |  | |  | |  | |  | |  | |  | |  | |  |
| **Physical Neglect** | | **GS (n=1,024)** | | | | | | **UKB (n=27,202)** | | | | | | **Mega-Analysis (n=28,226)** | | | | | |  |
| **Region** | | **Beta** | | **Std Err** | | **P(FDR)** | | **Beta** | | **Std Err** | | **P(FDR)** | | **Beta** | | **Std Err** | | **P(FDR)** | |  |
| Banks STS | | -0.0003 | | 0.0230 | | 0.99 | | -0.0170 | | 0.0044 | | **0.0034** | | -0.0170 | | 0.0043 | | 0.0027 | |  |
| Caudal Ant Cingulate** | | -0.0055 | | 0.0211 | | 0.93 | | 0.0006 | | 0.0042 | | 0.91 | | 0.0001 | | 0.0042 | | 0.98 | |  |
| Caudal Mid Frontal | | 0.0308 | | 0.0233 | | 0.75 | | 0.0067 | | 0.0043 | | 0.23 | | 0.0071 | | 0.0043 | | 0.21 | |  |
| Cuneus | | -0.0324 | | 0.0236 | | 0.75 | | -0.0071 | | 0.0050 | | 0.27 | | -0.0084 | | 0.0049 | | 0.21 | |  |
| Entorhinal | | -0.0101 | | 0.0225 | | 0.91 | | -0.0009 | | 0.0049 | | 0.91 | | -0.0016 | | 0.0048 | | 0.87 | |  |
| Frontal Pole | | -0.0092 | | 0.0238 | | 0.91 | | 0.0074 | | 0.0047 | | 0.23 | | 0.0068 | | 0.0046 | | 0.25 | |  |
| Fusiform | | -0.0257 | | 0.0221 | | 0.75 | | -0.0032 | | 0.0041 | | 0.66 | | -0.0043 | | 0.0040 | | 0.43 | |  |
| Inf Parietal | | -0.0323 | | 0.0220 | | 0.75 | | -0.0105 | | 0.0042 | | 0.086 | | -0.0119 | | 0.0042 | | **0.035** | |  |
| Inf Temporal | | 0.0209 | | 0.0216 | | 0.75 | | -0.0069 | | 0.0040 | | 0.21 | | -0.0064 | | 0.0040 | | 0.21 | |  |
| Insula | | -0.0009 | | 0.0205 | | 0.99 | | -0.0095 | | 0.0042 | | 0.086 | | -0.0096 | | 0.0041 | | 0.070 | |  |
| Isthmus Cingulate | | 0.0319 | | 0.0229 | | 0.75 | | 0.0032 | | 0.0043 | | 0.66 | | 0.0037 | | 0.0043 | | 0.56 | |  |
| Lat Occipital | | -0.0156 | | 0.0214 | | 0.84 | | -0.0091 | | 0.0043 | | 0.10 | | -0.0098 | | 0.0042 | | 0.070 | |  |
| Lat Orbitofrontal | | 0.0330 | | 0.0209 | | 0.75 | | -0.0115 | | 0.0041 | | **0.042** | | -0.0103 | | 0.0040 | | 0.058 | |  |
| Lingual | | 0.0391 | | 0.0240 | | 0.75 | | -0.0022 | | 0.0050 | | 0.80 | | -0.0013 | | 0.0049 | | 0.89 | |  |
| Med Orbitofrontal | | -0.0153 | | 0.0205 | | 0.84 | | -0.0073 | | 0.0040 | | 0.19 | | -0.0077 | | 0.0039 | | 0.13 | |  |
| Middle Temporal | | -0.0106 | | 0.0203 | | 0.91 | | -0.0136 | | 0.0040 | | **0.011** | | -0.0141 | | 0.0039 | | **0.0046** | |  |
| Paracentral | | -0.0016 | | 0.0226 | | 0.99 | | -0.0029 | | 0.0044 | | 0.66 | | -0.0031 | | 0.0044 | | 0.65 | |  |
| Parahippocampal | | -0.0285 | | 0.0244 | | 0.75 | | -0.0041 | | 0.0050 | | 0.65 | | -0.0054 | | 0.0049 | | 0.42 | |  |
| Pars Opercularis | | 0.0228 | | 0.0227 | | 0.75 | | 0.0005 | | 0.0045 | | 0.91 | | 0.0011 | | 0.0044 | | 0.89 | |  |
| Pars Orbitalis | | -0.0076 | | 0.0215 | | 0.91 | | -0.0100 | | 0.0044 | | 0.086 | | -0.0100 | | 0.0043 | | 0.070 | |  |
| Pars Triangularis | | 0.0083 | | 0.0226 | | 0.91 | | -0.0073 | | 0.0045 | | 0.22 | | -0.0070 | | 0.0044 | | 0.21 | |  |
| Pericalcarine | | 0.0076 | | 0.0260 | | 0.93 | | -0.0089 | | 0.0054 | | 0.21 | | -0.0087 | | 0.0053 | | 0.21 | |  |
| Postcentral | | 0.0101 | | 0.0222 | | 0.91 | | -0.0027 | | 0.0042 | | 0.66 | | -0.0029 | | 0.0042 | | 0.65 | |  |
| Posterior Cingulate | | 0.0456 | | 0.0223 | | 0.75 | | -0.0006 | | 0.0043 | | 0.91 | | 0.0005 | | 0.0042 | | 0.94 | |  |
| Precentral | | -0.0228 | | 0.0224 | | 0.75 | | -0.0049 | | 0.0043 | | 0.41 | | -0.0060 | | 0.0042 | | 0.25 | |  |
| Precuneus | | -0.0268 | | 0.0213 | | 0.75 | | -0.0132 | | 0.0041 | | **0.016** | | -0.0143 | | 0.0041 | | **0.0046** | |  |
| Rostral Ant Cingulate | | 0.0199 | | 0.0214 | | 0.75 | | -0.0028 | | 0.0042 | | 0.66 | | -0.0025 | | 0.0041 | | 0.69 | |  |
| Rostral Mid Frontal | | -0.0120 | | 0.0200 | | 0.91 | | -0.0090 | | 0.0039 | | 0.086 | | -0.0095 | | 0.0038 | | 0.063 | |  |
| Sup Frontal | | -0.0011 | | 0.0195 | | 0.99 | | -0.0009 | | 0.0038 | | 0.91 | | -0.0013 | | 0.0038 | | 0.87 | |  |
| Sup Parietal | | -0.0343 | | 0.0222 | | 0.75 | | -0.0106 | | 0.0044 | | 0.086 | | -0.0121 | | 0.0044 | | **0.035** | |  |
| Sup Temporal | | 0.0156 | | 0.0215 | | 0.84 | | -0.0069 | | 0.0041 | | 0.21 | | -0.0066 | | 0.0041 | | 0.21 | |  |
| Supramarginal | | 0.0107 | | 0.0214 | | 0.91 | | -0.0083 | | 0.0041 | | 0.13 | | -0.0081 | | 0.0040 | | 0.13 | |  |
| Temporal Pole* | | -0.0057 | | 0.0244 | | 0.93 | |  | |  | |  | |  | |  | |  | |  |
| Transverse Temporal | | 0.0228 | | 0.0246 | | 0.75 | | -0.0009 | | 0.0048 | | 0.91 | | -0.0005 | | 0.0047 | | 0.94 | |  |
|  | |  | |  | |  | |  | |  | |  | |  | |  | |  | |  |

| **Abuse Composite Score** | **GS (n=1,024)** | | | | | | **UKB (n=27,202)** | | | | | | **Mega-Analysis (n=28,226)** | | | | | |  | |
| --- | --- | --- | --- | --- | --- | --- | --- | --- | --- | --- | --- | --- | --- | --- | --- | --- | --- | --- | --- | --- |
| **Region** | **Beta** | | **Std Err** | | **P(FDR)** | | **Beta** | | **Std Err** | | **P(FDR)** | | **Beta** | | **Std Err** | | **P(FDR)** | |  | |
| Banks STS | 0.0283 | | 0.0233 | | 0.83 | | -0.0078 | | 0.0044 | | 0.21 | | -0.0067 | | 0.0043 | | 0.27 | |  | |
| Caudal Ant Cingulate** | -0.0258 | | 0.0213 | | 0.83 | | -0.0057 | | 0.0043 | | 0.39 | | -0.0067 | | 0.0042 | | 0.25 | |  | |
| Caudal Mid Frontal | 0.0015 | | 0.0236 | | 0.98 | | 0.0050 | | 0.0044 | | 0.49 | | 0.0049 | | 0.0043 | | 0.47 | |  | |
| Cuneus | -0.0184 | | 0.0239 | | 0.83 | | -0.0109 | | 0.0050 | | 0.11 | | -0.0114 | | 0.0049 | | 0.075 | |  | |
| Entorhinal | -0.0323 | | 0.0227 | | 0.83 | | -0.0054 | | 0.0049 | | 0.49 | | -0.0071 | | 0.0048 | | 0.29 | |  | |
| Frontal Pole | -0.0175 | | 0.0241 | | 0.83 | | 0.0015 | | 0.0048 | | 0.78 | | 0.0013 | | 0.0047 | | 0.80 | |  | |
| Fusiform | -0.0147 | | 0.0224 | | 0.83 | | -0.0071 | | 0.0041 | | 0.22 | | -0.0073 | | 0.0041 | | 0.18 | |  | |
| Inf Parietal | -0.0167 | | 0.0223 | | 0.83 | | -0.0199 | | 0.0043 | | **9.94E-05** | | -0.01996 | | 0.0042 | | **6.34E-05** | |  | |
| Inf Temporal | 0.0064 | | 0.0218 | | 0.92 | | -0.0083 | | 0.0040 | | 0.13 | | -0.0077 | | 0.0040 | | 0.16 | |  | |
| Insula | -0.0200 | | 0.0208 | | 0.83 | | -0.0123 | | 0.0042 | | **0.019** | | -0.0127 | | 0.0041 | | **0.011** | |  | |
| Isthmus Cingulate | -0.0128 | | 0.0232 | | 0.90 | | -0.0007 | | 0.0044 | | 0.88 | | -0.0012 | | 0.0043 | | 0.80 | |  | |
| Lat Occipital | -0.0252 | | 0.0217 | | 0.83 | | -0.0069 | | 0.0043 | | 0.25 | | -0.0077 | | 0.0042 | | 0.18 | |  | |
| Lat Orbitofrontal | -0.0024 | | 0.0212 | | 0.98 | | -0.0040 | | 0.0041 | | 0.49 | | -0.0040 | | 0.0041 | | 0.50 | |  | |
| Lingual | 0.0100 | | 0.0243 | | 0.92 | | 0.0016 | | 0.0050 | | 0.78 | | 0.0012 | | 0.0049 | | 0.80 | |  | |
| Med Orbitofrontal | -0.0154 | | 0.0207 | | 0.83 | | -0.0039 | | 0.0040 | | 0.49 | | -0.0042 | | 0.0040 | | 0.50 | |  | |
| Middle Temporal | -0.0229 | | 0.0206 | | 0.83 | | -0.0122 | | 0.0040 | | **0.019** | | -0.0127 | | 0.0039 | | **0.011** | |  | |
| Paracentral | 0.0099 | | 0.0229 | | 0.92 | | -0.0042 | | 0.0045 | | 0.49 | | -0.0036 | | 0.0044 | | 0.53 | |  | |
| Parahippocampal | -0.0162 | | 0.0247 | | 0.83 | | -0.0128 | | 0.0050 | | **0.042** | | -0.0140 | | 0.0049 | | **0.021** | |  | |
| Pars Opercularis | 0.0239 | | 0.0230 | | 0.83 | | -0.0042 | | 0.0045 | | 0.49 | | -0.0030 | | 0.0044 | | 0.58 | |  | |
| Pars Orbitalis | -0.0278 | | 0.0218 | | 0.83 | | -0.0034 | | 0.0044 | | 0.53 | | -0.0040 | | 0.0044 | | 0.50 | |  | |
| Pars Triangularis | -0.0068 | | 0.0229 | | 0.92 | | -0.0042 | | 0.0045 | | 0.49 | | -0.0041 | | 0.0045 | | 0.50 | |  | |
| Pericalcarine | -0.0095 | | 0.0263 | | 0.92 | | -0.0143 | | 0.0054 | | **0.038** | | -0.0146 | | 0.0053 | | **0.023** | |  | |
| Postcentral | -0.0004 | | 0.0225 | | 0.98 | | -0.0029 | | 0.0043 | | 0.55 | | -0.0027 | | 0.0042 | | 0.59 | |  | |
| Posterior Cingulate | -0.0099 | | 0.0226 | | 0.92 | | 0.0038 | | 0.0043 | | 0.49 | | 0.0030 | | 0.0042 | | 0.58 | |  | |
| Precentral | -0.0285 | | 0.0227 | | 0.83 | | -0.0126 | | 0.0043 | | **0.019** | | -0.0133 | | 0.0042 | | **0.011** | |  | |
| Precuneus | -0.0266 | | 0.0216 | | 0.83 | | -0.0135 | | 0.0042 | | **0.015** | | -0.0140 | | 0.0041 | | **0.0097** | |  | |
| Rostral Ant Cingulate | -0.0243 | | 0.0217 | | 0.83 | | -0.0032 | | 0.0042 | | 0.53 | | -0.0040 | | 0.0041 | | 0.50 | |  | |
| Rostral Mid Frontal | -0.0324 | | 0.0203 | | 0.83 | | -0.0035 | | 0.0039 | | 0.49 | | -0.0044 | | 0.0039 | | 0.47 | |  | |
| Sup Frontal | -0.0157 | | 0.0197 | | 0.83 | | 0.0026 | | 0.0038 | | 0.55 | | 0.0019 | | 0.0038 | | 0.67 | |  | |
| Sup Parietal | -0.0214 | | 0.0225 | | 0.83 | | -0.0091 | | 0.0045 | | 0.13 | | -0.0095 | | 0.0044 | | 0.096 | |  | |
| Sup Temporal | 0.0208 | | 0.0218 | | 0.83 | | -0.0039 | | 0.0042 | | 0.49 | | -0.0031 | | 0.0041 | | 0.58 | |  | |
| Supramarginal | -0.0019 | | 0.0217 | | 0.98 | | -0.0132 | | 0.0041 | | **0.015** | | -0.0127 | | 0.0040 | | **0.011** | |  | |
| Temporal Pole* | 0.0067 | | 0.0248 | | 0.92 | |  | |  | |  | |  | |  | |  | |  | |
| Transverse Temporal | 0.0059 | | 0.0250 | | 0.92 | | -0.0049 | | 0.0048 | | 0.49 | | -0.0045 | | 0.0047 | | 0.50 | |  | |
|  |  | |  | |  | |  | |  | |  | |  | |  | |  | |  | |
|  | |  | |  | |  | |  | |  | |  | |  | |  | |  | |  |
| **Neglect Composite Score** | | **GS (n=1,024)** | | | | | | **UKB (n=27,202)** | | | | | | **Mega-Analysis (n=28,226)** | | | | | |  |
| **Region** | | **Beta** | | **Std Err** | | **P(FDR)** | | **Beta** | | **Std Err** | | **P(FDR)** | | **Beta** | | **Std Err** | | **P(FDR)** | |  |
| Banks STS | | -0.0176 | | 0.0230 | | 0.87 | | -0.0206 | | 0.0044 | | **8.06E-05** | | -0.02087 | | 0.0043 | | **3.93E-05** | |  |
| Caudal Ant Cingulate** | | -0.0172 | | 0.0211 | | 0.87 | | -0.0050 | | 0.0042 | | 0.37 | | -0.0056 | | 0.0041 | | 0.29 | |  |
| Caudal Mid Frontal | | 0.0169 | | 0.0233 | | 0.87 | | 0.0022 | | 0.0043 | | 0.70 | | 0.0025 | | 0.0043 | | 0.69 | |  |
| Cuneus | | -0.0478 | | 0.0236 | | 0.67 | | -0.0062 | | 0.0050 | | 0.37 | | -0.0080 | | 0.0049 | | 0.26 | |  |
| Entorhinal | | -0.0060 | | 0.0224 | | 0.93 | | 0.0026 | | 0.0049 | | 0.70 | | 0.0022 | | 0.0048 | | 0.72 | |  |
| Frontal Pole | | -0.0217 | | 0.0238 | | 0.87 | | 0.0025 | | 0.0047 | | 0.70 | | 0.0015 | | 0.0046 | | 0.77 | |  |
| Fusiform | | -0.0233 | | 0.0221 | | 0.87 | | -0.0048 | | 0.0041 | | 0.37 | | -0.0056 | | 0.0040 | | 0.29 | |  |
| Inf Parietal | | -0.0162 | | 0.0220 | | 0.87 | | -0.0180 | | 0.0042 | | **2.35E-04** | | -0.0183 | | 0.0042 | | **1.22E-04** | |  |
| Inf Temporal | | 0.0093 | | 0.0216 | | 0.91 | | -0.0099 | | 0.0040 | | **0.050** | | -0.0095 | | 0.0039 | | 0.053 | |  |
| Insula | | -0.0118 | | 0.0205 | | 0.87 | | -0.0049 | | 0.0042 | | 0.37 | | -0.0053 | | 0.0041 | | 0.29 | |  |
| Isthmus Cingulate | | 0.0030 | | 0.0229 | | 0.97 | | 0.0048 | | 0.0043 | | 0.38 | | 0.0045 | | 0.0043 | | 0.38 | |  |
| Lat Occipital | | -0.0210 | | 0.0214 | | 0.87 | | -0.0004 | | 0.0042 | | 0.92 | | -0.0015 | | 0.0042 | | 0.76 | |  |
| Lat Orbitofrontal | | 0.0337 | | 0.0209 | | 0.67 | | -0.0077 | | 0.0041 | | 0.17 | | -0.0064 | | 0.0040 | | 0.26 | |  |
| Lingual | | 0.0395 | | 0.0239 | | 0.67 | | 0.0015 | | 0.0050 | | 0.82 | | 0.0026 | | 0.0049 | | 0.70 | |  |
| Med Orbitofrontal | | -0.0103 | | 0.0205 | | 0.87 | | -0.0130 | | 0.0040 | | **0.0098** | | -0.0130 | | 0.0039 | | **0.006** | |  |
| Middle Temporal | | -0.0246 | | 0.0203 | | 0.87 | | -0.0174 | | 0.0040 | | **2.30E-04** | | -0.01801 | | 0.0039 | | **7.38E-05** | |  |
| Paracentral | | -0.0008 | | 0.0226 | | 0.98 | | -0.0069 | | 0.0044 | | 0.28 | | -0.0068 | | 0.0044 | | 0.26 | |  |
| Parahippocampal | | -0.0139 | | 0.0244 | | 0.87 | | -0.0058 | | 0.0050 | | 0.37 | | -0.0063 | | 0.0049 | | 0.29 | |  |
| Pars Opercularis | | 0.0143 | | 0.0227 | | 0.87 | | -0.0006 | | 0.0045 | | 0.92 | | -0.0002 | | 0.0044 | | 0.96 | |  |
| Pars Orbitalis | | -0.0183 | | 0.0215 | | 0.87 | | -0.0042 | | 0.0044 | | 0.45 | | -0.0048 | | 0.0043 | | 0.37 | |  |
| Pars Triangularis | | -0.0007 | | 0.0226 | | 0.98 | | -0.0058 | | 0.0045 | | 0.37 | | -0.0058 | | 0.0044 | | 0.29 | |  |
| Pericalcarine | | -0.0130 | | 0.0260 | | 0.87 | | -0.0138 | | 0.0054 | | **0.042** | | -0.0138 | | 0.0052 | | **0.039** | |  |
| Postcentral | | -0.0078 | | 0.0222 | | 0.91 | | -0.0091 | | 0.0042 | | 0.094 | | -0.0096 | | 0.0042 | | 0.064 | |  |
| Posterior Cingulate | | 0.0366 | | 0.0223 | | 0.67 | | -0.0037 | | 0.0043 | | 0.48 | | -0.0027 | | 0.0042 | | 0.67 | |  |
| Precentral | | -0.0332 | | 0.0224 | | 0.67 | | -0.0094 | | 0.0043 | | 0.092 | | -0.0106 | | 0.0042 | | **0.045** | |  |
| Precuneus | | -0.0322 | | 0.0213 | | 0.67 | | -0.0119 | | 0.0041 | | **0.022** | | -0.0130 | | 0.0041 | | **0.0077** | |  |
| Rostral Ant Cingulate | | 0.0049 | | 0.0214 | | 0.93 | | -0.0059 | | 0.0042 | | 0.33 | | -0.0058 | | 0.0041 | | 0.29 | |  |
| Rostral Mid Frontal | | -0.0204 | | 0.0200 | | 0.87 | | -0.0123 | | 0.0039 | | **0.011** | | -0.0128 | | 0.0038 | | **0.0062** | |  |
| Sup Frontal | | -0.0074 | | 0.0195 | | 0.91 | | -0.0054 | | 0.0038 | | 0.33 | | -0.0057 | | 0.0038 | | 0.26 | |  |
| Sup Parietal | | -0.0355 | | 0.0222 | | 0.67 | | -0.0043 | | 0.0044 | | 0.45 | | -0.0058 | | 0.0043 | | 0.29 | |  |
| Sup Temporal | | 0.0055 | | 0.0215 | | 0.93 | | -0.0069 | | 0.0041 | | 0.24 | | -0.0067 | | 0.0041 | | 0.26 | |  |
| Supramarginal | | 0.0122 | | 0.0214 | | 0.87 | | -0.0107 | | 0.0041 | | **0.042** | | -0.0101 | | 0.0040 | | **0.045** | |  |
| Temporal Pole* | | 0.0028 | | 0.0244 | | 0.97 | |  | |  | |  | |  | |  | |  | |  |
| Transverse Temporal | | 0.0287 | | 0.0246 | | 0.87 | | 0.0014 | | 0.0048 | | 0.82 | | 0.0021 | | 0.0047 | | 0.72 | |  |
|  | |  | |  | |  | |  | |  | |  | |  | |  | |  | |  |

Cortical Surface Area

| **Emotional Abuse** | **GS (n=1,024)** | | | **UKB (n=27,202)** | | | | | **Mega-Analysis (n=28,226)** | | | | |  | |  |
| --- | --- | --- | --- | --- | --- | --- | --- | --- | --- | --- | --- | --- | --- | --- | --- | --- |
| **Region** | **Beta** | **Std Err** | **P(FDR)** | **Beta** | **Std Err** | | **P(FDR)** | | **Beta** | **Std Err** | | **P(FDR)** | |  | |  |
| Banks STS | 0.0280 | 0.0235 | 0.97 | -0.0063 | 0.0044 | | 0.34 | | -0.0052 | 0.0043 | | 0.39 | |  | |  |
| Caudal Ant Cingulate** | -0.0086 | 0.0221 | 0.98 | 0.0020 | 0.0042 | | 0.79 | | 0.0016 | 0.0041 | | 0.77 | |  | |  |
| Caudal Mid Frontal | 0.0078 | 0.0241 | 0.98 | 0.0041 | 0.0043 | | 0.53 | | 0.0043 | 0.0043 | | 0.50 | |  | |  |
| Cuneus | -0.0408 | 0.0242 | 0.97 | -0.0054 | 0.0049 | | 0.44 | | -0.0068 | 0.0048 | | 0.32 | |  | |  |
| Entorhinal | 0.0127 | 0.0239 | 0.98 | -0.0006 | 0.0049 | | 0.92 | | -0.0006 | 0.0048 | | 0.90 | |  | |  |
| Frontal Pole | -0.0233 | 0.0233 | 0.97 | -0.0037 | 0.0042 | | 0.54 | | -0.0039 | 0.0041 | | 0.51 | |  | |  |
| Fusiform | -0.0214 | 0.0226 | 0.97 | -0.0123 | 0.0039 | | **0.028** | | -0.0125 | 0.0039 | | **0.022** | |  | |  |
| Inf Parietal | -0.0002 | 0.0229 | 0.99 | -0.0148 | 0.0043 | | **0.017** | | -0.0146 | 0.0042 | | **0.017** | |  | |  |
| Inf Temporal | 0.0015 | 0.0226 | 0.99 | -0.0067 | 0.0040 | | 0.25 | | -0.0065 | 0.0039 | | 0.25 | |  | |  |
| Insula | -0.0012 | 0.0213 | 0.99 | -0.0086 | 0.0040 | | 0.17 | | -0.0084 | 0.0039 | | 0.16 | |  | |  |
| Isthmus Cingulate | -0.0161 | 0.0225 | 0.97 | 0.0037 | 0.0041 | | 0.54 | | 0.0030 | 0.0041 | | 0.64 | |  | |  |
| Lat Occipital | -0.0268 | 0.0225 | 0.97 | -0.0101 | 0.0044 | | 0.17 | | -0.0108 | 0.0043 | | 0.097 | |  | |  |
| Lat Orbitofrontal | -0.0048 | 0.0232 | 0.99 | -0.0049 | 0.0040 | | 0.42 | | -0.0048 | 0.0040 | | 0.39 | |  | |  |
| Lingual | -0.0318 | 0.0247 | 0.97 | 0.0000 | 0.0050 | | 0.99 | | -0.0015 | 0.0049 | | 0.81 | |  | |  |
| Med Orbitofrontal | -0.0135 | 0.0217 | 0.97 | -0.0014 | 0.0036 | | 0.79 | | -0.0017 | 0.0035 | | 0.77 | |  | |  |
| Middle Temporal | -0.0207 | 0.0221 | 0.97 | -0.0079 | 0.0039 | | 0.18 | | -0.0084 | 0.0039 | | 0.16 | |  | |  |
| Paracentral | 0.0186 | 0.0231 | 0.97 | 0.0113 | 0.0042 | | 0.088 | | 0.0116 | 0.0042 | | 0.062 | |  | |  |
| Parahippocampal | -0.0293 | 0.0238 | 0.97 | -0.0077 | 0.0044 | | 0.24 | | -0.0087 | 0.0043 | | 0.16 | |  | |  |
| Pars Opercularis | 0.0197 | 0.0238 | 0.97 | 0.0011 | 0.0045 | | 0.89 | | 0.0019 | 0.0044 | | 0.77 | |  | |  |
| Pars Orbitalis | -0.0081 | 0.0229 | 0.98 | -0.0016 | 0.0042 | | 0.79 | | -0.0017 | 0.0042 | | 0.77 | |  | |  |
| Pars Triangularis | -0.0206 | 0.0245 | 0.97 | -0.0007 | 0.0045 | | 0.92 | | -0.0011 | 0.0045 | | 0.82 | |  | |  |
| Pericalcarine | -0.0494 | 0.0266 | 0.97 | -0.0083 | 0.0055 | | 0.33 | | -0.0101 | 0.0053 | | 0.16 | |  | |  |
| Postcentral | -0.0033 | 0.0229 | 0.99 | -0.0044 | 0.0039 | | 0.44 | | -0.0044 | 0.0038 | | 0.41 | |  | |  |
| Posterior Cingulate | -0.0014 | 0.0224 | 0.99 | 0.0081 | 0.0040 | | 0.18 | | 0.0077 | 0.0040 | | 0.16 | |  | |  |
| Precentral | -0.0163 | 0.0225 | 0.97 | -0.0029 | 0.0040 | | 0.65 | | -0.0034 | 0.0040 | | 0.57 | |  | |  |
| Precuneus | -0.0147 | 0.0223 | 0.97 | -0.0054 | 0.0042 | | 0.40 | | -0.0058 | 0.0041 | | 0.32 | |  | |  |
| Rostral Ant Cingulate | -0.0040 | 0.0222 | 0.99 | 0.0079 | 0.0040 | | 0.18 | | 0.0075 | 0.0040 | | 0.16 | |  | |  |
| Rostral Mid Frontal | -0.0242 | 0.0223 | 0.97 | -0.0068 | 0.0039 | | 0.24 | | -0.0075 | 0.0038 | | 0.16 | |  | |  |
| Sup Frontal | 0.0085 | 0.0213 | 0.98 | 0.0053 | 0.0037 | | 0.34 | | 0.0053 | 0.0037 | | 0.32 | |  | |  |
| Sup Parietal | -0.0296 | 0.0239 | 0.97 | -0.0019 | 0.0044 | | 0.79 | | -0.0030 | 0.0044 | | 0.64 | |  | |  |
| Sup Temporal | 0.0021 | 0.0219 | 0.99 | -0.0025 | 0.0039 | | 0.68 | | -0.0023 | 0.0038 | | 0.69 | |  | |  |
| Supramarginal | 0.0071 | 0.0224 | 0.98 | -0.0090 | 0.0041 | | 0.17 | | -0.0083 | 0.0040 | | 0.16 | |  | |  |
| Temporal Pole* | 0.0088 | 0.0229 | 0.98 |  |  | |  | |  |  | |  | |  | |  |
| Transverse Temporal | -0.0155 | 0.0253 | 0.97 | -0.0056 | 0.0046 | | 0.42 | | -0.0056 | 0.0046 | | 0.39 | |  | |  |
|  |  |  |  |  |  | |  | |  |  | |  | |  | |  |
|  |  |  |  |  |  | |  | |  |  | |  | |  | |  |
| **Physical Abuse** | **GS (n=1,024)** | | | **UKB (n=27,202)** | | | | | **Mega-Analysis (n=28,226)** | | | | |  | |  |
| **Region** | **Beta** | **Std Err** | **P(FDR)** | **Beta** | **Std Err** | | **P(FDR)** | | **Beta** | **Std Err** | | **P(FDR)** | |  | |  |
| Banks STS | 0.0028 | 0.0232 | 0.93 | -0.0015 | 0.0043 | | 0.87 | | -0.0014 | 0.0043 | | 0.78 | |  | |  |
| Caudal Ant Cingulate** | -0.0147 | 0.0219 | 0.63 | -0.0073 | 0.0042 | | 0.19 | | -0.0078 | 0.0041 | | 0.12 | |  | |  |
| Caudal Mid Frontal | -0.0079 | 0.0238 | 0.84 | 0.0054 | 0.0043 | | 0.36 | | 0.0047 | 0.0043 | | 0.43 | |  | |  |
| Cuneus | -0.0650 | 0.0239 | 0.075 | -0.0157 | 0.0049 | | **0.021** | | -0.0178 | 0.0048 | | **0.0034** | |  | |  |
| Entorhinal | -0.0507 | 0.0235 | 0.18 | -0.0083 | 0.0049 | | 0.20 | | -0.0103 | 0.0048 | | 0.081 | |  | |  |
| Frontal Pole | -0.0274 | 0.0231 | 0.36 | -0.0098 | 0.0042 | | 0.073 | | -0.0102 | 0.0041 | | **0.050** | |  | |  |
| Fusiform | -0.0465 | 0.0223 | 0.18 | -0.0098 | 0.0039 | | 0.054 | | -0.0113 | 0.0039 | | **0.017** | |  | |  |
| Inf Parietal | -0.0266 | 0.0226 | 0.36 | -0.0125 | 0.0043 | | **0.022** | | -0.0133 | 0.0042 | | **0.0079** | |  | |  |
| Inf Temporal | 0.0040 | 0.0223 | 0.91 | -0.0061 | 0.0040 | | 0.24 | | -0.0062 | 0.0039 | | 0.20 | |  | |  |
| Insula | -0.0586 | 0.0209 | 0.075 | -0.0012 | 0.0040 | | 0.87 | | -0.0033 | 0.0039 | | 0.57 | |  | |  |
| Isthmus Cingulate | -0.0315 | 0.0223 | 0.30 | -0.0061 | 0.0041 | | 0.25 | | -0.0073 | 0.0040 | | 0.14 | |  | |  |
| Lat Occipital | -0.0391 | 0.0222 | 0.21 | -0.0134 | 0.0043 | | **0.022** | | -0.0145 | 0.0043 | | **0.0055** | |  | |  |
| Lat Orbitofrontal | -0.0266 | 0.0229 | 0.36 | -0.0015 | 0.0040 | | 0.87 | | -0.0026 | 0.0039 | | 0.68 | |  | |  |
| Lingual | -0.0127 | 0.0244 | 0.73 | -0.0123 | 0.0049 | | 0.054 | | -0.0127 | 0.0049 | | **0.036** | |  | |  |
| Med Orbitofrontal | -0.0293 | 0.0214 | 0.31 | 0.0002 | 0.0035 | | 0.95 | | -0.0010 | 0.0035 | | 0.78 | |  | |  |
| Middle Temporal | -0.0283 | 0.0219 | 0.33 | -0.0065 | 0.0039 | | 0.20 | | -0.0077 | 0.0039 | | 0.10 | |  | |  |
| Paracentral | 0.0003 | 0.0228 | 0.99 | 0.0017 | 0.0042 | | 0.87 | | 0.0015 | 0.0042 | | 0.78 | |  | |  |
| Parahippocampal | -0.0391 | 0.0236 | 0.22 | -0.0080 | 0.0044 | | 0.19 | | -0.0093 | 0.0043 | | 0.081 | |  | |  |
| Pars Opercularis | 0.0044 | 0.0236 | 0.91 | -0.0028 | 0.0045 | | 0.72 | | -0.0027 | 0.0044 | | 0.69 | |  | |  |
| Pars Orbitalis | -0.0415 | 0.0226 | 0.21 | -0.0085 | 0.0042 | | 0.14 | | -0.0097 | 0.0041 | | 0.058 | |  | |  |
| Pars Triangularis | -0.0437 | 0.0242 | 0.21 | -0.0037 | 0.0045 | | 0.64 | | -0.0051 | 0.0044 | | 0.41 | |  | |  |
| Pericalcarine | -0.0569 | 0.0263 | 0.18 | -0.0176 | 0.0054 | | **0.021** | | -0.0193 | 0.0053 | | **0.0034** | |  | |  |
| Postcentral | -0.0398 | 0.0226 | 0.21 | -0.0111 | 0.0039 | | **0.023** | | -0.0123 | 0.0038 | | **0.0079** | |  | |  |
| Posterior Cingulate | -0.0250 | 0.0221 | 0.37 | -0.0027 | 0.0040 | | 0.72 | | -0.0037 | 0.0040 | | 0.52 | |  | |  |
| Precentral | -0.0432 | 0.0222 | 0.21 | -0.0080 | 0.0040 | | 0.14 | | -0.0093 | 0.0039 | | 0.058 | |  | |  |
| Precuneus | -0.0417 | 0.0220 | 0.21 | -0.0072 | 0.0041 | | 0.19 | | -0.0086 | 0.0041 | | 0.084 | |  | |  |
| Rostral Ant Cingulate | -0.0088 | 0.0220 | 0.81 | 0.0026 | 0.0040 | | 0.72 | | 0.0018 | 0.0039 | | 0.78 | |  | |  |
| Rostral Mid Frontal | -0.0463 | 0.0220 | 0.18 | 0.0005 | 0.0039 | | 0.95 | | -0.0014 | 0.0038 | | 0.78 | |  | |  |
| Sup Frontal | -0.0318 | 0.0211 | 0.26 | 0.0004 | 0.0037 | | 0.95 | | -0.0010 | 0.0037 | | 0.78 | |  | |  |
| Sup Parietal | -0.0771 | 0.0235 | **0.036** | -0.0131 | 0.0044 | | **0.022** | | -0.0157 | 0.0043 | | **0.0034** | |  | |  |
| Sup Temporal | -0.0196 | 0.0216 | 0.50 | -0.0023 | 0.0039 | | 0.74 | | -0.0031 | 0.0038 | | 0.57 | |  | |  |
| Supramarginal | -0.0362 | 0.0221 | 0.22 | -0.0056 | 0.0040 | | 0.28 | | -0.0069 | 0.0040 | | 0.15 | |  | |  |
| Temporal Pole* | 0.0154 | 0.0226 | 0.63 |  |  | |  | |  |  | |  | |  | |  |
| Transverse Temporal | -0.0412 | 0.0250 | 0.22 | -0.0007 | 0.0046 | | 0.95 | | -0.0019 | 0.0046 | | 0.78 | |  | |  |
|  |  |  |  |  |  | |  | |  |  | |  | |  | |  |
|  |  |  |  |  |  | |  | |  |  | |  | |  | |  |
| **Sexual Abuse** | **GS (n=1,024)** | | | **UKB (n=27,202)** | | | | | **Mega-Analysis (n=28,226)** | | | | |  | |  |
| **Region** | **Beta** | **Std Err** | **P(FDR)** | **Beta** | **Std Err** | | **P(FDR)** | | **Beta** | **Std Err** | | **P(FDR)** | |  | |  |
| Banks STS | -0.0015 | 0.0235 | 0.98 | -0.0030 | 0.0044 | | 0.93 | | -0.0028 | 0.0043 | | 0.88 | |  | |  |
| Caudal Ant Cingulate** | -0.0297 | 0.0221 | 0.94 | -0.0020 | 0.0042 | | 0.93 | | -0.0028 | 0.0041 | | 0.88 | |  | |  |
| Caudal Mid Frontal | -0.0088 | 0.0241 | 0.94 | -0.0013 | 0.0043 | | 0.93 | | -0.0012 | 0.0043 | | 0.89 | |  | |  |
| Cuneus | -0.0301 | 0.0242 | 0.94 | -0.0150 | 0.0049 | | **0.037** | | -0.0154 | 0.0048 | | **0.022** | |  | |  |
| Entorhinal | 0.0043 | 0.0239 | 0.94 | -0.0014 | 0.0049 | | 0.93 | | -0.0014 | 0.0048 | | 0.89 | |  | |  |
| Frontal Pole | -0.0092 | 0.0234 | 0.94 | -0.0025 | 0.0042 | | 0.93 | | -0.0022 | 0.0041 | | 0.88 | |  | |  |
| Fusiform | 0.0104 | 0.0227 | 0.94 | 0.0006 | 0.0039 | | 0.93 | | 0.0016 | 0.0039 | | 0.89 | |  | |  |
| Inf Parietal | -0.0150 | 0.0229 | 0.94 | -0.0017 | 0.0043 | | 0.93 | | -0.0020 | 0.0042 | | 0.88 | |  | |  |
| Inf Temporal | -0.0129 | 0.0226 | 0.94 | -0.0036 | 0.0040 | | 0.92 | | -0.0034 | 0.0039 | | 0.88 | |  | |  |
| Insula | 0.0148 | 0.0213 | 0.94 | 0.0006 | 0.0040 | | 0.93 | | 0.0011 | 0.0039 | | 0.89 | |  | |  |
| Isthmus Cingulate | -0.0187 | 0.0226 | 0.94 | -0.0063 | 0.0041 | | 0.47 | | -0.0065 | 0.0041 | | 0.50 | |  | |  |
| Lat Occipital | -0.0257 | 0.0225 | 0.94 | -0.0029 | 0.0044 | | 0.93 | | -0.0036 | 0.0043 | | 0.88 | |  | |  |
| Lat Orbitofrontal | -0.0081 | 0.0232 | 0.94 | 0.0052 | 0.0040 | | 0.64 | | 0.0051 | 0.0040 | | 0.65 | |  | |  |
| Lingual | -0.0175 | 0.0247 | 0.94 | -0.0134 | 0.0050 | | 0.078 | | -0.0134 | 0.0049 | | 0.065 | |  | |  |
| Med Orbitofrontal | -0.0048 | 0.0217 | 0.94 | 0.0017 | 0.0036 | | 0.93 | | 0.0018 | 0.0035 | | 0.88 | |  | |  |
| Middle Temporal | -0.0402 | 0.0221 | 0.94 | -0.0088 | 0.0039 | | 0.21 | | -0.0095 | 0.0039 | | 0.12 | |  | |  |
| Paracentral | 0.0147 | 0.0231 | 0.94 | -0.0004 | 0.0043 | | 0.93 | | 0.0005 | 0.0042 | | 0.97 | |  | |  |
| Parahippocampal | -0.0112 | 0.0239 | 0.94 | -0.0069 | 0.0044 | | 0.47 | | -0.0069 | 0.0043 | | 0.50 | |  | |  |
| Pars Opercularis | 0.0035 | 0.0239 | 0.94 | -0.0004 | 0.0045 | | 0.93 | | 0.0001 | 0.0044 | | 0.98 | |  | |  |
| Pars Orbitalis | -0.0399 | 0.0229 | 0.94 | -0.0008 | 0.0042 | | 0.93 | | -0.0020 | 0.0042 | | 0.88 | |  | |  |
| Pars Triangularis | 0.0234 | 0.0246 | 0.94 | -0.0012 | 0.0045 | | 0.93 | | 0.0001 | 0.0045 | | 0.98 | |  | |  |
| Pericalcarine | -0.0310 | 0.0267 | 0.94 | -0.0262 | 0.0055 | | **5.19E-05** | | -0.02642 | 0.0053 | | **2.58E-05** | |  | |  |
| Postcentral | -0.0004 | 0.0229 | 0.99 | 0.0007 | 0.0039 | | 0.93 | | 0.0009 | 0.0038 | | 0.89 | |  | |  |
| Posterior Cingulate | -0.0211 | 0.0224 | 0.94 | -0.0039 | 0.0040 | | 0.92 | | -0.0042 | 0.0040 | | 0.87 | |  | |  |
| Precentral | -0.0272 | 0.0225 | 0.94 | -0.0067 | 0.0040 | | 0.47 | | -0.0072 | 0.0040 | | 0.46 | |  | |  |
| Precuneus | -0.0038 | 0.0224 | 0.94 | -0.0067 | 0.0042 | | 0.47 | | -0.0063 | 0.0041 | | 0.50 | |  | |  |
| Rostral Ant Cingulate | -0.0073 | 0.0223 | 0.94 | 0.0022 | 0.0040 | | 0.93 | | 0.0022 | 0.0040 | | 0.88 | |  | |  |
| Rostral Mid Frontal | -0.0213 | 0.0224 | 0.94 | -0.0014 | 0.0039 | | 0.93 | | -0.0018 | 0.0038 | | 0.88 | |  | |  |
| Sup Frontal | 0.0144 | 0.0214 | 0.94 | 0.0019 | 0.0037 | | 0.93 | | 0.0026 | 0.0037 | | 0.88 | |  | |  |
| Sup Parietal | -0.0035 | 0.0239 | 0.94 | -0.0045 | 0.0044 | | 0.92 | | -0.0041 | 0.0044 | | 0.88 | |  | |  |
| Sup Temporal | 0.0180 | 0.0219 | 0.94 | -0.0031 | 0.0039 | | 0.93 | | -0.0020 | 0.0038 | | 0.88 | |  | |  |
| Supramarginal | 0.0203 | 0.0224 | 0.94 | -0.0073 | 0.0041 | | 0.46 | | -0.0058 | 0.0040 | | 0.53 | |  | |  |
| Temporal Pole* | -0.0058 | 0.0229 | 0.94 |  |  | |  | |  |  | |  | |  | |  |
| Transverse Temporal | 0.0311 | 0.0254 | 0.94 | -0.0031 | 0.0046 | | 0.93 | | -0.0014 | 0.0046 | | 0.89 | |  | |  |
|  |  |  |  |  |  | |  | |  |  | |  | |  | |  |
|  |  |  |  |  |  | |  | |  |  | |  | |  | |  |
|  |  |  |  |  |  | |  | |  |  | |  | |  | |  |
| **Emotional Neglect** | **GS (n=1,024)** | | | **UKB (n=27,202)** | | | | | **Mega-Analysis (n=28,226)** | | | | | |  | |
| **Region** | **Beta** | **Std Err** | **P(FDR)** | **Beta** | | **Std Err** | | **P(FDR)** | **Beta** | | **Std Err** | | **P(FDR)** | |  | |
| Banks STS | -0.0130 | 0.0232 | 0.94 | -0.0146 | | 0.0043 | | **0.0086** | -0.0146 | | 0.0043 | | **0.0058** | |  | |
| Caudal Ant Cingulate** | 0.0020 | 0.0218 | 0.95 | -0.0072 | | 0.0042 | | 0.23 | -0.0069 | | 0.0041 | | 0.24 | |  | |
| Caudal Mid Frontal | 0.0115 | 0.0237 | 0.94 | -0.0010 | | 0.0043 | | 0.84 | -0.0004 | | 0.0042 | | 0.95 | |  | |
| Cuneus | -0.0566 | 0.0238 | 0.60 | -0.0085 | | 0.0049 | | 0.23 | -0.0102 | | 0.0048 | | 0.12 | |  | |
| Entorhinal | 0.0051 | 0.0235 | 0.94 | -0.0026 | | 0.0049 | | 0.70 | -0.0024 | | 0.0048 | | 0.73 | |  | |
| Frontal Pole | -0.0204 | 0.0230 | 0.94 | -0.0059 | | 0.0042 | | 0.31 | -0.0064 | | 0.0041 | | 0.27 | |  | |
| Fusiform | -0.0085 | 0.0223 | 0.94 | -0.0087 | | 0.0039 | | 0.11 | -0.0084 | | 0.0039 | | 0.12 | |  | |
| Inf Parietal | 0.0093 | 0.0226 | 0.94 | -0.0172 | | 0.0042 | | **0.0016** | -0.0162 | | 0.0042 | | **0.0019** | |  | |
| Inf Temporal | 0.0077 | 0.0223 | 0.94 | -0.0129 | | 0.0039 | | **0.0094** | -0.0121 | | 0.0039 | | **0.010** | |  | |
| Insula | 0.0072 | 0.0210 | 0.94 | -0.0020 | | 0.0040 | | 0.70 | -0.0016 | | 0.0039 | | 0.78 | |  | |
| Isthmus Cingulate | 0.0014 | 0.0222 | 0.95 | 0.0013 | | 0.0041 | | 0.82 | 0.0013 | | 0.0040 | | 0.79 | |  | |
| Lat Occipital | -0.0120 | 0.0222 | 0.94 | -0.0011 | | 0.0043 | | 0.84 | -0.0015 | | 0.0042 | | 0.79 | |  | |
| Lat Orbitofrontal | 0.0434 | 0.0228 | 0.82 | -0.0061 | | 0.0040 | | 0.27 | -0.0043 | | 0.0039 | | 0.50 | |  | |
| Lingual | 0.0306 | 0.0243 | 0.94 | -0.0053 | | 0.0049 | | 0.44 | -0.0039 | | 0.0048 | | 0.64 | |  | |
| Med Orbitofrontal | 0.0095 | 0.0214 | 0.94 | -0.0071 | | 0.0035 | | 0.14 | -0.0065 | | 0.0035 | | 0.20 | |  | |
| Middle Temporal | -0.0177 | 0.0218 | 0.94 | -0.0147 | | 0.0039 | | **0.0026** | -0.0148 | | 0.0039 | | **0.0019** | |  | |
| Paracentral | 0.0378 | 0.0228 | 0.82 | -0.0039 | | 0.0042 | | 0.49 | -0.0023 | | 0.0042 | | 0.73 | |  | |
| Parahippocampal | 0.0042 | 0.0235 | 0.94 | -0.0057 | | 0.0044 | | 0.35 | -0.0054 | | 0.0043 | | 0.40 | |  | |
| Pars Opercularis | 0.0066 | 0.0235 | 0.94 | 0.0026 | | 0.0045 | | 0.69 | 0.0028 | | 0.0044 | | 0.71 | |  | |
| Pars Orbitalis | -0.0152 | 0.0226 | 0.94 | 0.0027 | | 0.0042 | | 0.66 | 0.0021 | | 0.0041 | | 0.73 | |  | |
| Pars Triangularis | 0.0125 | 0.0242 | 0.94 | -0.0003 | | 0.0045 | | 0.95 | 0.0002 | | 0.0044 | | 0.96 | |  | |
| Pericalcarine | -0.0467 | 0.0262 | 0.82 | -0.0172 | | 0.0054 | | **0.010** | -0.0180 | | 0.0053 | | **0.0058** | |  | |
| Postcentral | -0.0101 | 0.0226 | 0.94 | -0.0120 | | 0.0039 | | **0.011** | -0.0120 | | 0.0038 | | 0.010 | |  | |
| Posterior Cingulate | 0.0340 | 0.0220 | 0.84 | -0.0045 | | 0.0040 | | 0.43 | -0.0031 | | 0.0039 | | 0.64 | |  | |
| Precentral | -0.0216 | 0.0221 | 0.94 | -0.0063 | | 0.0040 | | 0.27 | -0.0068 | | 0.0039 | | 0.24 | |  | |
| Precuneus | 0.0031 | 0.0220 | 0.94 | -0.0068 | | 0.0041 | | 0.25 | -0.0063 | | 0.0041 | | 0.27 | |  | |
| Rostral Ant Cingulate | 0.0072 | 0.0219 | 0.94 | -0.0039 | | 0.0040 | | 0.48 | -0.0035 | | 0.0039 | | 0.61 | |  | |
| Rostral Mid Frontal | -0.0035 | 0.0220 | 0.94 | -0.0097 | | 0.0039 | | 0.057 | -0.0094 | | 0.0038 | | 0.064 | |  | |
| Sup Frontal | 0.0131 | 0.0210 | 0.94 | -0.0044 | | 0.0037 | | 0.41 | -0.0037 | | 0.0036 | | 0.55 | |  | |
| Sup Parietal | -0.0130 | 0.0236 | 0.94 | 0.0028 | | 0.0044 | | 0.66 | 0.0024 | | 0.0043 | | 0.73 | |  | |
| Sup Temporal | 0.0047 | 0.0216 | 0.94 | -0.0058 | | 0.0039 | | 0.27 | -0.0054 | | 0.0038 | | 0.32 | |  | |
| Supramarginal | 0.0315 | 0.0220 | 0.87 | -0.0084 | | 0.0040 | | 0.14 | -0.0067 | | 0.0040 | | 0.24 | |  | |
| Temporal Pole* | 0.0167 | 0.0226 | 0.94 |  | |  | |  |  | |  | |  | |  | |
| Transverse Temporal | 0.0242 | 0.0250 | 0.94 | -0.0047 | | 0.0046 | | 0.46 | -0.0034 | | 0.0045 | | 0.65 | |  | |
|  |  |  |  |  | |  | |  |  | |  | |  | |  | |
|  |  |  |  |  | |  | |  |  | |  | |  | |  | |
| **Physical Neglect** | **GS (n=1,024)** | | | **UKB (n=27,202)** | | | | | **Mega-Analysis (n=28,226)** | | | | | |  | |
| **Region** | **Beta** | **Std Err** | **P(FDR)** | **Beta** | | **Std Err** | | **P(FDR)** | **Beta** | | **Std Err** | | **P(FDR)** | |  | |
| Banks STS | -0.0012 | 0.0232 | 0.96 | -0.0127 | | 0.0043 | | **0.022** | -0.0129 | | 0.0043 | | **0.012** | |  | |
| Caudal Ant Cingulate** | 0.0073 | 0.0218 | 0.85 | 0.0026 | | 0.0042 | | 0.71 | 0.0024 | | 0.0041 | | 0.71 | |  | |
| Caudal Mid Frontal | 0.0375 | 0.0237 | 0.55 | 0.0058 | | 0.0043 | | 0.28 | 0.0068 | | 0.0043 | | 0.19 | |  | |
| Cuneus | -0.0548 | 0.0238 | 0.40 | -0.0138 | | 0.0049 | | **0.024** | -0.0155 | | 0.0048 | | **0.0089** | |  | |
| Entorhinal | 0.0014 | 0.0236 | 0.96 | -0.0057 | | 0.0049 | | 0.33 | -0.0057 | | 0.0048 | | 0.33 | |  | |
| Frontal Pole | -0.0144 | 0.0231 | 0.85 | -0.0075 | | 0.0042 | | 0.15 | -0.0079 | | 0.0041 | | 0.11 | |  | |
| Fusiform | -0.0327 | 0.0223 | 0.55 | -0.0074 | | 0.0039 | | 0.12 | -0.0086 | | 0.0039 | | 0.059 | |  | |
| Inf Parietal | -0.0229 | 0.0226 | 0.67 | -0.0094 | | 0.0043 | | 0.058 | -0.0103 | | 0.0042 | | **0.034** | |  | |
| Inf Temporal | 0.0140 | 0.0223 | 0.85 | -0.0103 | | 0.0040 | | **0.030** | -0.0100 | | 0.0039 | | **0.029** | |  | |
| Insula | 0.0111 | 0.0210 | 0.85 | -0.0091 | | 0.0040 | | 0.058 | -0.0083 | | 0.0039 | | 0.067 | |  | |
| Isthmus Cingulate | 0.0363 | 0.0222 | 0.55 | 0.0017 | | 0.0041 | | 0.78 | 0.0026 | | 0.0040 | | 0.68 | |  | |
| Lat Occipital | -0.0157 | 0.0222 | 0.85 | -0.0133 | | 0.0043 | | **0.019** | -0.0137 | | 0.0043 | | **0.0089** | |  | |
| Lat Orbitofrontal | 0.0233 | 0.0229 | 0.67 | -0.0142 | | 0.0040 | | **0.013** | -0.0131 | | 0.0039 | | **0.0089** | |  | |
| Lingual | 0.0272 | 0.0243 | 0.67 | -0.0138 | | 0.0049 | | **0.024** | -0.0127 | | 0.0048 | | **0.028** | |  | |
| Med Orbitofrontal | -0.0105 | 0.0214 | 0.85 | -0.0079 | | 0.0035 | | 0.058 | -0.0083 | | 0.0035 | | **0.040** | |  | |
| Middle Temporal | -0.0097 | 0.0218 | 0.85 | -0.0125 | | 0.0039 | | **0.019** | -0.0129 | | 0.0039 | | **0.0089** | |  | |
| Paracentral | 0.0184 | 0.0228 | 0.84 | 0.0015 | | 0.0042 | | 0.80 | 0.0018 | | 0.0042 | | 0.76 | |  | |
| Parahippocampal | -0.0447 | 0.0235 | 0.55 | -0.0062 | | 0.0044 | | 0.26 | -0.0080 | | 0.0043 | | 0.11 | |  | |
| Pars Opercularis | 0.0113 | 0.0236 | 0.85 | 0.0019 | | 0.0045 | | 0.78 | 0.0021 | | 0.0044 | | 0.76 | |  | |
| Pars Orbitalis | -0.0115 | 0.0226 | 0.85 | -0.0107 | | 0.0042 | | **0.034** | -0.0108 | | 0.0041 | | **0.028** | |  | |
| Pars Triangularis | 0.0089 | 0.0242 | 0.85 | -0.0066 | | 0.0045 | | 0.25 | -0.0061 | | 0.0044 | | 0.26 | |  | |
| Pericalcarine | -0.0319 | 0.0263 | 0.67 | -0.0145 | | 0.0054 | | **0.028** | -0.0153 | | 0.0053 | | **0.017** | |  | |
| Postcentral | -0.0028 | 0.0226 | 0.96 | -0.0048 | | 0.0039 | | 0.33 | -0.0050 | | 0.0038 | | 0.28 | |  | |
| Posterior Cingulate | 0.0499 | 0.0220 | 0.40 | -0.0005 | | 0.0040 | | 0.92 | 0.0009 | | 0.0040 | | 0.84 | |  | |
| Precentral | -0.0114 | 0.0222 | 0.85 | -0.0006 | | 0.0040 | | 0.92 | -0.0011 | | 0.0039 | | 0.84 | |  | |
| Precuneus | -0.0070 | 0.0220 | 0.85 | -0.0130 | | 0.0041 | | **0.019** | -0.0130 | | 0.0041 | | **0.0089** | |  | |
| Rostral Ant Cingulate | 0.0257 | 0.0219 | 0.67 | 0.0004 | | 0.0040 | | 0.92 | 0.0008 | | 0.0039 | | 0.84 | |  | |
| Rostral Mid Frontal | -0.0321 | 0.0220 | 0.55 | -0.0106 | | 0.0039 | | **0.025** | -0.0118 | | 0.0038 | | **0.012** | |  | |
| Sup Frontal | 0.0077 | 0.0211 | 0.85 | -0.0017 | | 0.0037 | | 0.78 | -0.0016 | | 0.0037 | | 0.76 | |  | |
| Sup Parietal | -0.0409 | 0.0236 | 0.55 | -0.0099 | | 0.0044 | | 0.058 | -0.0114 | | 0.0043 | | **0.028** | |  | |
| Sup Temporal | 0.0237 | 0.0216 | 0.67 | -0.0046 | | 0.0039 | | 0.33 | -0.0039 | | 0.0038 | | 0.43 | |  | |
| Supramarginal | 0.0222 | 0.0221 | 0.67 | -0.0071 | | 0.0040 | | 0.15 | -0.0062 | | 0.0040 | | 0.20 | |  | |
| Temporal Pole* | -0.0033 | 0.0226 | 0.96 |  | |  | |  |  | |  | |  | |  | |
| Transverse Temporal | 0.0399 | 0.0250 | 0.55 | -0.0026 | | 0.0046 | | 0.72 | -0.0010 | | 0.0045 | | 0.84 | |  | |
|  |  |  |  |  | |  | |  |  | |  | |  | |  | |

| **Abuse Composite Score** | **GS (n=1,024)** | | | | | **UKB (n=27,202)** | | | | | **Mega-Analysis (n=28,226)** | | | | |  | |
| --- | --- | --- | --- | --- | --- | --- | --- | --- | --- | --- | --- | --- | --- | --- | --- | --- | --- |
| **Region** | **Beta** | | **Std Err** | | **P(FDR)** | **Beta** | | **Std Err** | | **P(FDR)** | **Beta** | **Std Err** | | **P(FDR)** | |  | |
| Banks STS | 0.0132 | | 0.0235 | | 0.82 | -0.0051 | | 0.0044 | | 0.41 | -0.0045 | 0.0043 | | 0.44 | |  | |
| Caudal Ant Cingulate** | -0.0234 | | 0.0221 | | 0.76 | -0.0032 | | 0.0042 | | 0.52 | -0.0040 | 0.0041 | | 0.44 | |  | |
| Caudal Mid Frontal | -0.0033 | | 0.0241 | | 0.95 | 0.0043 | | 0.0043 | | 0.45 | 0.0040 | 0.0043 | | 0.44 | |  | |
| Cuneus | -0.0553 | | 0.0242 | | 0.49 | -0.0159 | | 0.0049 | | **0.013** | -0.0174 | 0.0048 | | **0.0045** | |  | |
| Entorhinal | -0.0089 | | 0.0239 | | 0.89 | -0.0048 | | 0.0049 | | 0.45 | -0.0055 | 0.0048 | | 0.40 | |  | |
| Frontal Pole | -0.0244 | | 0.0233 | | 0.76 | -0.0076 | | 0.0042 | | 0.16 | -0.0076 | 0.0041 | | 0.15 | |  | |
| Fusiform | -0.0204 | | 0.0226 | | 0.79 | -0.0109 | | 0.0039 | | **0.037** | -0.0110 | 0.0039 | | **0.026** | |  | |
| Inf Parietal | -0.0163 | | 0.0229 | | 0.82 | -0.0144 | | 0.0043 | | **0.012** | -0.0147 | 0.0042 | | **0.0053** | |  | |
| Inf Temporal | -0.0042 | | 0.0226 | | 0.95 | -0.0078 | | 0.0040 | | 0.13 | -0.0077 | 0.0039 | | 0.12 | |  | |
| Insula | -0.0129 | | 0.0213 | | 0.82 | -0.0049 | | 0.0040 | | 0.38 | -0.0052 | 0.0039 | | 0.32 | |  | |
| Isthmus Cingulate | -0.0271 | | 0.0225 | | 0.76 | -0.0033 | | 0.0041 | | 0.51 | -0.0042 | 0.0041 | | 0.44 | |  | |
| Lat Occipital | -0.0380 | | 0.0225 | | 0.49 | -0.0127 | | 0.0044 | | **0.029** | -0.0137 | 0.0043 | | **0.011** | |  | |
| Lat Orbitofrontal | -0.0151 | | 0.0232 | | 0.82 | -0.0013 | | 0.0040 | | 0.79 | -0.0017 | 0.0040 | | 0.73 | |  | |
| Lingual | -0.0275 | | 0.0247 | | 0.76 | -0.0108 | | 0.0050 | | 0.11 | -0.0118 | 0.0049 | | 0.064 | |  | |
| Med Orbitofrontal | -0.0184 | | 0.0217 | | 0.79 | -0.00002 | | 0.0036 | | 1.00 | -0.0005 | 0.0035 | | 0.92 | |  | |
| Middle Temporal | -0.0386 | | 0.0221 | | 0.49 | -0.0105 | | 0.0039 | | **0.042** | -0.0115 | 0.0039 | | **0.020** | |  | |
| Paracentral | 0.0159 | | 0.0231 | | 0.82 | 0.0066 | | 0.0043 | | 0.24 | 0.0070 | 0.0042 | | 0.20 | |  | |
| Parahippocampal | -0.0321 | | 0.0238 | | 0.67 | -0.0104 | | 0.0044 | | 0.076 | -0.0114 | 0.0043 | | **0.041** | |  | |
| Pars Opercularis | 0.0123 | | 0.0238 | | 0.82 | -0.0009 | | 0.0045 | | 0.86 | -0.0003 | 0.0044 | | 0.95 | |  | |
| Pars Orbitalis | -0.0371 | | 0.0229 | | 0.49 | -0.0052 | | 0.0042 | | 0.38 | -0.0062 | 0.0042 | | 0.26 | |  | |
| Pars Triangularis | -0.0128 | | 0.0245 | | 0.82 | -0.0026 | | 0.0045 | | 0.65 | -0.0026 | 0.0045 | | 0.63 | |  | |
| Pericalcarine | -0.0570 | | 0.0266 | | 0.49 | -0.0224 | | 0.0055 | | **0.0014** | -0.0241 | 0.0053 | | **2.29E-04** | |  | |
| Postcentral | -0.0150 | | 0.0229 | | 0.82 | -0.0074 | | 0.0039 | | 0.13 | -0.0076 | 0.0038 | | 0.12 | |  | |
| Posterior Cingulate | -0.0192 | | 0.0224 | | 0.79 | 0.0015 | | 0.0040 | | 0.78 | 0.0008 | 0.0040 | | 0.90 | |  | |
| Precentral | -0.0352 | | 0.0224 | | 0.49 | -0.0078 | | 0.0040 | | 0.13 | -0.0087 | 0.0040 | | 0.075 | |  | |
| Precuneus | -0.0226 | | 0.0223 | | 0.76 | -0.0087 | | 0.0042 | | 0.12 | -0.0092 | 0.0041 | | 0.072 | |  | |
| Rostral Ant Cingulate | -0.0084 | | 0.0222 | | 0.89 | 0.0062 | | 0.0040 | | 0.24 | 0.0057 | 0.0040 | | 0.28 | |  | |
| Rostral Mid Frontal | -0.0371 | | 0.0223 | | 0.49 | -0.0038 | | 0.0039 | | 0.45 | -0.0050 | 0.0038 | | 0.32 | |  | |
| Sup Frontal | 0.0003 | | 0.0213 | | 0.99 | 0.0036 | | 0.0037 | | 0.45 | 0.0035 | 0.0037 | | 0.44 | |  | |
| Sup Parietal | -0.0412 | | 0.0239 | | 0.49 | -0.0090 | | 0.0044 | | 0.13 | -0.0102 | 0.0044 | | 0.065 | |  | |
| Sup Temporal | 0.0032 | | 0.0219 | | 0.95 | -0.0035 | | 0.0039 | | 0.46 | -0.0032 | 0.0038 | | 0.47 | |  | |
| Supramarginal | 0.0011 | | 0.0224 | | 0.99 | -0.0101 | | 0.0041 | | 0.060 | -0.0095 | 0.0040 | | 0.064 | |  | |
| Temporal Pole* | 0.0064 | | 0.0229 | | 0.95 |  | |  | |  |  |  | |  | |  | |
| Transverse Temporal | -0.0058 | | 0.0253 | | 0.95 | -0.0044 | | 0.0046 | | 0.46 | -0.0040 | 0.0046 | | 0.47 | |  | |
|  |  | |  | |  |  | |  | |  |  |  | |  | |  | |
|  |  | |  | |  |  | |  | |  |  |  | |  | |  | |
|  |  |  | |  | |  |  | |  | |  | |  | |  | |  |
| **Neglect Composite Score** | **GS (n=1,024)** | | | | | **UKB (n=27,202)** | | | | | **Mega-Analysis (n=28,226)** | | | | | |  |
| **Region** | **Beta** | **Std Err** | | **P(FDR)** | | **Beta** | **Std Err** | | **P(FDR)** | | **Beta** | | **Std Err** | | **P(FDR)** | |  |
| Banks STS | -0.0099 | 0.0232 | | 0.83 | | -0.0170 | 0.0043 | | **9.50E-04** | | -0.01712 | | 0.0043 | | **6.60E-04** | |  |
| Caudal Ant Cingulate** | 0.0043 | 0.0218 | | 0.94 | | -0.0038 | 0.0042 | | 0.50 | | -0.0037 | | 0.0041 | | 0.49 | |  |
| Caudal Mid Frontal | 0.0231 | 0.0237 | | 0.83 | | 0.0023 | 0.0043 | | 0.63 | | 0.0031 | | 0.0042 | | 0.56 | |  |
| Cuneus | -0.0625 | 0.0238 | | 0.30 | | -0.0132 | 0.0049 | | **0.021** | | -0.0151 | | 0.0048 | | **0.0073** | |  |
| Entorhinal | 0.0042 | 0.0235 | | 0.94 | | -0.0048 | 0.0049 | | 0.46 | | -0.0046 | | 0.0048 | | 0.49 | |  |
| Frontal Pole | -0.0204 | 0.0230 | | 0.83 | | -0.0081 | 0.0042 | | 0.12 | | -0.0086 | | 0.0041 | | 0.093 | |  |
| Fusiform | -0.0190 | 0.0223 | | 0.83 | | -0.0100 | 0.0039 | | **0.028** | | -0.0103 | | 0.0039 | | **0.025** | |  |
| Inf Parietal | -0.0023 | 0.0226 | | 0.95 | | -0.0172 | 0.0042 | | **8.23E-04** | | -0.0168 | | 0.0042 | | **6.60E-04** | |  |
| Inf Temporal | 0.0110 | 0.0223 | | 0.83 | | -0.0145 | 0.0040 | | **0.0017** | | -0.0139 | | 0.0039 | | **0.0025** | |  |
| Insula | 0.0096 | 0.0210 | | 0.83 | | -0.0061 | 0.0040 | | 0.21 | | -0.0055 | | 0.0039 | | 0.27 | |  |
| Isthmus Cingulate | 0.0152 | 0.0222 | | 0.83 | | 0.0018 | 0.0041 | | 0.66 | | 0.0021 | | 0.0040 | | 0.63 | |  |
| Lat Occipital | -0.0149 | 0.0222 | | 0.83 | | -0.0077 | 0.0043 | | 0.16 | | -0.0081 | | 0.0043 | | 0.12 | |  |
| Lat Orbitofrontal | 0.0405 | 0.0228 | | 0.66 | | -0.0118 | 0.0040 | | **0.015** | | -0.0100 | | 0.0039 | | **0.031** | |  |
| Lingual | 0.0328 | 0.0243 | | 0.79 | | -0.0110 | 0.0049 | | 0.061 | | -0.0095 | | 0.0048 | | 0.11 | |  |
| Med Orbitofrontal | 0.0028 | 0.0214 | | 0.95 | | -0.0092 | 0.0035 | | **0.027** | | -0.0090 | | 0.0035 | | **0.031** | |  |
| Middle Temporal | -0.0166 | 0.0218 | | 0.83 | | -0.0170 | 0.0039 | | **4.42E-04** | | -0.01724 | | 0.0039 | | **2.58E-04** | |  |
| Paracentral | 0.0346 | 0.0228 | | 0.79 | | -0.0020 | 0.0042 | | 0.66 | | -0.0008 | | 0.0042 | | 0.85 | |  |
| Parahippocampal | -0.0145 | 0.0235 | | 0.83 | | -0.0073 | 0.0044 | | 0.17 | | -0.0078 | | 0.0043 | | 0.14 | |  |
| Pars Opercularis | 0.0092 | 0.0235 | | 0.83 | | 0.0028 | 0.0045 | | 0.58 | | 0.0031 | | 0.0044 | | 0.56 | |  |
| Pars Orbitalis | -0.0155 | 0.0226 | | 0.83 | | -0.0036 | 0.0042 | | 0.50 | | -0.0040 | | 0.0041 | | 0.49 | |  |
| Pars Triangularis | 0.0125 | 0.0242 | | 0.83 | | -0.0037 | 0.0045 | | 0.51 | | -0.0031 | | 0.0044 | | 0.56 | |  |
| Pericalcarine | -0.0463 | 0.0262 | | 0.66 | | -0.0198 | 0.0054 | | **0.0017** | | -0.0206 | | 0.0053 | | **8.66E-04** | |  |
| Postcentral | -0.0084 | 0.0226 | | 0.83 | | -0.0110 | 0.0039 | | **0.018** | | -0.0111 | | 0.0038 | | **0.015** | |  |
| Posterior Cingulate | 0.0442 | 0.0220 | | 0.66 | | -0.0035 | 0.0040 | | 0.50 | | -0.0019 | | 0.0039 | | 0.65 | |  |
| Precentral | -0.0201 | 0.0221 | | 0.83 | | -0.0048 | 0.0040 | | 0.38 | | -0.0054 | | 0.0039 | | 0.28 | |  |
| Precuneus | -0.0005 | 0.0220 | | 0.98 | | -0.0116 | 0.0041 | | **0.018** | | -0.0113 | | 0.0041 | | **0.020** | |  |
| Rostral Ant Cingulate | 0.0153 | 0.0219 | | 0.83 | | -0.0026 | 0.0040 | | 0.58 | | -0.0022 | | 0.0039 | | 0.63 | |  |
| Rostral Mid Frontal | -0.0151 | 0.0220 | | 0.83 | | -0.0125 | 0.0039 | | **0.0070** | | -0.0127 | | 0.0038 | | **0.005** | |  |
| Sup Frontal | 0.0125 | 0.0210 | | 0.83 | | -0.0040 | 0.0037 | | 0.44 | | -0.0035 | | 0.0036 | | 0.49 | |  |
| Sup Parietal | -0.0254 | 0.0236 | | 0.83 | | -0.0032 | 0.0044 | | 0.56 | | -0.0040 | | 0.0043 | | 0.49 | |  |
| Sup Temporal | 0.0127 | 0.0216 | | 0.83 | | -0.0065 | 0.0039 | | 0.17 | | -0.0059 | | 0.0038 | | 0.22 | |  |
| Supramarginal | 0.0315 | 0.0220 | | 0.79 | | -0.0097 | 0.0040 | | **0.042** | | -0.0081 | | 0.0040 | | 0.10 | |  |
| Temporal Pole* | 0.0108 | 0.0226 | | 0.83 | |  |  | |  | |  | |  | |  | |  |
| Transverse Temporal | 0.0331 | 0.0250 | | 0.79 | | -0.0047 | 0.0046 | | 0.46 | | -0.0032 | | 0.0045 | | 0.56 | |  |
|  |  |  | |  | |  |  | |  | |  | |  | |  | |  |

Cortical Thickness

| **Emotional Abuse** | **GS (n=1,024)** | | | | | | | | **UKB (n=27,202)** | | | | | | | | | | **Mega-Analysis (n=28,226)** | | | | | | | | | |  | |
| --- | --- | --- | --- | --- | --- | --- | --- | --- | --- | --- | --- | --- | --- | --- | --- | --- | --- | --- | --- | --- | --- | --- | --- | --- | --- | --- | --- | --- | --- | --- |
| **Region** | **Beta** | | **Std Err** | | | **P(FDR)** | | | **Beta** | | | | **Std Err** | | | **P(FDR)** | | | **Beta** | | | **Std Err** | | | | **P(FDR)** | | |  | |
| Banks STS | 0.0051 | | 0.0252 | | | 0.97 | | | -0.0103 | | | | 0.0050 | | | 0.093 | | | -0.0103 | | | 0.0049 | | | | 0.085 | | |  | |
| Caudal Ant Cingulate** | -0.0267 | | 0.0247 | | | 0.97 | | | -0.0015 | | | | 0.0048 | | | 0.83 | | | -0.0022 | | | 0.0047 | | | | 0.76 | | |  | |
| Caudal Mid Frontal | -0.0027 | | 0.0260 | | | 0.97 | | | -0.0094 | | | | 0.0053 | | | 0.15 | | | -0.0095 | | | 0.0052 | | | | 0.13 | | |  | |
| Cuneus | 0.0249 | | 0.0262 | | | 0.97 | | | 0.0003 | | | | 0.0054 | | | 0.99 | | | 0.0010 | | | 0.0053 | | | | 0.90 | | |  | |
| Entorhinal | -0.0216 | | 0.0269 | | | 0.97 | | | -0.0019 | | | | 0.0051 | | | 0.83 | | | -0.0031 | | | 0.0050 | | | | 0.69 | | |  | |
| Frontal Pole | 0.0345 | | 0.0253 | | | 0.97 | | | -0.0017 | | | | 0.0050 | | | 0.83 | | | -0.0004 | | | 0.0049 | | | | 0.96 | | |  | |
| Fusiform | 0.0074 | | 0.0273 | | | 0.97 | | | -0.0044 | | | | 0.0052 | | | 0.54 | | | -0.0045 | | | 0.0052 | | | | 0.51 | | |  | |
| Inf Parietal | -0.0204 | | 0.0242 | | | 0.97 | | | -0.0164 | | | | 0.0053 | | | **0.026** | | | -0.0169 | | | 0.0052 | | | | **0.014** | | |  | |
| Inf Temporal | -0.0095 | | 0.0254 | | | 0.97 | | | -0.0134 | | | | 0.0052 | | | **0.046** | | | -0.0135 | | | 0.0051 | | | | **0.033** | | |  | |
| Insula | -0.0048 | | 0.0260 | | | 0.97 | | | -0.0094 | | | | 0.0052 | | | 0.15 | | | -0.0098 | | | 0.0051 | | | | 0.11 | | |  | |
| Isthmus Cingulate | 0.0426 | | 0.0255 | | | 0.97 | | | -0.0002 | | | | 0.0051 | | | 0.99 | | | 0.0013 | | | 0.0050 | | | | 0.88 | | |  | |
| Lat Occipital | -0.0071 | | 0.0258 | | | 0.97 | | | 0.0025 | | | | 0.0054 | | | 0.79 | | | 0.0021 | | | 0.0053 | | | | 0.78 | | |  | |
| Lat Orbitofrontal | 0.0047 | | 0.0281 | | | 0.97 | | | -0.0087 | | | | 0.0053 | | | 0.18 | | | -0.0085 | | | 0.0052 | | | | 0.18 | | |  | |
| Lingual | 0.0627 | | 0.0267 | | | 0.39 | | | 0.0118 | | | | 0.0054 | | | 0.079 | | | 0.0133 | | | 0.0053 | | | | **0.040** | | |  | |
| Med Orbitofrontal | -0.0027 | | 0.0266 | | | 0.97 | | | -0.0135 | | | | 0.0051 | | | **0.046** | | | -0.0135 | | | 0.0050 | | | | **0.033** | | |  | |
| Middle Temporal | -0.0014 | | 0.0249 | | | 0.97 | | | -0.0084 | | | | 0.0051 | | | 0.18 | | | -0.0085 | | | 0.0050 | | | | 0.17 | | |  | |
| Paracentral | -0.0270 | | 0.0251 | | | 0.97 | | | -0.0162 | | | | 0.0054 | | | **0.026** | | | -0.0169 | | | 0.0052 | | | | **0.014** | | |  | |
| Parahippocampal | 0.0078 | | 0.0279 | | | 0.97 | | | -0.0060 | | | | 0.0052 | | | 0.36 | | | -0.0061 | | | 0.0051 | | | | 0.35 | | |  | |
| Pars Opercularis | 0.0035 | | 0.0244 | | | 0.97 | | | -0.0146 | | | | 0.0052 | | | **0.033** | | | -0.0143 | | | 0.0051 | | | | **0.032** | | |  | |
| Pars Orbitalis | 0.0019 | | 0.0252 | | | 0.97 | | | -0.0058 | | | | 0.0051 | | | 0.36 | | | -0.0057 | | | 0.0050 | | | | 0.36 | | |  | |
| Pars Triangularis | 0.0134 | | 0.0255 | | | 0.97 | | | -0.0190 | | | | 0.0052 | | | **0.0089** | | | -0.0182 | | | 0.0051 | | | | **0.012** | | |  | |
| Pericalcarine | 0.0622 | | 0.0273 | | | 0.39 | | | 0.0094 | | | | 0.0053 | | | 0.15 | | | 0.0110 | | | 0.0052 | | | | 0.085 | | |  | |
| Postcentral | 0.0187 | | 0.0241 | | | 0.97 | | | -0.0035 | | | | 0.0053 | | | 0.65 | | | -0.0029 | | | 0.0052 | | | | 0.69 | | |  | |
| Posterior Cingulate | 0.0216 | | 0.0260 | | | 0.97 | | | -0.0047 | | | | 0.0049 | | | 0.47 | | | -0.0042 | | | 0.0049 | | | | 0.51 | | |  | |
| Precentral | -0.0012 | | 0.0272 | | | 0.97 | | | -0.0157 | | | | 0.0053 | | | **0.026** | | | -0.0155 | | | 0.0052 | | | | **0.025** | | |  | |
| Precuneus | -0.0232 | | 0.0254 | | | 0.97 | | | -0.0133 | | | | 0.0054 | | | 0.057 | | | -0.0142 | | | 0.0053 | | | | **0.033** | | |  | |
| Rostral Ant Cingulate | -0.0286 | | 0.0258 | | | 0.97 | | | -0.0107 | | | | 0.0048 | | | 0.079 | | | -0.0114 | | | 0.0047 | | | | **0.047** | | |  | |
| Rostral Mid Frontal | -0.0107 | | 0.0259 | | | 0.97 | | | -0.0070 | | | | 0.0054 | | | 0.30 | | | -0.0073 | | | 0.0052 | | | | 0.26 | | |  | |
| Sup Frontal | -0.0315 | | 0.0269 | | | 0.97 | | | -0.0124 | | | | 0.0053 | | | 0.069 | | | -0.0135 | | | 0.0052 | | | | **0.034** | | |  | |
| Sup Parietal | 0.0009 | | 0.0246 | | | 0.97 | | | -0.0073 | | | | 0.0054 | | | 0.29 | | | -0.0074 | | | 0.0053 | | | | 0.26 | | |  | |
| Sup Temporal | 0.0333 | | 0.0262 | | | 0.97 | | | -0.0107 | | | | 0.0051 | | | 0.093 | | | -0.0095 | | | 0.0050 | | | | 0.12 | | |  | |
| Supramarginal | -0.0148 | | 0.0246 | | | 0.97 | | | -0.0115 | | | | 0.0052 | | | 0.079 | | | -0.0121 | | | 0.0051 | | | | 0.051 | | |  | |
| Temporal Pole* | -0.0152 | | 0.0271 | | | 0.97 | | |  | | | |  | | |  | | |  | | |  | | | |  | | |  | |
| Transverse Temporal | 0.0201 | | 0.0269 | | | 0.97 | | | -0.00003 | | | | 0.0053 | | | 1.00 | | | 0.0002 | | | 0.0052 | | | | 0.97 | | |  | |
|  |  | |  | | |  | | |  | | | |  | | |  | | |  | | |  | | | |  | | |  | |
|  |  | |  | | |  | | |  | | | |  | | |  | | |  | | |  | | | |  | | |  | |
| **Physical Abuse** | **GS (n=1,024)** | | | | | | | | **UKB (n=27,202)** | | | | | | | | | | **Mega-Analysis (n=28,226)** | | | | | | | | | |  | |
| **Region** | **Beta** | | **Std Err** | | | **P(FDR)** | | | **Beta** | | | | **Std Err** | | | **P(FDR)** | | | **Beta** | | | **Std Err** | | | | **P(FDR)** | | |  | |
| Banks STS | 0.0165 | | 0.0249 | | | 0.84 | | | -0.0068 | | | | 0.0050 | | | 0.57 | | | -0.0065 | | | 0.0049 | | | | 0.48 | | |  | |
| Caudal Ant Cingulate** | -0.0225 | | 0.0244 | | | 0.84 | | | -0.0067 | | | | 0.0047 | | | 0.57 | | | -0.0067 | | | 0.0047 | | | | 0.48 | | |  | |
| Caudal Mid Frontal | -0.0131 | | 0.0257 | | | 0.84 | | | -0.0036 | | | | 0.0053 | | | 0.79 | | | -0.0041 | | | 0.0052 | | | | 0.72 | | |  | |
| Cuneus | 0.0147 | | 0.0259 | | | 0.84 | | | 0.0038 | | | | 0.0054 | | | 0.79 | | | 0.0035 | | | 0.0053 | | | | 0.72 | | |  | |
| Entorhinal | -0.0470 | | 0.0265 | | | 0.49 | | | -0.0026 | | | | 0.0051 | | | 0.88 | | | -0.0042 | | | 0.0050 | | | | 0.72 | | |  | |
| Frontal Pole | 0.0008 | | 0.0251 | | | 0.98 | | | 0.0062 | | | | 0.0050 | | | 0.59 | | | 0.0064 | | | 0.0049 | | | | 0.48 | | |  | |
| Fusiform | 0.0114 | | 0.0270 | | | 0.85 | | | 0.0001 | | | | 0.0052 | | | 0.99 | | | 0.00004 | | | 0.0051 | | | | 0.99 | | |  | |
| Inf Parietal | -0.0260 | | 0.0240 | | | 0.84 | | | -0.0137 | | | | 0.0053 | | | 0.20 | | | -0.0144 | | | 0.0051 | | | | 0.091 | | |  | |
| Inf Temporal | 0.0116 | | 0.0251 | | | 0.84 | | | -0.0042 | | | | 0.0052 | | | 0.77 | | | -0.0037 | | | 0.0051 | | | | 0.72 | | |  | |
| Insula | -0.0076 | | 0.0257 | | | 0.90 | | | -0.0064 | | | | 0.0051 | | | 0.59 | | | -0.0072 | | | 0.0050 | | | | 0.48 | | |  | |
| Isthmus Cingulate | 0.0371 | | 0.0252 | | | 0.68 | | | 0.0034 | | | | 0.0051 | | | 0.79 | | | 0.0043 | | | 0.0050 | | | | 0.72 | | |  | |
| Lat Occipital | -0.0023 | | 0.0255 | | | 0.98 | | | 0.0045 | | | | 0.0054 | | | 0.77 | | | 0.0038 | | | 0.0053 | | | | 0.72 | | |  | |
| Lat Orbitofrontal | 0.0138 | | 0.0278 | | | 0.84 | | | -0.0019 | | | | 0.0053 | | | 0.88 | | | -0.0015 | | | 0.0052 | | | | 0.93 | | |  | |
| Lingual | 0.0781 | | 0.0263 | | | 0.11 | | | 0.0115 | | | | 0.0054 | | | 0.21 | | | 0.0130 | | | 0.0053 | | | | 0.091 | | |  | |
| Med Orbitofrontal | -0.0370 | | 0.0263 | | | 0.68 | | | -0.0024 | | | | 0.0051 | | | 0.88 | | | -0.0035 | | | 0.0050 | | | | 0.72 | | |  | |
| Middle Temporal | 0.0171 | | 0.0246 | | | 0.84 | | | -0.0041 | | | | 0.0051 | | | 0.77 | | | -0.0037 | | | 0.0050 | | | | 0.72 | | |  | |
| Paracentral | -0.0303 | | 0.0248 | | | 0.75 | | | -0.0110 | | | | 0.0053 | | | 0.22 | | | -0.0118 | | | 0.0052 | | | | 0.13 | | |  | |
| Parahippocampal | -0.0142 | | 0.0276 | | | 0.84 | | | -0.0090 | | | | 0.0052 | | | 0.39 | | | -0.0095 | | | 0.0051 | | | | 0.29 | | |  | |
| Pars Opercularis | 0.0005 | | 0.0241 | | | 0.98 | | | -0.0051 | | | | 0.0052 | | | 0.77 | | | -0.0052 | | | 0.0051 | | | | 0.67 | | |  | |
| Pars Orbitalis | -0.0162 | | 0.0249 | | | 0.84 | | | 0.0022 | | | | 0.0051 | | | 0.88 | | | 0.0017 | | | 0.0050 | | | | 0.93 | | |  | |
| Pars Triangularis | 0.0173 | | 0.0252 | | | 0.84 | | | -0.0013 | | | | 0.0052 | | | 0.94 | | | -0.0008 | | | 0.0051 | | | | 0.93 | | |  | |
| Pericalcarine | 0.0654 | | 0.0270 | | | 0.26 | | | 0.0080 | | | | 0.0053 | | | 0.55 | | | 0.0093 | | | 0.0052 | | | | 0.31 | | |  | |
| Postcentral | 0.0041 | | 0.0239 | | | 0.95 | | | -0.0008 | | | | 0.0053 | | | 0.96 | | | -0.0008 | | | 0.0051 | | | | 0.93 | | |  | |
| Posterior Cingulate | 0.0199 | | 0.0257 | | | 0.84 | | | 0.0018 | | | | 0.0049 | | | 0.88 | | | 0.0022 | | | 0.0048 | | | | 0.90 | | |  | |
| Precentral | -0.0168 | | 0.0269 | | | 0.84 | | | -0.0131 | | | | 0.0053 | | | 0.20 | | | -0.0136 | | | 0.0052 | | | | 0.091 | | |  | |
| Precuneus | -0.0431 | | 0.0251 | | | 0.49 | | | -0.0047 | | | | 0.0054 | | | 0.77 | | | -0.0066 | | | 0.0053 | | | | 0.49 | | |  | |
| Rostral Ant Cingulate | -0.0489 | | 0.0255 | | | 0.47 | | | -0.0108 | | | | 0.0048 | | | 0.20 | | | -0.0118 | | | 0.0047 | | | | 0.091 | | |  | |
| Rostral Mid Frontal | -0.0150 | | 0.0256 | | | 0.84 | | | -0.0009 | | | | 0.0053 | | | 0.96 | | | -0.0010 | | | 0.0052 | | | | 0.93 | | |  | |
| Sup Frontal | -0.0518 | | 0.0266 | | | 0.47 | | | -0.0051 | | | | 0.0053 | | | 0.77 | | | -0.0068 | | | 0.0052 | | | | 0.48 | | |  | |
| Sup Parietal | -0.0082 | | 0.0244 | | | 0.89 | | | -0.0004 | | | | 0.0054 | | | 0.99 | | | -0.0009 | | | 0.0053 | | | | 0.93 | | |  | |
| Sup Temporal | 0.0330 | | 0.0259 | | | 0.75 | | | -0.0020 | | | | 0.0051 | | | 0.88 | | | -0.0013 | | | 0.0050 | | | | 0.93 | | |  | |
| Supramarginal | -0.0148 | | 0.0243 | | | 0.84 | | | -0.0124 | | | | 0.0052 | | | 0.20 | | | -0.0129 | | | 0.0051 | | | | 0.091 | | |  | |
| Temporal Pole* | -0.0048 | | 0.0270 | | | 0.95 | | |  | | | |  | | |  | | |  | | |  | | | |  | | |  | |
| Transverse Temporal | 0.0132 | | 0.0266 | | | 0.84 | | | 0.0002 | | | | 0.0053 | | | 0.99 | | | -0.0003 | | | 0.0052 | | | | 0.98 | | |  | |
|  |  | |  | | |  | | |  | | | |  | | |  | | |  | | |  | | | |  | | |  | |
|  |  | |  | | |  | | |  | | | |  | | |  | | |  | | |  | | | |  | | |  | |
| **Sexual Abuse** | **GS (n=1,024)** | | | | | | | | **UKB (n=27,202)** | | | | | | | | | | **Mega-Analysis (n=28,226)** | | | | | | | | | |  | |
| **Region** | **Beta** | | **Std Err** | | | **P(FDR)** | | | **Beta** | | | | **Std Err** | | | **P(FDR)** | | | **Beta** | | | **Std Err** | | | | **P(FDR)** | | |  | |
| Banks STS | 0.0475 | | 0.0252 | | | 0.68 | | | 0.0002 | | | | 0.0050 | | | 0.97 | | | 0.0017 | | | 0.0049 | | | | 0.96 | | |  | |
| Caudal Ant Cingulate** | 0.0093 | | 0.0248 | | | 0.96 | | | 0.0033 | | | | 0.0048 | | | 0.88 | | | 0.0035 | | | 0.0047 | | | | 0.88 | | |  | |
| Caudal Mid Frontal | 0.0070 | | 0.0260 | | | 0.96 | | | 0.0065 | | | | 0.0053 | | | 0.75 | | | 0.0064 | | | 0.0052 | | | | 0.73 | | |  | |
| Cuneus | 0.0513 | | 0.0263 | | | 0.68 | | | 0.0058 | | | | 0.0054 | | | 0.79 | | | 0.0074 | | | 0.0053 | | | | 0.67 | | |  | |
| Entorhinal | -0.0141 | | 0.0269 | | | 0.96 | | | 0.0054 | | | | 0.0051 | | | 0.79 | | | 0.0045 | | | 0.0050 | | | | 0.86 | | |  | |
| Frontal Pole | -0.0161 | | 0.0254 | | | 0.96 | | | 0.0112 | | | | 0.0050 | | | 0.30 | | | 0.0099 | | | 0.0049 | | | | 0.28 | | |  | |
| Fusiform | 0.0130 | | 0.0273 | | | 0.96 | | | 0.0013 | | | | 0.0052 | | | 0.97 | | | 0.0015 | | | 0.0052 | | | | 0.96 | | |  | |
| Inf Parietal | 0.0020 | | 0.0243 | | | 0.96 | | | -0.0041 | | | | 0.0053 | | | 0.86 | | | -0.0041 | | | 0.0052 | | | | 0.88 | | |  | |
| Inf Temporal | 0.0191 | | 0.0254 | | | 0.96 | | | -0.0030 | | | | 0.0052 | | | 0.91 | | | -0.0025 | | | 0.0051 | | | | 0.94 | | |  | |
| Insula | -0.0049 | | 0.0260 | | | 0.96 | | | -0.0020 | | | | 0.0052 | | | 0.97 | | | -0.0022 | | | 0.0051 | | | | 0.94 | | |  | |
| Isthmus Cingulate | 0.0083 | | 0.0256 | | | 0.96 | | | 0.0079 | | | | 0.0051 | | | 0.68 | | | 0.0082 | | | 0.0050 | | | | 0.49 | | |  | |
| Lat Occipital | 0.0148 | | 0.0258 | | | 0.96 | | | 0.0120 | | | | 0.0054 | | | 0.30 | | | 0.0123 | | | 0.0053 | | | | 0.28 | | |  | |
| Lat Orbitofrontal | -0.0001 | | 0.0282 | | | 1.00 | | | -0.0027 | | | | 0.0053 | | | 0.93 | | | -0.0027 | | | 0.0052 | | | | 0.94 | | |  | |
| Lingual | 0.0260 | | 0.0268 | | | 0.96 | | | 0.0145 | | | | 0.0054 | | | 0.23 | | | 0.0152 | | | 0.0053 | | | | 0.13 | | |  | |
| Med Orbitofrontal | -0.0115 | | 0.0267 | | | 0.96 | | | 0.0011 | | | | 0.0051 | | | 0.97 | | | 0.0002 | | | 0.0050 | | | | 0.97 | | |  | |
| Middle Temporal | 0.0228 | | 0.0249 | | | 0.96 | | | -0.0002 | | | | 0.0051 | | | 0.97 | | | 0.0003 | | | 0.0050 | | | | 0.97 | | |  | |
| Paracentral | -0.0049 | | 0.0251 | | | 0.96 | | | 0.0009 | | | | 0.0054 | | | 0.97 | | | 0.0003 | | | 0.0052 | | | | 0.97 | | |  | |
| Parahippocampal | 0.0342 | | 0.0279 | | | 0.96 | | | 0.0004 | | | | 0.0052 | | | 0.97 | | | 0.0013 | | | 0.0051 | | | | 0.96 | | |  | |
| Pars Opercularis | 0.0175 | | 0.0244 | | | 0.96 | | | 0.0029 | | | | 0.0052 | | | 0.91 | | | 0.0033 | | | 0.0051 | | | | 0.90 | | |  | |
| Pars Orbitalis | -0.0032 | | 0.0252 | | | 0.96 | | | 0.0034 | | | | 0.0051 | | | 0.88 | | | 0.0028 | | | 0.0050 | | | | 0.94 | | |  | |
| Pars Triangularis | 0.0050 | | 0.0256 | | | 0.96 | | | -0.0008 | | | | 0.0052 | | | 0.97 | | | -0.0008 | | | 0.0051 | | | | 0.96 | | |  | |
| Pericalcarine | 0.0664 | | 0.0274 | | | 0.52 | | | 0.0067 | | | | 0.0053 | | | 0.75 | | | 0.0089 | | | 0.0052 | | | | 0.48 | | |  | |
| Postcentral | 0.0128 | | 0.0242 | | | 0.96 | | | 0.0110 | | | | 0.0053 | | | 0.30 | | | 0.0109 | | | 0.0052 | | | | 0.28 | | |  | |
| Posterior Cingulate | 0.0162 | | 0.0261 | | | 0.96 | | | 0.0098 | | | | 0.0049 | | | 0.31 | | | 0.0099 | | | 0.0049 | | | | 0.28 | | |  | |
| Precentral | 0.0103 | | 0.0272 | | | 0.96 | | | 0.0064 | | | | 0.0053 | | | 0.75 | | | 0.0065 | | | 0.0052 | | | | 0.73 | | |  | |
| Precuneus | 0.0099 | | 0.0255 | | | 0.96 | | | 0.0042 | | | | 0.0054 | | | 0.86 | | | 0.0043 | | | 0.0053 | | | | 0.88 | | |  | |
| Rostral Ant Cingulate | -0.0181 | | 0.0259 | | | 0.96 | | | -0.0040 | | | | 0.0048 | | | 0.86 | | | -0.0048 | | | 0.0047 | | | | 0.85 | | |  | |
| Rostral Mid Frontal | -0.0056 | | 0.0259 | | | 0.96 | | | 0.0064 | | | | 0.0054 | | | 0.75 | | | 0.0056 | | | 0.0052 | | | | 0.85 | | |  | |
| Sup Frontal | -0.0266 | | 0.0270 | | | 0.96 | | | 0.0049 | | | | 0.0053 | | | 0.86 | | | 0.0034 | | | 0.0052 | | | | 0.90 | | |  | |
| Sup Parietal | 0.0179 | | 0.0247 | | | 0.96 | | | 0.0049 | | | | 0.0054 | | | 0.86 | | | 0.0051 | | | 0.0053 | | | | 0.86 | | |  | |
| Sup Temporal | 0.0311 | | 0.0263 | | | 0.96 | | | 0.0010 | | | | 0.0051 | | | 0.97 | | | 0.0020 | | | 0.0050 | | | | 0.96 | | |  | |
| Supramarginal | -0.0033 | | 0.0247 | | | 0.96 | | | 0.0014 | | | | 0.0052 | | | 0.97 | | | 0.0010 | | | 0.0051 | | | | 0.96 | | |  | |
| Temporal Pole* | 0.0320 | | 0.0272 | | | 0.96 | | |  | | | |  | | |  | | |  | | |  | | | |  | | |  | |
| Transverse Temporal | -0.0225 | | 0.0269 | | | 0.96 | | | 0.0018 | | | | 0.0053 | | | 0.97 | | | 0.0010 | | | 0.0052 | | | | 0.96 | | |  | |
|  |  | |  | | |  | | |  | | | |  | | |  | | |  | | |  | | | |  | | |  | |
|  |  | |  | | |  | | |  | | | |  | | |  | | |  | | |  | | | |  | | |  | |
| **Emotional Neglect** | | **GS (n=1,024)** | | | | | | | | **UKB (n=27,202)** | | | | | | | | | | **Mega-Analysis (n=28,226)** | | | | | | | | | |  |
| **Region** | | **Beta** | | | **Std Err** | | | **P(FDR)** | | **Beta** | | | | **Std Err** | | | | **P(FDR)** | | **Beta** | | | | **Std Err** | | | | **P(FDR)** | |  |
| Banks STS | | 0.0011 | | | 0.0249 | | | 0.99 | | -0.0098 | | | | 0.0050 | | | | 0.41 | | -0.0094 | | | | 0.0049 | | | | 0.30 | |  |
| Caudal Ant Cingulate** | | -0.0491 | | | 0.0244 | | | 0.50 | | 0.0015 | | | | 0.0047 | | | | 0.83 | | -0.0004 | | | | 0.0046 | | | | 0.94 | |  |
| Caudal Mid Frontal | | -0.0198 | | | 0.0256 | | | 0.93 | | -0.0056 | | | | 0.0053 | | | | 0.55 | | -0.0064 | | | | 0.0052 | | | | 0.49 | |  |
| Cuneus | | -0.0134 | | | 0.0258 | | | 0.98 | | 0.0056 | | | | 0.0054 | | | | 0.55 | | 0.0046 | | | | 0.0053 | | | | 0.66 | |  |
| Entorhinal | | -0.0072 | | | 0.0265 | | | 0.99 | | 0.0052 | | | | 0.0051 | | | | 0.55 | | 0.0049 | | | | 0.0050 | | | | 0.59 | |  |
| Frontal Pole | | 0.0108 | | | 0.0250 | | | 0.99 | | 0.0013 | | | | 0.0049 | | | | 0.84 | | 0.0015 | | | | 0.0048 | | | | 0.82 | |  |
| Fusiform | | -0.0092 | | | 0.0269 | | | 0.99 | | 0.0043 | | | | 0.0052 | | | | 0.71 | | 0.0037 | | | | 0.0051 | | | | 0.73 | |  |
| Inf Parietal | | -0.0185 | | | 0.0239 | | | 0.93 | | -0.0060 | | | | 0.0052 | | | | 0.55 | | -0.0067 | | | | 0.0051 | | | | 0.48 | |  |
| Inf Temporal | | -0.0061 | | | 0.0250 | | | 0.99 | | 0.0019 | | | | 0.0051 | | | | 0.83 | | 0.0014 | | | | 0.0050 | | | | 0.83 | |  |
| Insula | | -0.0159 | | | 0.0256 | | | 0.97 | | 0.0061 | | | | 0.0051 | | | | 0.55 | | 0.0051 | | | | 0.0050 | | | | 0.59 | |  |
| Isthmus Cingulate | | 0.0005 | | | 0.0252 | | | 0.99 | | 0.0018 | | | | 0.0051 | | | | 0.83 | | 0.0017 | | | | 0.0050 | | | | 0.82 | |  |
| Lat Occipital | | -0.0282 | | | 0.0254 | | | 0.88 | | 0.0159 | | | | 0.0054 | | | | 0.11 | | 0.0139 | | | | 0.0053 | | | | 0.15 | |  |
| Lat Orbitofrontal | | -0.0170 | | | 0.0278 | | | 0.97 | | 0.0030 | | | | 0.0053 | | | | 0.80 | | 0.0022 | | | | 0.0052 | | | | 0.82 | |  |
| Lingual | | 0.0268 | | | 0.0264 | | | 0.88 | | 0.0138 | | | | 0.0053 | | | | 0.16 | | 0.0140 | | | | 0.0052 | | | | 0.15 | |  |
| Med Orbitofrontal | | -0.0283 | | | 0.0262 | | | 0.88 | | -0.0079 | | | | 0.0051 | | | | 0.44 | | -0.0087 | | | | 0.0050 | | | | 0.30 | |  |
| Middle Temporal | | -0.0138 | | | 0.0245 | | | 0.97 | | -0.0054 | | | | 0.0051 | | | | 0.55 | | -0.0061 | | | | 0.0050 | | | | 0.49 | |  |
| Paracentral | | -0.0697 | | | 0.0246 | | | 0.16 | | -0.0066 | | | | 0.0053 | | | | 0.55 | | -0.0091 | | | | 0.0052 | | | | 0.30 | |  |
| Parahippocampal | | -0.0043 | | | 0.0275 | | | 0.99 | | -0.0006 | | | | 0.0052 | | | | 0.94 | | -0.0008 | | | | 0.0051 | | | | 0.91 | |  |
| Pars Opercularis | | -0.0016 | | | 0.0241 | | | 0.99 | | -0.0095 | | | | 0.0052 | | | | 0.44 | | -0.0095 | | | | 0.0051 | | | | 0.30 | |  |
| Pars Orbitalis | | -0.0019 | | | 0.0248 | | | 0.99 | | -0.0031 | | | | 0.0050 | | | | 0.80 | | -0.0032 | | | | 0.0049 | | | | 0.73 | |  |
| Pars Triangularis | | -0.0219 | | | 0.0252 | | | 0.93 | | -0.0109 | | | | 0.0052 | | | | 0.39 | | -0.0115 | | | | 0.0051 | | | | 0.26 | |  |
| Pericalcarine | | 0.0259 | | | 0.0270 | | | 0.88 | | 0.0029 | | | | 0.0053 | | | | 0.80 | | 0.0035 | | | | 0.0052 | | | | 0.73 | |  |
| Postcentral | | -0.0152 | | | 0.0238 | | | 0.97 | | -0.0025 | | | | 0.0052 | | | | 0.83 | | -0.0034 | | | | 0.0051 | | | | 0.73 | |  |
| Posterior Cingulate | | 0.0069 | | | 0.0257 | | | 0.99 | | -0.0021 | | | | 0.0049 | | | | 0.83 | | -0.0019 | | | | 0.0048 | | | | 0.82 | |  |
| Precentral | | -0.0324 | | | 0.0268 | | | 0.88 | | -0.0082 | | | | 0.0053 | | | | 0.44 | | -0.0095 | | | | 0.0052 | | | | 0.30 | |  |
| Precuneus | | -0.0574 | | | 0.0250 | | | 0.37 | | -0.0041 | | | | 0.0054 | | | | 0.74 | | -0.0062 | | | | 0.0053 | | | | 0.49 | |  |
| Rostral Ant Cingulate | | -0.0301 | | | 0.0255 | | | 0.88 | | -0.0018 | | | | 0.0048 | | | | 0.83 | | -0.0029 | | | | 0.0047 | | | | 0.73 | |  |
| Rostral Mid Frontal | | -0.0270 | | | 0.0255 | | | 0.88 | | -0.0065 | | | | 0.0053 | | | | 0.55 | | -0.0075 | | | | 0.0052 | | | | 0.45 | |  |
| Sup Frontal | | -0.0394 | | | 0.0265 | | | 0.88 | | -0.0092 | | | | 0.0053 | | | | 0.44 | | -0.0105 | | | | 0.0052 | | | | 0.30 | |  |
| Sup Parietal | | -0.0373 | | | 0.0243 | | | 0.88 | | 0.0000 | | | | 0.0054 | | | | 0.99 | | -0.0017 | | | | 0.0053 | | | | 0.82 | |  |
| Sup Temporal | | 0.0038 | | | 0.0259 | | | 0.99 | | -0.0030 | | | | 0.0051 | | | | 0.80 | | -0.0029 | | | | 0.0050 | | | | 0.74 | |  |
| Supramarginal | | -0.0257 | | | 0.0243 | | | 0.88 | | -0.0060 | | | | 0.0052 | | | | 0.55 | | -0.0071 | | | | 0.0051 | | | | 0.46 | |  |
| Temporal Pole* | | 0.0099 | | | 0.0268 | | | 0.99 | |  | | | |  | | | |  | |  | | | |  | | | |  | |  |
| Transverse Temporal | | -0.0007 | | | 0.0265 | | | 0.99 | | 0.0085 | | | | 0.0053 | | | | 0.44 | | 0.0078 | | | | 0.0052 | | | | 0.43 | |  |
|  | |  | | |  | | |  | |  | | | |  | | | |  | |  | | | |  | | | |  | |  |
|  | |  | | |  | | |  | |  | | | |  | | | |  | |  | | | |  | | | |  | |  |
| **Physical Neglect** | | **GS (n=1,024)** | | | | | | | | **UKB (n=27,202)** | | | | | | | | | | **Mega-Analysis (n=28,226)** | | | | | | | | | |  |
| **Region** | | **Beta** | | | **Std Err** | | | **P(FDR)** | | **Beta** | | | | **Std Err** | | | | **P(FDR)** | | **Beta** | | | | **Std Err** | | | | **P(FDR)** | |  |
| Banks STS | | -0.0108 | | | 0.0249 | | | 0.91 | | -0.0117 | | | | 0.0050 | | | | 0.16 | | -0.0121 | | | | 0.0049 | | | | 0.11 | |  |
| Caudal Ant Cingulate** | | -0.0212 | | | 0.0244 | | | 0.82 | | -0.0003 | | | | 0.0047 | | | | 1.00 | | -0.0010 | | | | 0.0047 | | | | 0.95 | |  |
| Caudal Mid Frontal | | -0.0269 | | | 0.0256 | | | 0.82 | | -0.0042 | | | | 0.0053 | | | | 0.79 | | -0.0055 | | | | 0.0052 | | | | 0.60 | |  |
| Cuneus | | 0.0098 | | | 0.0259 | | | 0.92 | | 0.0052 | | | | 0.0054 | | | | 0.75 | | 0.0048 | | | | 0.0053 | | | | 0.69 | |  |
| Entorhinal | | -0.0331 | | | 0.0265 | | | 0.82 | | 0.0025 | | | | 0.0051 | | | | 0.82 | | 0.0010 | | | | 0.0050 | | | | 0.95 | |  |
| Frontal Pole | | 0.0149 | | | 0.0250 | | | 0.85 | | 0.0120 | | | | 0.0050 | | | | 0.16 | | 0.0122 | | | | 0.0049 | | | | 0.11 | |  |
| Fusiform | | 0.0064 | | | 0.0269 | | | 0.93 | | 0.00001 | | | | 0.0052 | | | | 1.00 | | -0.0002 | | | | 0.0051 | | | | 0.96 | |  |
| Inf Parietal | | -0.0333 | | | 0.0239 | | | 0.82 | | -0.0105 | | | | 0.0053 | | | | 0.25 | | -0.0118 | | | | 0.0051 | | | | 0.14 | |  |
| Inf Temporal | | -0.0013 | | | 0.0251 | | | 0.99 | | -0.0038 | | | | 0.0052 | | | | 0.79 | | -0.0039 | | | | 0.0051 | | | | 0.77 | |  |
| Insula | | -0.0021 | | | 0.0256 | | | 0.99 | | 0.0031 | | | | 0.0051 | | | | 0.79 | | 0.0022 | | | | 0.0050 | | | | 0.93 | |  |
| Isthmus Cingulate | | 0.0006 | | | 0.0252 | | | 0.99 | | -0.0018 | | | | 0.0051 | | | | 0.91 | | -0.0020 | | | | 0.0050 | | | | 0.93 | |  |
| Lat Occipital | | -0.0206 | | | 0.0254 | | | 0.82 | | 0.0038 | | | | 0.0054 | | | | 0.79 | | 0.0023 | | | | 0.0053 | | | | 0.93 | |  |
| Lat Orbitofrontal | | -0.0176 | | | 0.0278 | | | 0.85 | | 0.0030 | | | | 0.0053 | | | | 0.79 | | 0.0020 | | | | 0.0052 | | | | 0.93 | |  |
| Lingual | | 0.0328 | | | 0.0264 | | | 0.82 | | 0.0134 | | | | 0.0054 | | | | 0.16 | | 0.0132 | | | | 0.0053 | | | | 0.11 | |  |
| Med Orbitofrontal | | -0.0220 | | | 0.0263 | | | 0.82 | | -0.0001 | | | | 0.0051 | | | | 1.00 | | -0.0009 | | | | 0.0050 | | | | 0.95 | |  |
| Middle Temporal | | -0.0167 | | | 0.0245 | | | 0.84 | | -0.0103 | | | | 0.0051 | | | | 0.25 | | -0.0109 | | | | 0.0050 | | | | 0.16 | |  |
| Paracentral | | -0.0350 | | | 0.0247 | | | 0.82 | | -0.0052 | | | | 0.0053 | | | | 0.75 | | -0.0064 | | | | 0.0052 | | | | 0.53 | |  |
| Parahippocampal | | 0.0074 | | | 0.0275 | | | 0.93 | | -0.0002 | | | | 0.0052 | | | | 1.00 | | -0.0002 | | | | 0.0051 | | | | 0.96 | |  |
| Pars Opercularis | | 0.0054 | | | 0.0241 | | | 0.93 | | -0.0061 | | | | 0.0052 | | | | 0.71 | | -0.0063 | | | | 0.0051 | | | | 0.53 | |  |
| Pars Orbitalis | | -0.0065 | | | 0.0248 | | | 0.93 | | -0.0059 | | | | 0.0050 | | | | 0.71 | | -0.0060 | | | | 0.0049 | | | | 0.53 | |  |
| Pars Triangularis | | -0.0005 | | | 0.0252 | | | 0.99 | | -0.0072 | | | | 0.0052 | | | | 0.61 | | -0.0074 | | | | 0.0051 | | | | 0.53 | |  |
| Pericalcarine | | 0.0600 | | | 0.0271 | | | 0.72 | | 0.0060 | | | | 0.0053 | | | | 0.71 | | 0.0072 | | | | 0.0052 | | | | 0.53 | |  |
| Postcentral | | 0.0166 | | | 0.0238 | | | 0.84 | | -0.0007 | | | | 0.0052 | | | | 1.00 | | -0.0007 | | | | 0.0051 | | | | 0.95 | |  |
| Posterior Cingulate | | 0.0326 | | | 0.0257 | | | 0.82 | | -0.0014 | | | | 0.0049 | | | | 0.91 | | -0.0007 | | | | 0.0048 | | | | 0.95 | |  |
| Precentral | | -0.0275 | | | 0.0268 | | | 0.82 | | -0.0080 | | | | 0.0053 | | | | 0.54 | | -0.0093 | | | | 0.0052 | | | | 0.30 | |  |
| Precuneus | | -0.0509 | | | 0.0251 | | | 0.72 | | -0.0032 | | | | 0.0054 | | | | 0.79 | | -0.0056 | | | | 0.0053 | | | | 0.60 | |  |
| Rostral Ant Cingulate | | -0.0238 | | | 0.0255 | | | 0.82 | | -0.0035 | | | | 0.0048 | | | | 0.79 | | -0.0042 | | | | 0.0047 | | | | 0.69 | |  |
| Rostral Mid Frontal | | 0.0201 | | | 0.0256 | | | 0.82 | | -0.0030 | | | | 0.0053 | | | | 0.79 | | -0.0023 | | | | 0.0052 | | | | 0.93 | |  |
| Sup Frontal | | -0.0240 | | | 0.0266 | | | 0.82 | | -0.0053 | | | | 0.0053 | | | | 0.75 | | -0.0063 | | | | 0.0052 | | | | 0.53 | |  |
| Sup Parietal | | -0.0115 | | | 0.0243 | | | 0.90 | | -0.0016 | | | | 0.0054 | | | | 0.91 | | -0.0025 | | | | 0.0053 | | | | 0.93 | |  |
| Sup Temporal | | -0.0138 | | | 0.0259 | | | 0.88 | | -0.0120 | | | | 0.0051 | | | | 0.16 | | -0.0125 | | | | 0.0050 | | | | 0.11 | |  |
| Supramarginal | | -0.0286 | | | 0.0243 | | | 0.82 | | -0.0096 | | | | 0.0052 | | | | 0.32 | | -0.0109 | | | | 0.0051 | | | | 0.16 | |  |
| Temporal Pole* | | -0.0213 | | | 0.0268 | | | 0.82 | |  | | | |  | | | |  | |  | | | |  | | | |  | |  |
| Transverse Temporal | | -0.0244 | | | 0.0265 | | | 0.82 | | 0.0033 | | | | 0.0053 | | | | 0.79 | | 0.0013 | | | | 0.0052 | | | | 0.95 | |  |
|  | |  | | |  | | |  | |  | | | |  | | | |  | |  | | | |  | | | |  | |  |
| **Abuse Composite Score** | **GS (n=1,024)** | | | | | | | | **UKB (n=27,202)** | | | | | | | | | | **Mega-Analysis (n=28,226)** | | | | | | | | | |  | |
| **Region** | **Beta** | | **Std Err** | | | **P(FDR)** | | | **Beta** | | **Std Err** | | | | **P(FDR)** | | | | **Beta** | | **Std Err** | | | | **P(FDR)** | | | |  | |
| Banks STS | 0.0043 | | 0.0035 | | | 0.85 | | | -0.0086 | | 0.0050 | | | | 0.25 | | | | -0.0078 | | 0.0049 | | | | 0.25 | | | |  | |
| Caudal Ant Cingulate** | -0.0021 | | 0.0034 | | | 0.92 | | | -0.0028 | | 0.0048 | | | | 0.74 | | | | -0.0029 | | 0.0047 | | | | 0.71 | | | |  | |
| Caudal Mid Frontal | -0.0003 | | 0.0036 | | | 0.96 | | | -0.0042 | | 0.0053 | | | | 0.61 | | | | -0.0044 | | 0.0052 | | | | 0.54 | | | |  | |
| Cuneus | 0.0057 | | 0.0036 | | | 0.76 | | | 0.0041 | | 0.0054 | | | | 0.62 | | | | 0.0052 | | 0.0053 | | | | 0.48 | | | |  | |
| Entorhinal | -0.0045 | | 0.0037 | | | 0.85 | | | -0.0002 | | 0.0051 | | | | 0.97 | | | | -0.0018 | | 0.0050 | | | | 0.77 | | | |  | |
| Frontal Pole | 0.0012 | | 0.0035 | | | 0.92 | | | 0.0062 | | 0.0050 | | | | 0.36 | | | | 0.0063 | | 0.0049 | | | | 0.36 | | | |  | |
| Fusiform | 0.0019 | | 0.0037 | | | 0.92 | | | -0.0017 | | 0.0052 | | | | 0.82 | | | | -0.0018 | | 0.0052 | | | | 0.77 | | | |  | |
| Inf Parietal | -0.0024 | | 0.0033 | | | 0.92 | | | -0.0167 | | 0.0053 | | | | **0.026** | | | | -0.0170 | | 0.0052 | | | | **0.016** | | | |  | |
| Inf Temporal | 0.0012 | | 0.0035 | | | 0.92 | | | -0.0101 | | 0.0052 | | | | 0.19 | | | | -0.0097 | | 0.0051 | | | | 0.21 | | | |  | |
| Insula | -0.0010 | | 0.0036 | | | 0.92 | | | -0.0087 | | 0.0052 | | | | 0.25 | | | | -0.0094 | | 0.0051 | | | | 0.21 | | | |  | |
| Isthmus Cingulate | 0.0050 | | 0.0035 | | | 0.76 | | | 0.0044 | | 0.0051 | | | | 0.58 | | | | 0.0055 | | 0.0050 | | | | 0.42 | | | |  | |
| Lat Occipital | 0.0004 | | 0.0035 | | | 0.96 | | | 0.0079 | | 0.0054 | | | | 0.33 | | | | 0.0076 | | 0.0053 | | | | 0.31 | | | |  | |
| Lat Orbitofrontal | 0.0009 | | 0.0039 | | | 0.92 | | | -0.0065 | | 0.0053 | | | | 0.36 | | | | -0.0063 | | 0.0052 | | | | 0.38 | | | |  | |
| Lingual | 0.0093 | | 0.0037 | | | 0.19 | | | 0.0170 | | 0.0054 | | | | **0.026** | | | | 0.0184 | | 0.0053 | | | | **0.016** | | | |  | |
| Med Orbitofrontal | -0.0026 | | 0.0037 | | | 0.92 | | | -0.0078 | | 0.0051 | | | | 0.33 | | | | -0.0086 | | 0.0050 | | | | 0.23 | | | |  | |
| Middle Temporal | 0.0022 | | 0.0034 | | | 0.92 | | | -0.0064 | | 0.0051 | | | | 0.36 | | | | -0.0061 | | 0.0050 | | | | 0.38 | | | |  | |
| Paracentral | -0.0034 | | 0.0034 | | | 0.92 | | | -0.0134 | | 0.0054 | | | | 0.10 | | | | -0.0142 | | 0.0052 | | | | 0.056 | | | |  | |
| Parahippocampal | 0.0021 | | 0.0038 | | | 0.92 | | | -0.0073 | | 0.0052 | | | | 0.33 | | | | -0.0071 | | 0.0051 | | | | 0.32 | | | |  | |
| Pars Opercularis | 0.0014 | | 0.0033 | | | 0.92 | | | -0.0090 | | 0.0052 | | | | 0.25 | | | | -0.0087 | | 0.0051 | | | | 0.23 | | | |  | |
| Pars Orbitalis | -0.0008 | | 0.0035 | | | 0.92 | | | -0.0007 | | 0.0051 | | | | 0.92 | | | | -0.0011 | | 0.0050 | | | | 0.85 | | | |  | |
| Pars Triangularis | 0.0020 | | 0.0035 | | | 0.92 | | | -0.0108 | | 0.0052 | | | | 0.16 | | | | -0.0103 | | 0.0051 | | | | 0.18 | | | |  | |
| Pericalcarine | 0.0114 | | 0.0037 | | | 0.080 | | | 0.0112 | | 0.0053 | | | | 0.16 | | | | 0.0134 | | 0.0052 | | | | 0.070 | | | |  | |
| Postcentral | 0.0022 | | 0.0033 | | | 0.92 | | | 0.0018 | | 0.0053 | | | | 0.82 | | | | 0.0021 | | 0.0052 | | | | 0.77 | | | |  | |
| Posterior Cingulate | 0.0034 | | 0.0036 | | | 0.92 | | | 0.0020 | | 0.0049 | | | | 0.82 | | | | 0.0024 | | 0.0049 | | | | 0.77 | | | |  | |
| Precentral | -0.0002 | | 0.0037 | | | 0.97 | | | -0.0121 | | 0.0053 | | | | 0.15 | | | | -0.0121 | | 0.0052 | | | | 0.097 | | | |  | |
| Precuneus | -0.0028 | | 0.0035 | | | 0.92 | | | -0.0077 | | 0.0054 | | | | 0.33 | | | | -0.0087 | | 0.0053 | | | | 0.24 | | | |  | |
| Rostral Ant Cingulate | -0.0053 | | 0.0035 | | | 0.76 | | | -0.0122 | | 0.0048 | | | | 0.10 | | | | -0.0132 | | 0.0047 | | | | 0.056 | | | |  | |
| Rostral Mid Frontal | -0.0017 | | 0.0036 | | | 0.92 | | | -0.0018 | | 0.0054 | | | | 0.82 | | | | -0.0022 | | 0.0053 | | | | 0.77 | | | |  | |
| Sup Frontal | -0.0062 | | 0.0037 | | | 0.76 | | | -0.0072 | | 0.0053 | | | | 0.34 | | | | -0.0089 | | 0.0052 | | | | 0.23 | | | |  | |
| Sup Parietal | 0.0009 | | 0.0034 | | | 0.92 | | | -0.0022 | | 0.0054 | | | | 0.82 | | | | -0.0022 | | 0.0053 | | | | 0.77 | | | |  | |
| Sup Temporal | 0.0057 | | 0.0036 | | | 0.76 | | | -0.0062 | | 0.0051 | | | | 0.36 | | | | -0.0049 | | 0.0051 | | | | 0.48 | | | |  | |
| Supramarginal | -0.0018 | | 0.0034 | | | 0.92 | | | -0.0114 | | 0.0052 | | | | 0.16 | | | | -0.0120 | | 0.0051 | | | | 0.097 | | | |  | |
| Temporal Pole* | 0.0010 | | 0.0037 | | | 0.92 | | |  | |  | | | |  | | | |  | |  | | | |  | | | |  | |
| Transverse Temporal | 0.0004 | | 0.0037 | | | 0.96 | | | 0.0007 | | 0.0053 | | | | 0.92 | | | | 0.0002 | | 0.0052 | | | | 0.97 | | | |  | |
|  |  | |  | | |  | | |  | |  | | | |  | | | |  | |  | | | |  | | | |  | |
|  | |  | |  | | |  | | |  | |  | | | | |  | | |  | | |  | | | |  | | |  |
| **Neglect Composite Score** | | **GS (n=1,024)** | | | | | | | | **UKB (n=27,202)** | | | | | | | | | | **Mega-Analysis (n=28,226)** | | | | | | | | | |  |
| **Region** | | **Beta** | | **Std Err** | | | **P(FDR)** | | | **Beta** | | **Std Err** | | | | | **P(FDR)** | | | **Beta** | | | **Std Err** | | | | **P(FDR)** | | |  |
| Banks STS | | -0.0006 | | 0.0044 | | | 0.99 | | | -0.0131 | | 0.0050 | | | | | 0.14 | | | -0.0129 | | | 0.0049 | | | | 0.13 | | |  |
| Caudal Ant Cingulate** | | -0.0077 | | 0.0043 | | | 0.70 | | | 0.0009 | | 0.0047 | | | | | 0.94 | | | -0.0008 | | | 0.0046 | | | | 0.92 | | |  |
| Caudal Mid Frontal | | -0.0044 | | 0.0045 | | | 0.80 | | | -0.0062 | | 0.0053 | | | | | 0.45 | | | -0.0073 | | | 0.0052 | | | | 0.37 | | |  |
| Cuneus | | -0.0010 | | 0.0046 | | | 0.99 | | | 0.0067 | | 0.0054 | | | | | 0.45 | | | 0.0058 | | | 0.0053 | | | | 0.44 | | |  |
| Entorhinal | | -0.0032 | | 0.0047 | | | 0.94 | | | 0.0050 | | 0.0051 | | | | | 0.49 | | | 0.0042 | | | 0.0050 | | | | 0.55 | | |  |
| Frontal Pole | | 0.0024 | | 0.0044 | | | 0.99 | | | 0.0072 | | 0.0049 | | | | | 0.37 | | | 0.0073 | | | 0.0049 | | | | 0.33 | | |  |
| Fusiform | | -0.0007 | | 0.0047 | | | 0.99 | | | 0.0031 | | 0.0052 | | | | | 0.70 | | | 0.0025 | | | 0.0051 | | | | 0.75 | | |  |
| Inf Parietal | | -0.0047 | | 0.0042 | | | 0.70 | | | -0.0097 | | 0.0052 | | | | | 0.26 | | | -0.0108 | | | 0.0051 | | | | 0.16 | | |  |
| Inf Temporal | | -0.0009 | | 0.0044 | | | 0.99 | | | -0.0006 | | 0.0051 | | | | | 0.94 | | | -0.0010 | | | 0.0050 | | | | 0.92 | | |  |
| Insula | | -0.0022 | | 0.0045 | | | 0.99 | | | 0.0059 | | 0.0051 | | | | | 0.45 | | | 0.0049 | | | 0.0050 | | | | 0.50 | | |  |
| Isthmus Cingulate | | 0.0001 | | 0.0044 | | | 0.99 | | | 0.0004 | | 0.0051 | | | | | 0.94 | | | 0.0002 | | | 0.0050 | | | | 0.97 | | |  |
| Lat Occipital | | -0.0050 | | 0.0045 | | | 0.70 | | | 0.0133 | | 0.0054 | | | | | 0.16 | | | 0.0112 | | | 0.0053 | | | | 0.16 | | |  |
| Lat Orbitofrontal | | -0.0034 | | 0.0049 | | | 0.94 | | | 0.0037 | | 0.0053 | | | | | 0.66 | | | 0.0027 | | | 0.0052 | | | | 0.75 | | |  |
| Lingual | | 0.0057 | | 0.0047 | | | 0.70 | | | 0.0168 | | 0.0053 | | | | | 0.055 | | | 0.0168 | | | 0.0053 | | | | **0.045** | | |  |
| Med Orbitofrontal | | -0.0051 | | 0.0046 | | | 0.70 | | | -0.0057 | | 0.0051 | | | | | 0.46 | | | -0.0066 | | | 0.0050 | | | | 0.39 | | |  |
| Middle Temporal | | -0.0029 | | 0.0043 | | | 0.94 | | | -0.0093 | | 0.0051 | | | | | 0.26 | | | -0.0099 | | | 0.0050 | | | | 0.16 | | |  |
| Paracentral | | -0.0113 | | 0.0044 | | | 0.24 | | | -0.0074 | | 0.0053 | | | | | 0.39 | | | -0.0097 | | | 0.0052 | | | | 0.19 | | |  |
| Parahippocampal | | 0.0000 | | 0.0049 | | | 0.99 | | | -0.0005 | | 0.0052 | | | | | 0.94 | | | -0.0007 | | | 0.0051 | | | | 0.92 | | |  |
| Pars Opercularis | | 0.0002 | | 0.0042 | | | 0.99 | | | -0.0100 | | 0.0052 | | | | | 0.26 | | | -0.0100 | | | 0.0051 | | | | 0.16 | | |  |
| Pars Orbitalis | | -0.0007 | | 0.0044 | | | 0.99 | | | -0.0052 | | 0.0050 | | | | | 0.49 | | | -0.0054 | | | 0.0049 | | | | 0.44 | | |  |
| Pars Triangularis | | -0.0028 | | 0.0044 | | | 0.94 | | | -0.0115 | | 0.0052 | | | | | 0.22 | | | -0.0120 | | | 0.0051 | | | | 0.16 | | |  |
| Pericalcarine | | 0.0074 | | 0.0048 | | | 0.70 | | | 0.0052 | | 0.0053 | | | | | 0.49 | | | 0.0060 | | | 0.0052 | | | | 0.43 | | |  |
| Postcentral | | -0.0008 | | 0.0042 | | | 0.99 | | | -0.0022 | | 0.0052 | | | | | 0.80 | | | -0.0028 | | | 0.0051 | | | | 0.75 | | |  |
| Posterior Cingulate | | 0.0031 | | 0.0045 | | | 0.94 | | | -0.0022 | | 0.0049 | | | | | 0.80 | | | -0.0019 | | | 0.0048 | | | | 0.79 | | |  |
| Precentral | | -0.0060 | | 0.0047 | | | 0.70 | | | -0.0100 | | 0.0053 | | | | | 0.26 | | | -0.0114 | | | 0.0052 | | | | 0.16 | | |  |
| Precuneus | | -0.0109 | | 0.0044 | | | 0.24 | | | -0.0045 | | 0.0054 | | | | | 0.57 | | | -0.0071 | | | 0.0053 | | | | 0.39 | | |  |
| Rostral Ant Cingulate | | -0.0055 | | 0.0045 | | | 0.70 | | | -0.0031 | | 0.0048 | | | | | 0.69 | | | -0.0042 | | | 0.0047 | | | | 0.53 | | |  |
| Rostral Mid Frontal | | -0.0021 | | 0.0045 | | | 0.99 | | | -0.0062 | | 0.0053 | | | | | 0.45 | | | -0.0067 | | | 0.0052 | | | | 0.39 | | |  |
| Sup Frontal | | -0.0067 | | 0.0047 | | | 0.70 | | | -0.0093 | | 0.0053 | | | | | 0.26 | | | -0.0107 | | | 0.0052 | | | | 0.16 | | |  |
| Sup Parietal | | -0.0056 | | 0.0043 | | | 0.70 | | | -0.0008 | | 0.0054 | | | | | 0.94 | | | -0.0025 | | | 0.0053 | | | | 0.75 | | |  |
| Sup Temporal | | -0.0005 | | 0.0046 | | | 0.99 | | | -0.0084 | | 0.0051 | | | | | 0.31 | | | -0.0085 | | | 0.0050 | | | | 0.25 | | |  |
| Supramarginal | | -0.0053 | | 0.0043 | | | 0.70 | | | -0.0093 | | 0.0052 | | | | | 0.26 | | | -0.0105 | | | 0.0051 | | | | 0.16 | | |  |
| Temporal Pole* | | -0.0002 | | 0.0047 | | | 0.99 | | |  | |  | | | | |  | | |  | | |  | | | |  | | |  |
| Transverse Temporal | | -0.0018 | | 0.0047 | | | 0.99 | | | 0.0078 | | 0.0053 | | | | | 0.37 | | | 0.0065 | | | 0.0052 | | | | 0.39 | | |  |
|  | |  | |  | | |  | | |  | |  | | | | |  | | |  | | |  | | | |  | | |  |

Subcortical volumes

| **Emotional Abuse** | **GS (n=1,024)** | | | | | | **UKB (n=27,202)** | | | | | | **Mega-Analysis (n=28,226)** | | | | | |  | |
| --- | --- | --- | --- | --- | --- | --- | --- | --- | --- | --- | --- | --- | --- | --- | --- | --- | --- | --- | --- | --- |
| **Region** | **Beta** | | **Std Err** | | **P(FDR)** | | **Beta** | | **Std Err** | | **P(FDR)** | | **Beta** | | **Std Err** | | **P(FDR)** | |  | |
| Accumbens | -0.0489 | | 0.0233 | | 0.22 | | -0.0084 | | 0.0042 | | 0.12 | | -0.0092 | | 0.0042 | | 0.072 | |  | |
| Amygdala | -0.0213 | | 0.0247 | | 0.44 | | 0.0043 | | 0.0040 | | 0.44 | | 0.0037 | | 0.0040 | | 0.54 | |  | |
| Caudate | 0.0025 | | 0.0260 | | 0.92 | | 0.0036 | | 0.0050 | | 0.62 | | 0.0035 | | 0.0049 | | 0.54 | |  | |
| Hippocampus | -0.0431 | | 0.0250 | | 0.23 | | -0.0079 | | 0.0045 | | 0.16 | | -0.0092 | | 0.0044 | | 0.073 | |  | |
| Pallidum | -0.0326 | | 0.0242 | | 0.36 | | -0.0027 | | 0.0045 | | 0.63 | | -0.0035 | | 0.0044 | | 0.54 | |  | |
| Putamen | -0.0249 | | 0.0246 | | 0.44 | | 0.0013 | | 0.0048 | | 0.78 | | 0.0007 | | 0.0047 | | 0.88 | |  | |
| Thalamus | -0.0204 | | 0.0209 | | 0.44 | | -0.0137 | | 0.0040 | | **0.0034** | | -0.0133 | | 0.0040 | | **0.0030** | |  | |
| Ventral DC | -0.0424 | | 0.0220 | | 0.22 | | -0.0134 | | 0.0040 | | **0.0034** | | -0.0144 | | 0.0040 | | **0.0021** | |  | |
|  |  | |  | |  | |  | |  | |  | |  | |  | |  | |  | |
|  |  | |  | |  | |  | |  | |  | |  | |  | |  | |  | |
| **Physical Abuse** | **GS (n=1,024)** | | | | | | **UKB (n=27,202)** | | | | | | **Mega-Analysis (n=28,226)** | | | | | |  | |
| **Region** | **Beta** | | **Std Err** | | **P(FDR)** | | **Beta** | | **Std Err** | | **P(FDR)** | | **Beta** | | **Std Err** | | **P(FDR)** | |  | |
| Accumbens | -0.0730 | | 0.0230 | | **0.0050** | | -0.0043 | | 0.0042 | | 0.47 | | -0.0064 | | 0.0041 | | 0.20 | |  | |
| Amygdala | -0.0564 | | 0.0243 | | **0.028** | | 0.0037 | | 0.0040 | | 0.47 | | 0.0015 | | 0.0039 | | 0.80 | |  | |
| Caudate | -0.0251 | | 0.0257 | | 0.33 | | 0.0016 | | 0.0050 | | 0.75 | | 0.0003 | | 0.0049 | | 0.95 | |  | |
| Hippocampus | -0.0877 | | 0.0246 | | **0.0030** | | -0.0073 | | 0.0044 | | 0.20 | | -0.0106 | | 0.0044 | | **0.050** | |  | |
| Pallidum | -0.0588 | | 0.0239 | | **0.023** | | -0.0085 | | 0.0045 | | 0.18 | | -0.0103 | | 0.0044 | | **0.050** | |  | |
| Putamen | -0.0408 | | 0.0243 | | 0.11 | | 0.0037 | | 0.0048 | | 0.50 | | 0.0019 | | 0.0047 | | 0.80 | |  | |
| Thalamus | -0.0555 | | 0.0206 | | **0.014** | | -0.0073 | | 0.0040 | | 0.18 | | -0.0086 | | 0.0039 | | 0.057 | |  | |
| Ventral DC | -0.0676 | | 0.0217 | | **0.0050** | | -0.0212 | | 0.0040 | | **1.01E-06** | | -0.02302 | | 0.0039 | | **4.19E-08** | |  | |
|  |  | |  | |  | |  | |  | |  | |  | |  | |  | |  | |
|  |  | |  | |  | |  | |  | |  | |  | |  | |  | |  | |
| **Sexual Abuse** | **GS (n=1,024)** | | | | | | **UKB (n=27,202)** | | | | | | **Mega-Analysis (n=28,226)** | | | | | |  | |
| **Region** | **Beta** | | **Std Err** | | **P(FDR)** | | **Beta** | | **Std Err** | | **P(FDR)** | | **Beta** | | **Std Err** | | **P(FDR)** | |  | |
| Accumbens | 0.0124 | | 0.0234 | | 0.96 | | 0.0016 | | 0.0042 | | 0.80 | | 0.0029 | | 0.0042 | | 0.62 | |  | |
| Amygdala | 0.0032 | | 0.0247 | | 1.00 | | 0.0009 | | 0.0040 | | 0.82 | | 0.0016 | | 0.0040 | | 0.68 | |  | |
| Caudate | 0.0082 | | 0.0260 | | 1.00 | | 0.0027 | | 0.0050 | | 0.80 | | 0.0030 | | 0.0049 | | 0.62 | |  | |
| Hippocampus | -0.0209 | | 0.0251 | | 0.96 | | -0.0083 | | 0.0045 | | 0.12 | | -0.0083 | | 0.0044 | | 0.12 | |  | |
| Pallidum | 0.0132 | | 0.0243 | | 0.96 | | -0.0107 | | 0.0045 | | **0.045** | | -0.0093 | | 0.0044 | | 0.094 | |  | |
| Putamen | 0.0352 | | 0.0246 | | 0.62 | | 0.0024 | | 0.0048 | | 0.80 | | 0.0042 | | 0.0047 | | 0.60 | |  | |
| Thalamus | -0.0001 | | 0.0210 | | 1.00 | | -0.0172 | | 0.0040 | | **7.87E-05** | | -0.0160 | | 0.0040 | | **2.09E-04** | |  | |
| Ventral DC | -0.0314 | | 0.0221 | | 0.62 | | -0.0180 | | 0.0040 | | **6.15E-05** | | -0.0181 | | 0.0040 | | **3.87E-05** | |  | |
|  |  | |  | |  | |  | |  | |  | |  | |  | |  | |  | |
|  |  | |  | |  | |  | |  | |  | |  | |  | |  | |  | |
| **Emotional Neglect** | | **GS (n=1,024)** | | | | | | **UKB (n=27,202)** | | | | | | **Mega-Analysis (n=28,226)** | | | | | |  |
| **Region** | | **Beta** | | **Std Err** | | **P(FDR)** | | **Beta** | | **Std Err** | | **P(FDR)** | | **Beta** | | **Std Err** | | **P(FDR)** | |  |
| Accumbens | | -0.0743 | | 0.0229 | | **0.0099** | | 0.0008 | | 0.0042 | | 0.89 | | -0.0019 | | 0.0041 | | 0.86 | |  |
| Amygdala | | 0.0163 | | 0.0243 | | 0.81 | | 0.0042 | | 0.0040 | | 0.59 | | 0.0047 | | 0.0039 | | 0.46 | |  |
| Caudate | | -0.0137 | | 0.0257 | | 0.81 | | 0.0165 | | 0.0050 | | **0.0074** | | 0.0153 | | 0.0049 | | **0.013** | |  |
| Hippocampus | | -0.0318 | | 0.0247 | | 0.56 | | -0.0009 | | 0.0044 | | 0.89 | | -0.0020 | | 0.0044 | | 0.86 | |  |
| Pallidum | | -0.0124 | | 0.0239 | | 0.81 | | -0.0006 | | 0.0044 | | 0.89 | | -0.0011 | | 0.0044 | | 0.86 | |  |
| Putamen | | -0.0036 | | 0.0243 | | 0.93 | | 0.0128 | | 0.0047 | | **0.028** | | 0.0122 | | 0.0047 | | **0.037** | |  |
| Thalamus | | 0.0018 | | 0.0207 | | 0.93 | | -0.0009 | | 0.0040 | | 0.89 | | -0.0007 | | 0.0039 | | 0.86 | |  |
| Ventral DC | | -0.0272 | | 0.0217 | | 0.56 | | -0.0071 | | 0.0040 | | 0.20 | | -0.0078 | | 0.0039 | | 0.12 | |  |
|  | |  | |  | |  | |  | |  | |  | |  | |  | |  | |  |
|  | |  | |  | |  | |  | |  | |  | |  | |  | |  | |  |
| **Physical Neglect** | | **GS (n=1,024)** | | | | | | **UKB (n=27,202)** | | | | | | **Mega-Analysis (n=28,226)** | | | | | |  |
| **Region** | | **Beta** | | **Std Err** | | **P(FDR)** | | **Beta** | | **Std Err** | | **P(FDR)** | | **Beta** | | **Std Err** | | **P(FDR)** | |  |
| Accumbens | | -0.0681 | | 0.0230 | | **0.025** | | -0.0009 | | 0.0042 | | 0.82 | | -0.0037 | | 0.0041 | | 0.47 | |  |
| Amygdala | | -0.0133 | | 0.0244 | | 0.67 | | -0.0074 | | 0.0040 | | 0.10 | | -0.0080 | | 0.0039 | | 0.069 | |  |
| Caudate | | -0.0058 | | 0.0257 | | 0.82 | | 0.0046 | | 0.0050 | | 0.45 | | 0.0040 | | 0.0049 | | 0.47 | |  |
| Hippocampus | | -0.0509 | | 0.0247 | | 0.16 | | -0.0111 | | 0.0044 | | **0.033** | | -0.0131 | | 0.0044 | | **0.011** | |  |
| Pallidum | | -0.0145 | | 0.0239 | | 0.67 | | -0.0093 | | 0.0044 | | 0.071 | | -0.0102 | | 0.0044 | | **0.040** | |  |
| Putamen | | -0.0197 | | 0.0243 | | 0.67 | | 0.0040 | | 0.0048 | | 0.45 | | 0.0028 | | 0.0047 | | 0.55 | |  |
| Thalamus | | -0.0171 | | 0.0207 | | 0.67 | | -0.0106 | | 0.0040 | | **0.032** | | -0.0107 | | 0.0039 | | **0.018** | |  |
| Ventral DC | | -0.0368 | | 0.0217 | | 0.24 | | -0.0163 | | 0.0040 | | **3.58E-04** | | -0.01755 | | 0.0039 | | **6.72E-05** | |  |
|  | |  | |  | |  | |  | |  | |  | |  | |  | |  | |  |

| **Abuse Composite Score** | **GS (n=1,024)** | | | | | | **UKB (n=27,202)** | | | | | | **Mega-Analysis (n=28,226)** | | | | | |  | |
| --- | --- | --- | --- | --- | --- | --- | --- | --- | --- | --- | --- | --- | --- | --- | --- | --- | --- | --- | --- | --- |
| **Region** | **Beta** | | **Std Err** | | **P(FDR)** | | **Beta** | | **Std Err** | | **P(FDR)** | | **Beta** | | **Std Err** | | **P(FDR)** | |  | |
| Accumbens | -0.0411 | | 0.0233 | | 0.21 | | -0.0059 | | 0.0042 | | 0.26 | | -0.0063 | | 0.0042 | | 0.21 | |  | |
| Amygdala | -0.0272 | | 0.0246 | | 0.36 | | 0.0044 | | 0.0040 | | 0.36 | | 0.0037 | | 0.0040 | | 0.47 | |  | |
| Caudate | -0.0032 | | 0.0260 | | 0.90 | | 0.0036 | | 0.0050 | | 0.48 | | 0.0033 | | 0.0049 | | 0.50 | |  | |
| Hippocampus | -0.0595 | | 0.0250 | | 0.069 | | -0.0107 | | 0.0045 | | **0.045** | | -0.0124 | | 0.0044 | | **0.013** | |  | |
| Pallidum | -0.0284 | | 0.0242 | | 0.36 | | -0.0094 | | 0.0045 | | 0.071 | | -0.0097 | | 0.0044 | | 0.055 | |  | |
| Putamen | -0.0081 | | 0.0246 | | 0.85 | | 0.0034 | | 0.0048 | | 0.48 | | 0.0033 | | 0.0047 | | 0.50 | |  | |
| Thalamus | -0.0281 | | 0.0209 | | 0.36 | | -0.0170 | | 0.0040 | | **9.54E-05** | | -0.0166 | | 0.0040 | | **1.03E-04** | |  | |
| Ventral DC | -0.0576 | | 0.0220 | | 0.069 | | -0.0238 | | 0.0040 | | **2.59E-08** | | -0.0249 | | 0.0040 | | **2.42E-09** | |  | |
|  |  | |  | |  | |  | |  | |  | |  | |  | |  | |  | |
|  | |  | |  | |  | |  | |  | |  | |  | |  | |  | |  |
| **Neglect Composite Score** | | **GS (n=1,024)** | | | | | | **UKB (n=27,202)** | | | | | | **Mega-Analysis (n=28,226)** | | | | | |  |
| **Region** | | **Beta** | | **Std Err** | | **P(FDR)** | | **Beta** | | **Std Err** | | **P(FDR)** | | **Beta** | | **Std Err** | | **P(FDR)** | |  |
| Accumbens | | -0.0805 | | 0.0229 | | **0.0037** | | 0.0001 | | 0.0042 | | 0.99 | | -0.0031 | | 0.0041 | | 0.52 | |  |
| Amygdala | | 0.0066 | | 0.0244 | | 0.79 | | -0.0009 | | 0.0040 | | 0.94 | | -0.0007 | | 0.0039 | | 0.85 | |  |
| Caudate | | -0.0122 | | 0.0257 | | 0.79 | | 0.0142 | | 0.0050 | | **0.018** | | 0.0131 | | 0.0049 | | **0.030** | |  |
| Hippocampus | | -0.0430 | | 0.0247 | | 0.31 | | -0.0064 | | 0.0044 | | 0.24 | | -0.0080 | | 0.0044 | | 0.14 | |  |
| Pallidum | | -0.0146 | | 0.0239 | | 0.79 | | -0.0053 | | 0.0044 | | 0.31 | | -0.0060 | | 0.0044 | | 0.22 | |  |
| Putamen | | -0.0103 | | 0.0243 | | 0.79 | | 0.0112 | | 0.0048 | | **0.049** | | 0.0102 | | 0.0047 | | 0.077 | |  |
| Thalamus | | -0.0054 | | 0.0207 | | 0.79 | | -0.0062 | | 0.0040 | | 0.24 | | -0.0060 | | 0.0039 | | 0.20 | |  |
| Ventral DC | | -0.0341 | | 0.0217 | | 0.31 | | -0.0136 | | 0.0040 | | **0.0055** | | -0.0146 | | 0.0039 | | **0.0017** | |  |
|  | |  | |  | |  | |  | |  | |  | |  | |  | |  | |  |
